# The neurotrophin DNT-2 via the Toll-2 receptor regulates neuronal survival and morphology during visual system development

**DOI:** 10.1242/bio.062837

**Published:** 2026-09-17

**Authors:** Naser Alshamsi, Francisca Rojo-Cortés, Jervis Fernandes, Callum Perrett, Bangfu Zhu, Samaher Fahy, Guiyi Li, Anna Lassota, Marta Moreira, Alicia Hidalgo

**Affiliations:** Birmingham Centre for Neurogenetics, School of Biosciences, University of Birmingham, Birmingham B15 2TT, UK

**Keywords:** Neurotrophin, DNT, Spätzle, Spz, Toll receptor, Cell survival, Cell death, Cell number, Dendrite, Visual system

## Abstract

During vertebrate nervous system development, neurons are produced in excess, and those receiving neurotrophin ligands are maintained, enabling neural circuit establishment. An apoptotic wave sweeps across the *Drosophila* pupal visual system, but whether neurotrophins participate in forming adult visual circuits remains unknown. Here, we show that *Drosophila neurotrophin-3* (*spz-3*) and *DNT-2* (*spz-5*) are expressed in retinal cells and medulla neurons and *Toll* receptors across the visual system. Using loss- and gain-of-function conditions for *DNT-3* (*spz-3*) and *DNT-2* (*spz-5*), we show that they both can, and are required to, promote cell survival. Importantly, genetic interaction data show that DNT-2 can function together with Toll-2. DNT-2 neurons were identified as medulla Mi1 neurons that connect to lamina L1 neurons expressing Toll-2. Loss of function for *DNT-2* or *Toll-2* induced apoptosis, and *Toll-2* knock-down prevented the pro-survival function of DNT-2. *DNT-2* overexpression resulted in excess Toll-2 neurons, whereas most Toll-2 neurons were lost in *DNT-2* mutants. Furthermore, DNT-2 and Toll-2 were required for appropriate L1 axonal columnar organisation and dendritic morphology. Altogether, evolutionarily conserved neurotrophin family ligands control neuronal number through Toll receptors during visual circuit development in *Drosophila*.

## Teaser

**Summary:** *Drosophila* neurotrophin-2 (DNT-2) with the Toll-2 receptor regulates neuronal survival, axonal columnar organisation and dendritic morphology during pupal visual system development, revealing deep evolutionarily conserved neurotrophism in fruit flies.

## INTRODUCTION

Neural circuits emerge during development, raising the question of how the required number of neurons is established to ensure appropriate brain function and behaviour. In vertebrates, neurons are generated in excess, surplus cells are eliminated by apoptosis, and survival is maintained by neurotrophic factors secreted in limiting amounts by target cells (Davies, 2003; Levi-Montalcini, 1987). Glial survival similarly depends on neuron-derived gliatrophic factors (Raff et al., 1993). In this way, only neurons that receive trophic support as they approach their targets are maintained, and cell number is adjusted across interacting cell populations (Levi-Montalcini, 1987; Raff et al., 1993). The neurotrophins NGF, BDNF, NT3 and NT4 are the key mediators of vertebrate neurotrophism – the adjustment in neuronal survival during neural circuit formation – and function as ligands for Trk receptor signalling via ERK and AKT downstream and for the p75^NTR^ receptor via NF-κB downstream (Lu et al., 2005). Neurotrophins can also promote cell death, depending on context, via p75^NTR^, Sortilin, and JNK signalling instead (Lu et al., 2005). Neurotrophin and receptor homologues have been found across invertebrates (Benito-Gutierrez et al., 2005; DeLotto and DeLotto, 1998; Foldi et al., 2017; Hallbook, 1999; Laramore et al., 2011; Lauri et al., 2016; McIlroy et al., 2013; Zhu et al., 2008; Mandai et al., 2009; Ulian-Benitez et al., 2017), but functional *in vivo* evidence for their involvement in neurotrophism outside fruit-flies remains limited.

In *Drosophila*, *Drosophila* neurotrophins (DNTs) are encoded by *spätzle* (*spz*) paralogue genes, which include sequence, structural and functional neurotrophin homologues (DeLotto and DeLotto, 1998; Foldi et al., 2017; Zhu et al., 2008; Sun et al., 2024; Coutinho-Budd et al., 2017; Ballard et al., 2014; Beachum et al., 2025; Parker et al., 2001). DNT/Spz ligands bind Kekkon (Kek) receptors – kinase-less Trk-family members – and Toll receptors (Foldi et al., 2017; Ulian-Benitez et al., 2017; Weber et al., 2003; Mandai et al., 2009; McIlroy et al., 2013). There are six *spz* and nine *Toll* paralogues in *Drosophila* (Parker et al., 2001; Tauszig et al., 2000). Spz-1 is the well-known ligand of Toll-1 (also known as Toll), DNT-1 (also known as Spz-2) and DNT-2 (also known as Spz-5) of Toll-7 and Toll-6, respectively, and Spz-3 is a candidate ligand for Toll-8 (also known as Tollo) (Ballard et al., 2014; McIlroy et al., 2013; Weber et al., 2003). Mature DNT-1 and DNT-2 and their Toll-6 and Toll-7 receptors can promote neuronal survival in embryonic, pupal and larval ventral nerve cords and in adult brains, demonstrating functional evolutionary conservation with vertebrate neurotrophins (Foldi et al., 2017; Li et al., 2020; Sun et al., 2024; Zhu et al., 2008; McIlroy et al., 2013). Spz-3 also has neurotrophin functions (Coutinho-Budd et al., 2017; Beachum et al., 2025; Ballard et al., 2014), and therefore, we refer to it as DNT-3. Importantly, at least full-length DNT-1 and Toll-1 can promote cell death instead, and Toll-6 can promote either cell survival or death, depending on the cell context (Foldi et al., 2017; Singh et al., 2025; Zhu et al., 2008).

The developing visual system offers an ideal context in which to further test neurotrophin function in *Drosophila*. The optic lobe develops from outer and inner proliferation centres, where transcription factor cascades and lineage-based neurogenesis generate distinct lamina, medulla, lobula and lobula plate neurons that assemble into circuits (Bakshi et al., 2025; Malin and Desplan, 2021; Ngo et al., 2017). Photoreceptors R1–R6 project to the lamina and form cartridges with L1–L5 neurons, R7 and R8 project to medulla layers M6 and M3, and medulla interneurons connect across layers to process motion, colour and other visual features (Tuthill et al., 2014; Behnia and Desplan, 2015; Borst and Groschner, 2023; Matsliah et al., 2024; Schnaitmann et al., 2020). The different layers of the optic lobe correspond to functionally distinct stages of visual processing in which medulla interneurons play central roles in routing and transforming feature-specific information (Behnia and Desplan, 2015; Borst and Groschner, 2023). Neurons within the lobula complex integrate signals from the medulla and project to the optic glomeruli in the central brain and motor outputs to enable appropriate behaviour (Klapoetke et al., 2022; Tanaka and Clark, 2022; Wu et al., 2016). During neural circuit formation, a wave of cell death takes place in the pupal optic lobe (Holguera and Desplan, 2018; Togane et al., 2012). Apoptosis is partly induced by ecdysone, but there is also extensive ecdysone-independent cell death (Hara et al., 2013). Apoptosis peaks at 24 h after puparium formation (APF) and declines at 48 h APF (Togane et al., 2012; Hara et al., 2013). Critically, visual circuit assembly overlaps with this temporal window, culminating in synaptogenesis and spontaneous neuronal activity starting from 48 h APF and peaking at 72 h APF, in preparation for adult eclosion at 96 h APF (Hadjieconomou et al., 2011; Melnattur and Lee, 2011; Millard and Pecot, 2018; Akin et al., 2019; Bajar et al., 2022). The temporal correlation between cell death and neural circuit formation prompted us to ask whether neurotrophins regulate cell survival during visual system development in *Drosophila*.

Here, we show that DNT-2 (Spz-5) and DNT-3 (Spz-3) are expressed in the retina and medulla and Toll receptors in the lamina and throughout the optic lobe. Both DNTs maintain cell survival in the pupal visual system. Genetic evidence suggests that DNT-2 functions with Toll-2, in addition to its established receptors Toll-6 and Kek-6 (McIlroy et al., 2013; Ulian-Benitez et al., 2017). *DNT-2* is expressed in Mi1 medulla neurons, *Toll-2* is expressed in connecting L1 lamina neurons, and DNT-2 functions through Toll-2 to maintain neuronal survival and regulate axonal columnar organisation and dendritic morphology. Together, these findings show that evolutionarily conserved neurotrophism participates in visual system development in *Drosophila*.

## RESULTS

### Differential expression of *spz* paralogues and *Toll* receptors in the developing optic lobe

To ask whether *DNTs/spzs* are expressed in the pupal optic lobe, we generated *T2A-Gal4* driver lines for *spz-1*,*3*,*4*,*5*, crossed them to *10xUASmyrGFP* or *20UAS6xmCherry* reporter flies, and used anti-GFP or anti-DsRed antibodies, as required (Fig. 1). These tools reveal the cells expressing these *spz*-family genes. *spz-1^MIO2318^-T2A>myrGFP* and *spz-1^MIO2318^-T2A>6xmCherry* revealed expression in a few centrifugal neurons in the lobula complex that projected to the lamina (48 h APF), in medulla neurons (48 h APF), and in lobula complex neurons and central brain neurons projecting into the lobula complex (72 h APF) (Fig. 1A). Expression from *spz4^MI5678^-T2A->myrGFP* was not detected until 72 h APF, and both at this time point and in the adult brain, it was found in the medulla, in the lobula complex and prominently in the trachea (Fig. 1B). *spz-3-T2A>6xmCherry* (hereby named *DNT-3*) was highly expressed in non-neuronal retinal cells and medulla neurons (24 and 48 h APF) and in the trachea and other cells that, based on morphology, could perhaps be glia at 72 h APF (Fig. 1C). *DNT-2-T2A>6xmCherry* (also known as *spz-5*) was expressed in medulla neurons and retinal cells (24–72 h APF) (Fig. 1D, and see below). Focusing on somas revealed abundant cells expressing *spz-1* throughout the optic lobe (Fig. 1E, 48 and 72 h) and *DNT-3/spz-3* and *DNT-2/spz-5* (Fig. 1F,G, respectively) also in the optic lobe. *DNT-3/spz-3* and *DNT-2/spz-5* were also expressed in retinal cells, seen in MultiColor FlpOut (MCFO) clones (Fig. 1H–M). *spz-3>mCherry* expression did not overlap with the photoreceptor marker N-cadherin, and based on morphology, Spz-3+ cells appear to be secondary pigment cells instead (Fig. 1H,I,N). *DNT-2>mCherry* did not overlap with N-cadherin either (Fig. 1K), but the cells had long projections, meaning they are most likely neurons (Fig. 1L,M). The projections did not target the lamina or medulla (Fig. 1M) and, based on morphology and location (Fig. 1J–N), appeared to be interommatidial bristle neurons (Longley and Ready, 1995; Garrido-Garcia et al., 2026).

**Fig. 1.**
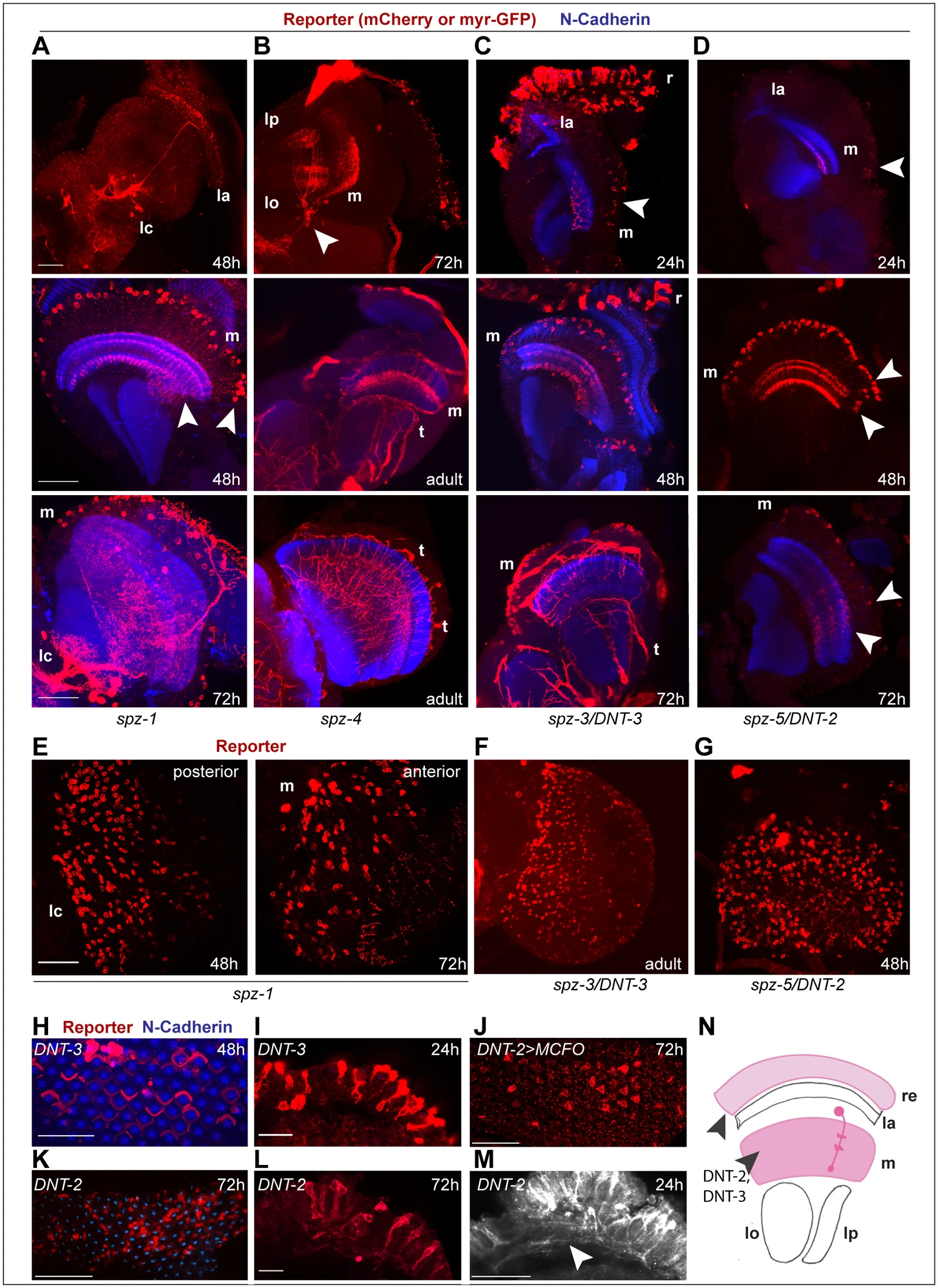
**Expression of *spz*-family ligands in the optic lobe.** (A) *spz-1^[MIO2318]^-T2A-Gal4>myrGP* (top) and *spz-1^[MIO2318^-]T2A-Gal4>20UAS6xmCherry* (middle and bottom) show expression in the lobula complex (lc) and medulla (m, arrowheads); top and bottom images: horizontal views; middle: vertical view. (B) *spz-4^[MI5678]^T2A-Gal4>myrGFP*-expressing cells in the lobula complex (arrowhead), projecting into the medulla and prominent in the trachea (t). Vertical view images; bottom image: horizontal view. (C) *spz-3-T2A-Gal4>20UAS6xmCherry* in the retina (r) and medulla (arrowhead) and possibly the trachea (vertical views). (D) *DNT-2-T2A-Gal4>20UAS6xmCherry* expression in medulla neurons (arrowheads, vertical views). (E–G) Cell bodies in the medulla and lobula complex expressing (E) *spz-1^[MIO2318]^-T2A-Gal4>20UAS6xmCherry*, showing anterior and posterior focal planes; (F) *spz-3-T2A-Gal4>20UAS6xmCherry*; and (G) *DNT-2-T2A-Gal4>20UAS6xmCherry*. (H–M) Retinal images showing (H,K) no colocalisation between *spz-3/DNT-3-T2A-Gal4>20xUAS6xmCherry* or *spz-5/DNT-2-T2A-Gal4>20xUAS6xmCherry* and the photoreceptor marker anti-N-cadherin. (I) N-cadherin–Spz-3+/DNT-3+ cells could be pigment cells. (J–M) Spz-5+/DNT-2+ cells could be inter-ommatidial bristle neurons, with long projections (M, arrowhead). Stages on images are APF. (N) Diagram illustrating expression domains of *DNT-2* and *DNT-3* (pink), in the medulla and retina, with an example of a medulla neuron. Scale bars: (A–H,J,K,M) 50 µm; (I,L) 20 µm.

Spz-1 and DNT-2 are known ligands for Toll-1 and Toll-6 receptors, respectively (Weber et al., 2003; McIlroy et al., 2013), and DNT-3 (Spz-3) is a candidate ligand for Toll-8 (Ballard et al., 2014). *Tolls* are expressed in the optic lobes of adult flies (Li et al., 2020). To visualise the expression of *Tolls* in pupal optic lobes, we used GAL4 lines previously described (Li et al., 2020), driving *myrGFP* expression (Fig. 2A–D). *Toll-1-T2A>myrGFP* initially revealed prominent expression in all optic neuropiles, including the retina, and subsequently was prominent in the medulla and lobula complex (Fig. 2A). *Toll-8^MD806^>myrGFP* expression was prominent in the lobula complex and included some medulla neurons and lamina neurons (Fig. 2B). However, this P-element-driven Gal4 insertion 180 bp upstream of the start codon may not faithfully represent the endogenous expression pattern of *Toll-8*. *Toll-6^MIO2127^>myrGFP* was initially prominently expressed in the lobula complex but included lamina and medulla neurons, and subsequently, the expression became more prominent in the lamina (Fig. 2C). *Toll-2^PTV^>myrGFP* was initially found in photoreceptors and lamina, medulla and lobula complex neurons, and by 72 h APF, *Toll-2^PTV^>myrGFP* expression was prominent in lamina neurons projecting to the medulla (Fig. 2D). Using MCFO clones as well as *myrGFP*, we could identify Toll-8+ neurons as Lawf1 (Fig. 2E) feedback neurons projecting from the medulla to the lamina, L4 lamina neurons (Fig. 2F,J) and other unidentified lamina neurons (Fig. 2J′); Toll-6+ cells as including L3 and L4 lamina neurons (Fig. 2G,K,K′); and Toll-2+ neurons as L1 lamina neurons which project to M1 and M5 medulla layers and L3 lamina neurons that project to M3 (Fig. 2H,I,L,L′) (Fischbach and Dittrich, 1989).

**Fig. 2.**
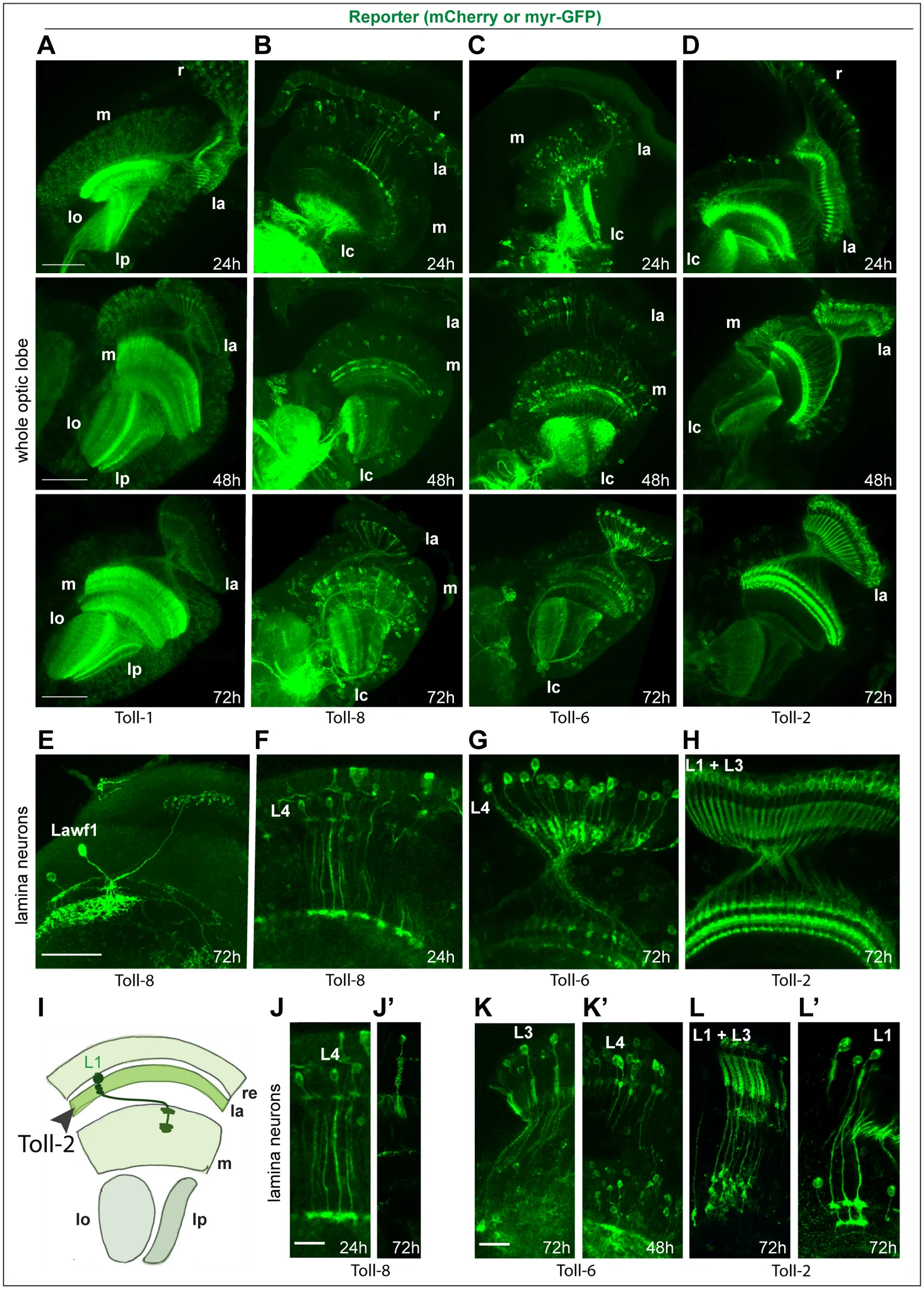
**Expression of *Toll* receptors in the pupal optic lobe.** (A) *Toll-1-T2A-Gal4>myrGFP* is expressed in the retina and all optic lobe neuropiles, throughout development. (B,F) *Toll-8^MD806^GAL4>myrGFP* is expressed predominantly in the lobula complex (lc), plus some lamina (la) and medulla (m) neurons. (C,G) *Toll-6^MIO2127^Gal4>myrGFP* is initially expressed in the lobula complex, subsequently also in lamina and medulla neurons. (D,H) *Toll-2^pTV^Gal4>myrGFP* expression in the retina and lamina, medulla and lobula complex neurons. *Toll-8^MD806^GAL4>MCFO* (E,J′), *Toll-6^MIO2127^>MCFO* (K), and *Toll-2^pTV^>MCFO* (L,L′): MCFO clones stained with anti-HA antibodies and (F,G,H,J,K′) higher-magnification views of *myrGFP* samples stained with anti-GFP antibodies suggest potential expression of *Toll-8* in the Lawf1 medulla and L4 lamina neurons; *Toll-6* in L3 and L4 lamina neurons; and *Toll-2* in L1 and L3 lamina neurons. (I) Diagram illustrating expression of Toll receptors (green), including in the lamina (darker green), with one example of a lamina neuron. Timings are APF. Scale bars: (A–H) 50 µm; (J–L′) 20 µm.

We compared our reporter-based profiles with published single-cell RNA sequencing (scRNA-seq) datasets of the optic lobe through development (Kurmangaliyev et al., 2020) (Figs S1–S7). In scRNA-seq, *spz-*family expression was low and dynamic, whereas *Toll* receptors were expressed more abundantly and widely, consistent with the reporter profiles shown in Figs 1 and 2. Regarding cell types, the expression in the scRNA-seq dataset (Kurmangaliyev et al., 2020) of the *spz* ligands was less consistent with the *spz-*paralogue translational reporters (in Fig. 1) than the expression of *Tolls* (Fig. 2). In fact, the expression of *Toll-1*, *Toll-2* (also known as *18w*) and *Toll-6* confirmed cells seen with the transcriptional (*Toll-2*, *Toll-6*) and translational (*Toll-1*) reporters shown in Fig. 2. The exception was a discrepancy in *Toll-8* (also known as *Tollo*), which can be explained by the Gal4 insertion (see above). Most importantly, *Toll-2* mRNA (synonym *18w*) was found in L1, L3 and L5 at 24 h APF; and *Toll-6* mRNA was found in L1–L4 at 24 h (Figs S1–S6). Focusing on *DNT-2* (*spz-5*) and *Toll-2* (*18w*), *DNT-2* mRNA was found in Mi1 neurons at 48 h and in clusters (some unclassified) which could include other medulla neurons (Fig. S7). Between 24 and 48 h APF, *Toll-2* was highly expressed in L1 and L3 lamina neurons, as well as in L4 and L5 and in various medulla neurons (Fig. S7), consistent with reporter-based data (Fig. 2D,H,L,L′). From 72 h APF onwards, *Toll-2* expression decreased in L1 and increased in L3 neurons. The discrepancies between the reporter and scRNA-seq data can be explained as scRNA-seq reveals only a temporal snapshot within a dynamic profile, transcription does not necessarily convert into translation, translational reporters have a temporal lag relative to scRNA-seq, and GAL4/UAS amplifies signals. Importantly, scRNA-seq analysis showed that *Toll-2* was expressed in cells (L1, Mi1, Tm3, Dm9, T4) that could receive the DNT-2 ligand produced from medulla neurons (Fig. S7), as DNT-2 is well known to be secreted and to function non-autonomously (Foldi et al., 2017; McIlroy et al., 2013; McLaughlin et al., 2019; Sun et al., 2024; Ulian-Benitez et al., 2017).

Altogether, at the time of naturally occurring cell death (0–48 h APF), *Toll-1* is highly expressed throughout the optic lobe; *Toll-2*, *Toll-6* and *Toll-8* are expressed in the medulla; *Toll-8* and *Toll-6* are expressed in the lobula complex; and *Toll-6* and *Toll-2* are prominently expressed in the lamina. Interestingly, each lamina neuron type expresses distinct *Tolls* or a combination of *Tolls*: L1 neurons express *Toll-2* (Fig. 2D,H,I,L,L′); L2 may express *Toll-6* and *Toll-8* (scRNA-seq, Fig. S1); L3 neurons express *Toll-2* and *Toll-6* (Fig. 2H,I,K,L); and L4 neurons express at least *Toll-6* (Fig. 2G,K′).

The data tentatively suggested that *DNT-2* might be expressed in Mi1 medulla neurons that – according to the connectome (Nern et al., 2025) – interact with L1 lamina neurons expressing *Toll-2*. To test this, we used the marker anti-Bsh, which labels Mi1 neurons (Xu et al., 2024a). There was clear colocalisation of Bsh in DNT-2>6xmCherry cells (Fig. 3A–C), demonstrating that *DNT-2* is expressed in Mi1 neurons. Next, we used the L1 marker anti-Svp (Xu et al., 2024b) and the L3 marker anti-Erm (Hildebrandt et al., 2021) in Toll-2>hisHYFP-labelled cells. Unambiguous colocalisation of Toll-2>His-YFP with Svp in all Svp+ lamina cells (Fig. 3D,E) and with a subset of Erm cells (Fig. 3F,G) demonstrated that *Toll-2* is expressed in all L1 and some L3 neurons. The data show that *DNT-2* is expressed in Mi1 medulla neurons and *Toll-2* in L1 lamina neurons.

**Fig. 3.**
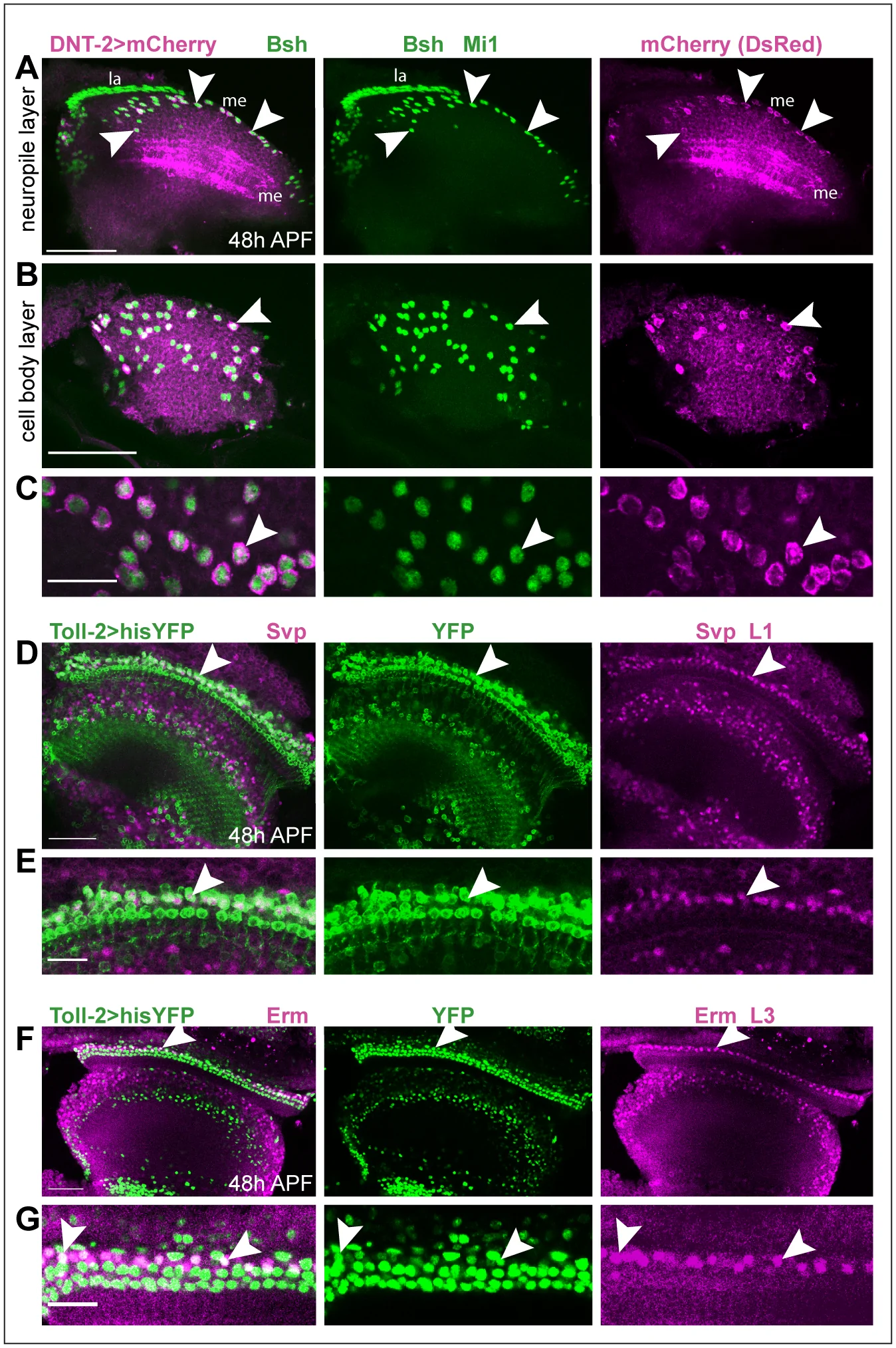
**Identification of *DNT-2*- and *Toll-2*-expressing neurons.** (A–C) *DNT-2>mCherry* neurons visualised with anti-DsRed antibodies colocalise the Mi1 marker anti-Bsh (arrowheads) at higher magnification in C. (D,E) Nuclei of Toll-2>Histone-YFP neurons colocalise with the L1 marker anti-Svp (arrowheads) at higher magnification in E. (F,G) Toll-2>hisYFP neurons colocalised with the anti-Erm L3 lamina neuron marker (arrowheads), at higher magnification in G. Stage: all 48 h APF. Scale bars: (A,B,D,F) 50 µm; (C,E,G) 20 µm.

According to the connectome, L1 and Mi1 neurons are synaptic partners (Nern et al., 2008). To confirm the identity of *DNT-2*- and *Toll-2*-expressing neurons, we used GFP reconstitution across synaptic partners (GRASP) (Feinberg et al., 2008) to test if these cells could connect. Here, the GFP protein is split into two halves, and fluorescence is restored only when neurons expressing one half of GFP at the synapse, with a synaptobrevin–GFP1-10 protein fusion (with LexAOP), contact neurons expressing half-GFP11 postsynaptically (with UAS) (Feinberg et al., 2008). Samples were counterstained with the neuropile marker N-cadherin. Driving GRASP with *Toll-2LexA* and *DNT-2Gal4* revealed a clear signal along the medulla neuropile, which was absent in controls expressing GRASP but lacking the *Toll-2LexA* and *DNT-2Gal4* drivers (Fig. 4A,B). This was consistent with DNT-2+ medulla neurons contacting or being in close proximity to Toll-2+ lamina neurons. We also observed GRASP signals along the lamina, consistent with *DNT-2* expression in the retina (Fig. 4A,B), suggesting that retinal DNT-2+ neurons can also reach lamina Toll-2+ neurons. Altogether, these data confirm that DNT-2+ cells in the retina and medulla either connect with or are in close proximity to Toll-2+ neurons (Fig. 4C).

**Fig. 4.**
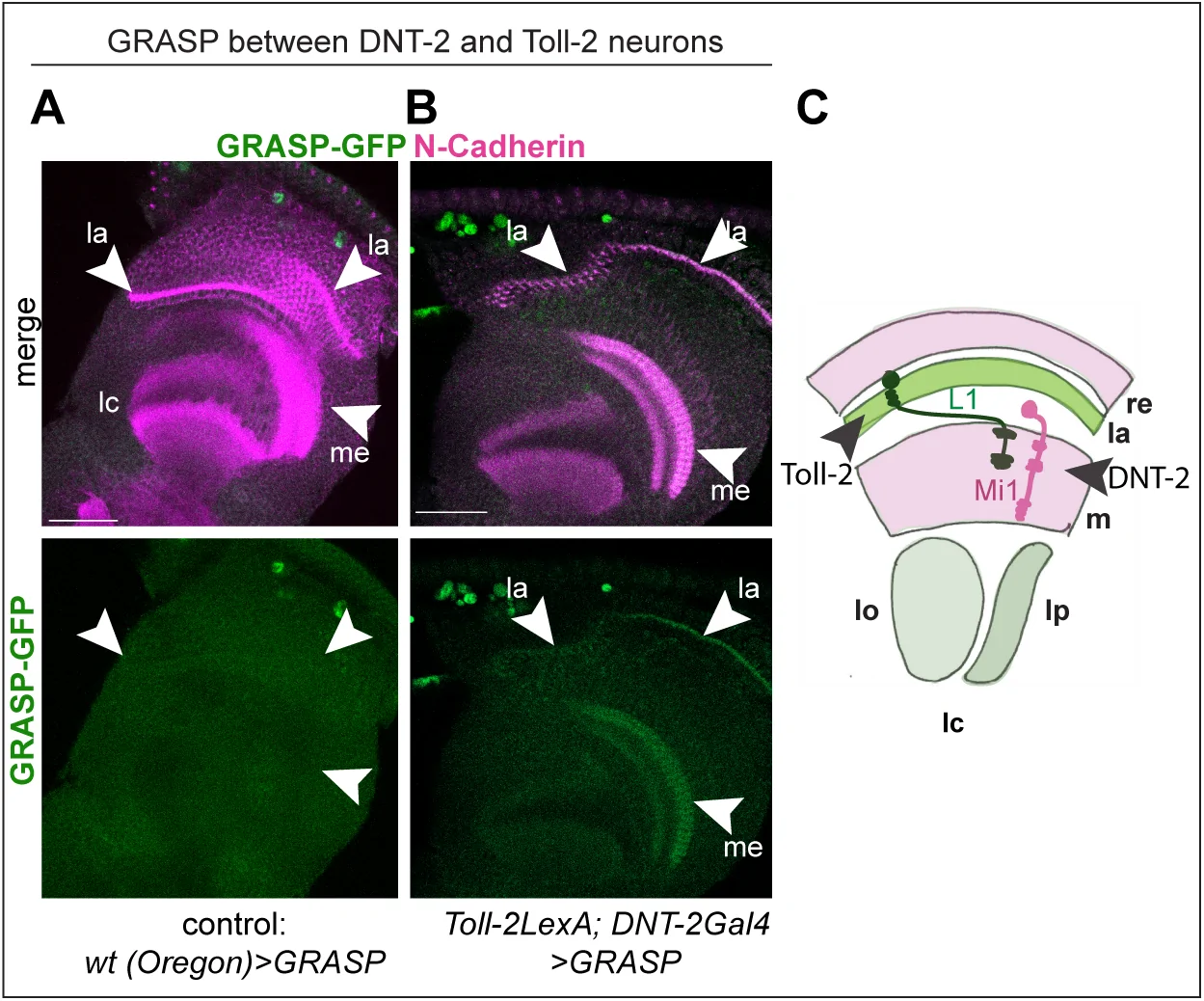
**GRASP+ signal between DNT-2 and Toll-2 neurons.** (A) Controls, carrying the UAS-GRASP/+ reporter but not drivers lacking the GFP signal (72 h APF). (B) Reconstituted GFP signal from presynaptic *LexAOP-nsyb-spGFP1-10* and postsynaptic *UAS-CD4-spGFP11* driven by *Toll-2LexA;DNT-2Gal4* and visualised with GRASP-specific anti-GFP antibodies revealed proximity between DNT-2 and Toll-2 neurons at 24 h APF. Arrowheads indicate lamina (la) and medulla (me) neuropiles (lc: lobula complex; lo: lobula; lp: lobula plate) counterstained with N-cadherin. (C) Diagram illustrating *DNT-2* (pink) and *Toll-2* (green) expression and the relative positions of one L1 and one Mi1 neuron. Scale bars: (A,B) 50 µm.

Transcriptional and translational profiles do not reveal where ligands are secreted from or the subcellular distribution of Toll receptors. Notwithstanding these limitations and given previous evidence that DNT-2 is naturally cleaved and secreted (Foldi et al., 2017; McIlroy et al., 2013), the above data indicate that DNT-2 could be secreted from retinal cells and Mi1 neurons and that the Toll-2 receptor is distributed in, at least, L1 lamina and medulla neurons.

### The neurotrophins DNT-2 and DNT-3 promote neuronal survival during optic lobe development

To ask whether *DNTs* can promote cell survival in developing optic lobe development, we focused on *DNT-3/spz-3* and *DNT-2/spz-5*, as they were both expressed throughout the period of naturally occurring cell death (0–48 h APF). DNT-2 (Spz-5) promotes cell survival in the embryonic central nervous system (CNS) and in adult brains, cleaved DNT-1 (Spz-2) promotes cell survival in the embryonic CNS, and DNT-3 (Spz-3) promotes cell survival in the adult brain, and both DNT-2 and DNT-3 also have neurotrophin functions at the neuromuscular junction (Zhu et al., 2008; Coutinho-Budd et al., 2017; Sun et al., 2024; Ballard et al., 2014; Ulian-Benitez et al., 2017; Beachum et al., 2025; Sutcliffe et al., 2013). We first confirmed that *DNT-2* is expressed during the period of naturally occurring cell death in the optic lobe, by staining apoptotic cells with anti-Dcp1 concomitantly with visualising *DNT-2>mCherry* at 24 h APF (Fig. 5A–A″). Remarkably intense Dcp1 was observed across the lamina in wild-type optic lobes (Fig. 5A,A′). In fact, counterstaining control optic lobes with the neuropile marker anti-N-cadherin revealed Dcp1-labelled cells abutting the N-cadherin+ lamina neuropile (Fig. 5B,B′). In horizontal mounts, the lamina lies between the medulla and the retina and can be clearly seen from the tissue fold edges (Fig. 5A′,D). Thus, we asked whether DNT-2 and DNT-3 are required to maintain cell survival during optic lobe development and measured the volume occupied by Dcp1 signals throughout the stack of confocal images. We generated *DNT-3/spz-3* loss-of-function mutants by P-element mobilisation, and we previously published *DNT-2* mutants (Foldi et al., 2017; Sun et al., 2024; Ulian-Benitez et al., 2017). *DNT-2^18^/DNT-2^37^* and *DNT-3^46^/DNT-3^27^* loss-of-function mutants displayed very high levels of fine Dcp1+ cell debris (presumably resulting from fragmenting dying cells), which prevented accurate quantification in the medulla and lobula complex, so we focused on the lamina (Fig. 5C). We found that in *DNT-3^46^/DNT-3^27^* (i.e. *spz-3^46^/spz-3^27^*) pupae, Dcp1+ signals increased in the lamina compared to wild-type controls at 24 h APF (Fig. 5C,F). Similarly, in null *DNT-2^18^/DNT-2^37^* mutants, Dcp1 also increased in the lamina (Fig. 5C,F). These data show that both *DNT-2* and *DNT-3* are required to maintain cell survival of lamina cells during optic lobe development.

**Fig. 5.**
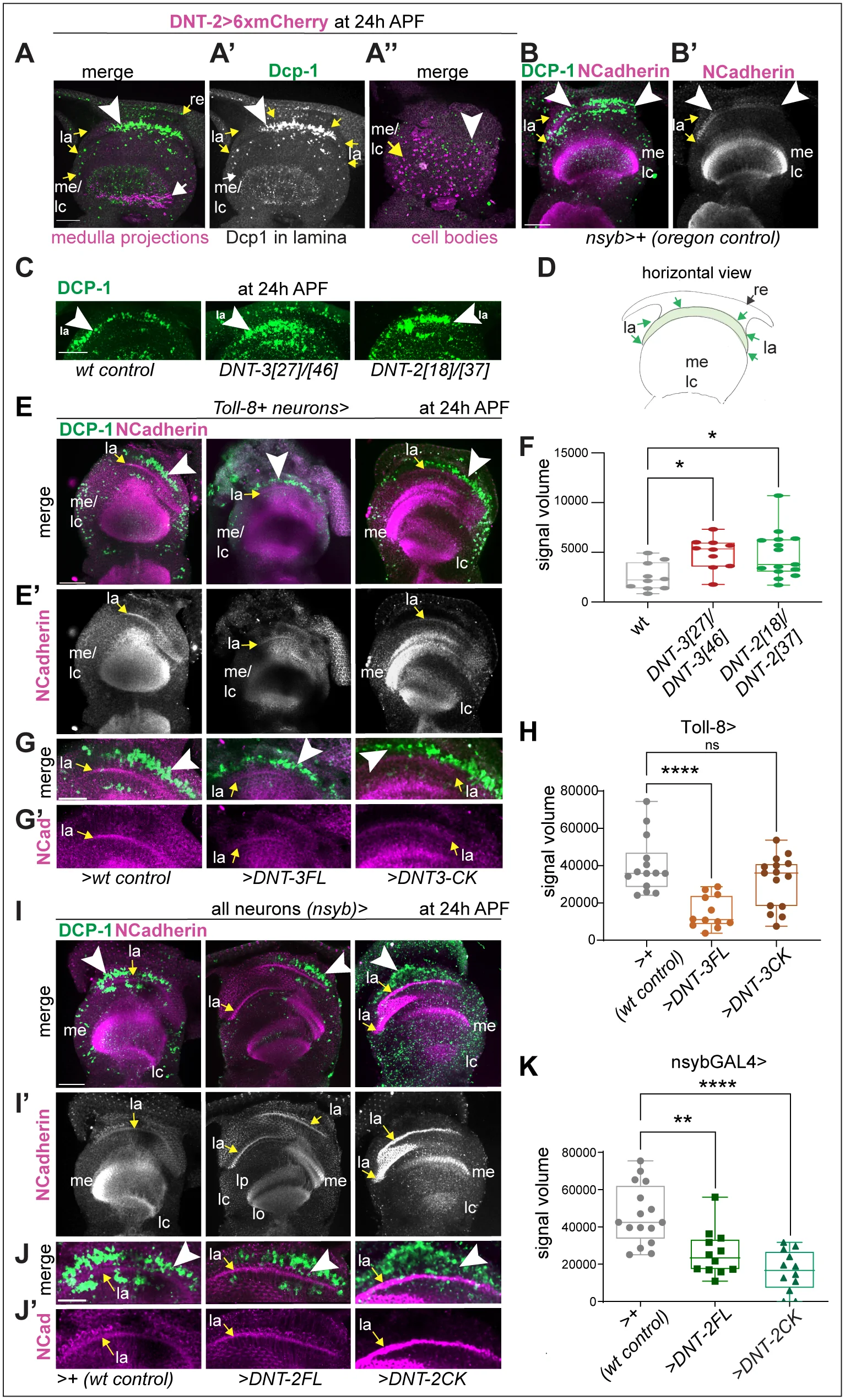
**DNT-2 and DNT-3 maintain cell survival during optic lobe development.** (A–A″) Apoptotic cells labelled with anti-Dcp1 concomitantly with *DNT-2>mCherry* at 24 h APF, at the lamina (A,A′, la, yellow arrows) and at the medulla and lobula complex (me/lc, A″). The lamina (la) fold is sandwiched between the medulla (me) and retina (re) in horizontal views (yellow arrows) (A′), illustrated in D. (B,B′) Abundant Dcp1+ cells abutting the lamina neuropile, visualised with N-cadherin. (C,F) *DNT-3^46^/DNT-3^27^* (i.e. *spz-3^46^/spz-3^27^*) and *DNT-2^18^/DNT-2^37^* (i.e. *spz-5^18^/spz-5^37^*) trans-heterozygous loss-of-function mutants had increased lamina apoptosis at 24 h APF, quantification in F: one-way ANOVA ****P<0.0001 and Dunnett's multiple-comparison correction to a fixed control. (D) Illustration of the lamina (green) relative to the retina and medulla in horizontal views of the optic lobe at 24 h APF. (E,E′,I,I′) Projection of the optical section subset showing Dcp1+ cells just over the lamina neuropile labelled with N-cadherin (yellow arrows, la), which also labels medulla (me) and lobula complex (lc) neuropiles; higher magnification in G, G′, J, J′. Overexpression of (E,E′,G,G′,H) *spz-3/DNT-3* in Toll-8 neurons (*Toll-8>spz-3/DNT-3*) and (I,I′,J,J′,K) *DNT-2/spz-5* in all neurons (*nsyb>DNT-2*) reduced Dcp1+ apoptosis in the lamina (white arrowheads), identified with N-cadherin at 24 h APF (yellow arrows). (H,K) One-way ANOVA ****P<0.0001 and Dunnett's multiple-comparison correction to a fixed control. FL, full length; CK, cystine-knot domain. A,B,E,G,I,J, composite images; B′,E′,G′,I′,J′, the N-cadherin channel only. (F,H,K) Quantification of the Dcp1 lamina signal volume through the entire stack of optical sections at the lamina. Asterisks indicate multiple-comparison corrections: *P<0.05, **P<0.01, ***P<0.001, ****P<0.0001. For further details, see Table S4. Scale bars: (A,A′,A″,B,B′,C,E,E′,I,I′) 50 µm; (G,G′,J,J′) 30 µm.

Next, we asked whether *DNT-2/spz-5* and *DNT-3/spz-3* could promote cell survival, by overexpressing *DNT-2/spz-5* and *DNT-3/spz-3* and visualising dying cells with anti-Dcp1 at 24 h APF. Spz-family ligands, like mammalian neurotrophins, can be found in full-length or cleaved forms consisting of the evolutionarily conserved neurotrophin cystine-knot domain (Foldi et al., 2017; Zhu et al., 2008; DeLotto and DeLotto, 1998; Hu et al., 2004). Overexpressed cystine-knot forms from transgenic flies may not be secreted, but they are functional upon *in vivo* overexpression, where they act cell-autonomously (Foldi et al., 2017; Sun et al., 2024; Zhu et al., 2008; Ulian-Benitez et al., 2017; Hu et al., 2004; Ligoxygakis et al., 2002). By contrast, overexpressed full-length forms of *DNT-2* and *DNT-3* are spontaneously cleaved and secreted both in cell culture and *in vivo*, thus enabling non-autonomous activation of their receptors (McIlroy et al., 2013; Foldi et al., 2017; Sun et al., 2024; Coutinho-Budd et al., 2017). Both *DNT-2* and *DNT-3* are expressed in retinal cells and medulla neurons, which could non-autonomously influence neurons in the lamina, medulla and lobula complex. As Toll-8 is the proposed receptor for DNT-3/Spz-3 (Ballard et al., 2014), we overexpressed *spz-3* in Toll-8+ neurons, labelled apoptotic cells with Dcp1 and counterstained the optic lobes with N-cadherin to identify the distinct neuropiles. We initially focused on the Dcp1 signal abutting the N-cadherin+ lamina neuropile (Fig. 5B,D). Overexpression of *spz-3/DNT-3-full length* (*DNT-3FL*) but not cleaved *DNT-3 cystine-knot* (*DNT-3 CK*) in Toll-8+ cells (i.e. *Toll-8>DNT-3FL* and *Toll-8>DNT-3CK*, respectively) reduced the incidence of Dcp1+ apoptosis in the lamina (Fig. 5E–G′,H). *Spz-3FL/DNT-3FL* is secreted (Coutinho-Budd et al., 2017), whereas *DNT-3CK* may not be, and it might be unable to rescue cell survival cell-autonomously due to the weak and irregular expression of *Toll-8* in the lamina with this Gal4 line. Overexpression of *DNT-3FL* also reduced Dcp1+ incidence throughout the medulla and lobula complex, as identified by N-cadherin counterstaining (Fig. S8A–C). To test whether DNT-2 could promote cell survival during optic lobe development, we overexpressed full-length *DNT-2* (i.e. *DNT-2FL*) or cleaved *DNT-2CK* in all neurons with *nsybGAL4.* They both reduced the incidence of Dcp1+ apoptosis in the lamina (Fig. 5I–J′,K) and medulla plus lobula complex (Fig. S8D–F), as identified by N-cadherin counterstaining. Altogether, overexpressed DNT-2/spz-5 and DNT-3/spz-3 reduced Dcp1+ apoptosis during optic lobe development.

To conclude, *DNT-3* (*spz-3*) and *DNT-2* (*spz-5*) are required to maintain cell survival and can decrease naturally occurring cell death during optic lobe development.

### DNT-2 promotes neuronal survival via Toll-2 in optic lobe development

To test whether the ability of DNTs to promote cell survival in the optic lobe depends on functioning through Toll receptors, we focused on DNT-2. DNT-2 binds Toll-6 preferentially and Toll-7 promiscuously (McIlroy et al., 2013). Both Toll-6 and Toll-2 are expressed in lamina neurons. Toll-2 is neuroprotective (Li et al., 2020), but its activating ligand is unknown. *DNT-2* is expressed in Mi1 medulla neurons (Figs 1D and 3A–C), which connect to L1 neurons (Nern et al., 2025) that express *Toll-2* (Figs 2D,H,L and 3D,E; Figs S1–S7). In horizontal views of the optic lobe, Toll-2>HisYFP can be clearly seen in lamina neurons, including Svp+ L1 lamina neurons (Fig. 6A). Homozygous null *DNT-2^37^/DNT-2^18^* mutants are viable (Sun et al., 2024; Ulian-Benitez et al., 2017; McIlroy et al., 2013). *Toll-2^pTV^GAL4* flies are heterozygous mutants for *Toll-2* and viable (Li et al., 2020). Remarkably, *DNT-2* homozygous mutant flies also bearing *Toll-2^pTV^GAL4* in heterozygosis were not fully viable, as only some escapers eclosed, revealing a functional interaction between these two genes. In fact, RNA interference (RNAi) knock-down of *Toll-2* in all neurons (i.e. *nybGAL4>Toll-2RNAi^GD36305^*) increased Dcp1+ apoptosis in the lamina (Fig. 6B,C), meaning that – like DNT-2 – Toll-2 is also required to maintain lamina neuron survival. Thus, using genetic epistasis analysis, we asked whether DNT-2 could maintain lamina neuron survival via Toll-2. When *DNT-2FL* was overexpressed concomitantly with *Toll-2RNAi^GD36305^* knock-down, Dcp1+ levels remained high (Fig. 6B,C). Interestingly, overexpression of *DNT-2FL* together with *Toll-2RNAi* knock-down increased the incidence of apoptosis beyond that caused by *Toll-2RNAi* knock-down alone (Fig. 6B,C). These data suggested that DNT-2 functions via Toll-2 to promote lamina neuron survival.

**Fig. 6.**
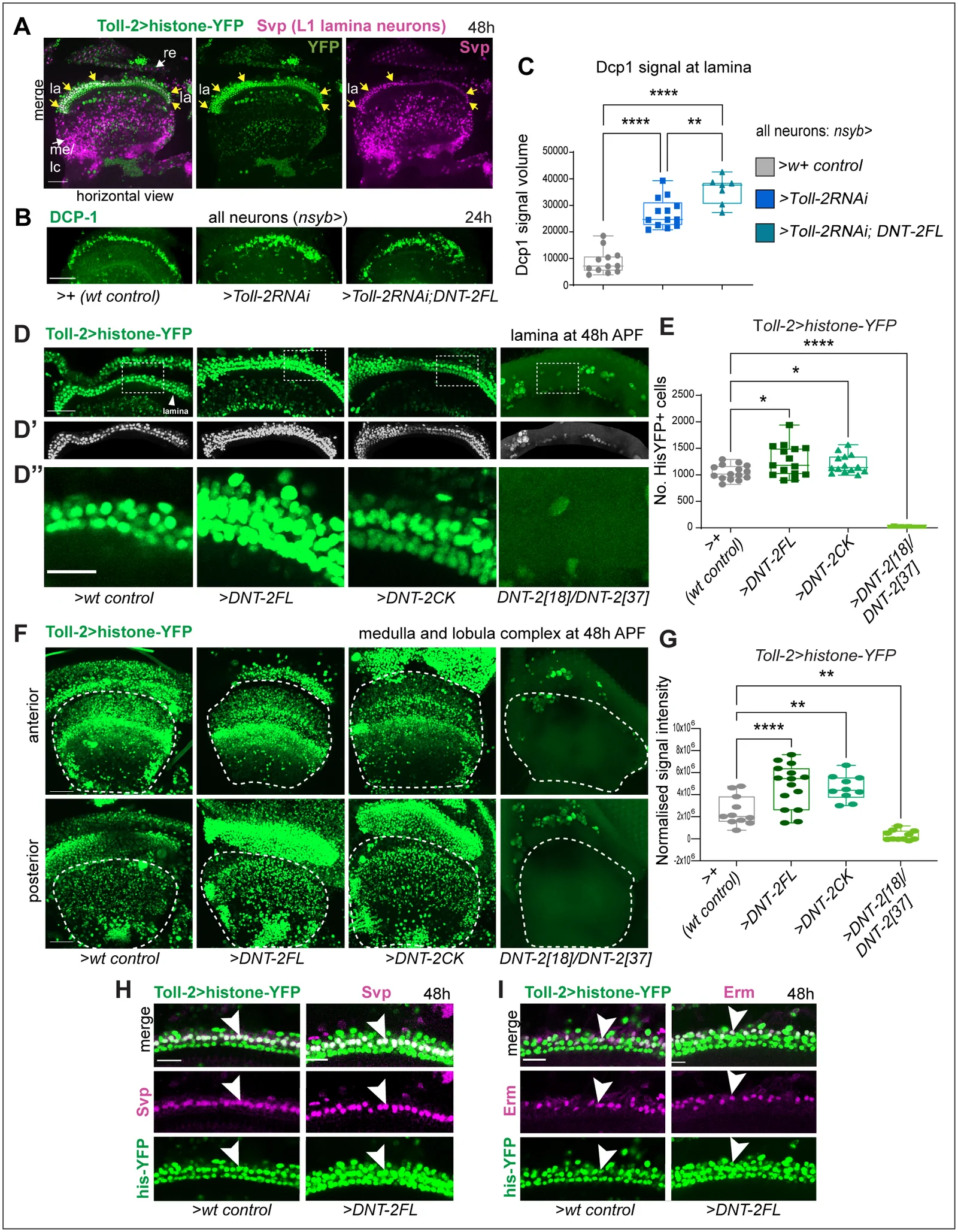
**DNT-2 functions with Toll-2 to regulate cell survival in the optic lobe.** (A) Toll-2>hisYFP+ Svp+ L1 lamina neurons (yellow arrows) seen in horizontal mounts of the optic lobe (48 h APF) (white arrows indicate retina, re, and medulla, me). (B,C) Pan-neuronal RNAi knock-down of *Toll-2* (*nsybGAL4>UAS-Toll-2^GD36305^-RNAi*) induced apoptosis in the lamina, and *DNT-2* overexpression had no effect when *Toll-2* was also knocked down (epistasis: *nsybGAL4>UAS-Toll-2^GD36305^-RNAi*, *DNT-2FL*) (24 h APF). One-way ANOVA ****P<0.0001 and Tukey's multiple-comparison test. (D–E) Overexpression of *DNT-2FL* or *DNT-2CK* in *Toll-2* cells increased lamina cell number (*Toll-2>his-YFP*, *DNT-2FL* and *Toll-2>his-YFP*, *DNT-2CK*), and most Toll-2> HisYFP+ lamina cells were lost in *DNT-2^18^/DNT-2^37^* mutants (48 h APF). Dotted ROIs indicate higher-magnification areas in D″. Automatic cell counting with DeadEasy Optic Lobe. Welch ANOVA ****P<0.0001, and Dunn's multiple-comparison correction. (F,G) Outside the lamina, overexpression of *DNT-2FL* or *DNT-2CK* in *Toll-2>hisYFP* cells increased signal intensity, and most *Toll-2>his-YTP+* cells and signals were lost in *DNT-2^18^/DNT-2^37^* mutants (48 h APF). One-Way ANOVA ****P<0.0001 and Dunnett's multiple-comparison correction. (F) Projections of anterior and posterior optic lobe sections to show effects throughout the optic lobe. Signal intensity was measured using Fiji and normalised to background signal. Dotted lines ROI indicate the optic lobe excluding lamina. (H,I) Excess Toll-2>hisYFP cells caused by *DNT-2FL* overexpression did not differentiate as Svp+ L1 or Erm+ L3 neurons (48 h APF). FL, full length; CK, cystine-knot domain. Arrowheads in H and I indicate L1 Svp+ neurons and Erm+ L3 neurons, respectively. Asterisks indicate multiple-comparison correction tests: *P<0.05, **P<0.01, ****P<0.0001. For further details, see Table S4. Scale bars: (A,B,D,D′,F) 50 µm; (D″,H,I) 20 µm.

Apoptosis can be verified by assessing whether alterations in cell death affect cell number. Toll-2+ lamina neurons are clearly distinct when labelled with the nuclear reporter histone-YFP (i.e. *Toll-2>hisYFP*) (Fig. 6A,D) and were quantified automatically using DeadEasy (Li et al., 2020). Thus, we asked whether altering DNT-2 levels could result in changes in Toll-2>hisYFP+ lamina cell number. We found that the reduction in apoptosis caused by DNT-2FL and DNT-2CK gain of function led to an increase in Toll-2>hisYFP+ lamina neuron number compared to wild-type controls (Fig. 6D,E). Conversely, the increase in apoptosis in *DNT-2^18^/DNT-2^37^* null mutants resulted in the loss of Toll-2>hisYFP+ lamina cells (Fig. 6D,E). The remaining cells were almost exclusively large, sporadically and erratically located HisYFP cells distributed mostly between the retina and lamina (Fig. 6D–D″). Conceivably, they could perhaps be macrophages having engulfed Toll-2>hisYFP dead cells, although we were unable to verify this. Finally, we asked whether altering DNT-2 levels might also affect Toll-2+ cell number outside the lamina, throughout the medulla and lobula complex. As too many cells were labelled with Toll-2>HisYFP, automatic cell counting was not possible, and we measured signal intensity instead. This showed that overexpression of either *DNT-2FL* or *DNT-2CK* increased Toll-2>HisYFP cell signal intensity in the medulla and lobula complex (Fig. 6F,G), consistent with an increase in cell number. Most dramatically, in *DNT-2^18^/DNT-2^37^* null mutants, most Toll-2+ cells throughout the optic lobe were missing, except for a few large, erratically distributed cells (Fig. 6F,G). These data demonstrate that the decrease in apoptosis caused by *DNT-2* gain of function increased the number of Toll-2+ cells, and the increase in apoptosis in *DNT-2* mutants caused Toll-2+ cell loss. This dramatic cell-loss phenotype could conceivably be the compound effect of a direct requirement for DNT-2 and Toll-2, plus the secondary effects of extreme cell loss. In any case, the dramatic loss of Toll-2>HisYFP cells in *DNT-2^18^/DNT-2^37^* homozygous mutants plus heterozygous *Toll-2^pTVGal4^* mutants strongly supports a requirement for DNT-2 functioning together with Toll-2 in maintaining neuronal survival.

Finally, we asked whether the excess Toll-2>hisYFP cells produced by DNT-2FL overexpression acquired a specific cell fate. Using the L1 lamina neuron marker anti-Svp, we found that overexpression of *DNT-2FL* did not alter the normal Svp profile (Fig. 6H). Similarly, *DNT-2FL* overexpression did not alter the L3 lamina neuron anti-Erm pattern either (Fig. 6I). These data show that upon *DNT-2FL* overexpression, excess neurons do not acquire L1 nor L3 cell fates. Perhaps they may acquire other lamina neuron fates or fail to follow a specific cell differentiation programme.

Together, these data show that DNT-2 functions with Toll-2 to maintain the survival of Toll-2+ neurons in the lamina, medulla and lobula complex during optic lobe development.

### DNT-2 and Toll-2 are required for appropriate neural circuit development

In the vertebrate nervous system, neurotrophic factors are produced in limiting amounts, and only neurons that receive them during targeting and connectivity (i.e. when presynaptic and postsynaptic neuronal partners meet, which is a requirement for synaptogenesis) are spared from naturally occurring cell death (Davies, 2003; Levi-Montalcini, 1987; Lu et al., 2005). In this way, cell number is adjusted to enable the emergence of functional neural circuits (Davies, 2003; Levi-Montalcini, 1987; Lu et al., 2005). In the *Drosophila* pupa, targeting, connectivity and synaptogenesis take place between 30 and 72 h APF (Millard and Pecot, 2018; Pecot et al., 2014; Hadjieconomou et al., 2011). Importantly, synaptogenesis as described by the expression of synaptic markers starts at 24 h and peaks at 60 h APF (Kurmangaliyev et al., 2020). Synapses can be visualised, and patterned stimulus-independent neuronal activity can be detected from 48 h APF and peaked at 72 h APF, meaning that at least some circuits are connected by these stages (Akin et al., 2019; Bajar et al., 2022). Thus, the period of naturally occurring cell death overlaps with that of neural circuit assembly and anticipates synaptogenesis. To ask whether DNT-2 with Toll-2 could function during neural circuit development, we first tested using retro-Tango (Sorkac et al., 2023) – which enables the unbiased identification of neuronal inputs – whether DNT-2 is required by lamina neurons for connecting to target medulla neurons. *DNT-2* is expressed in medulla neurons and putative retinal neurons (Fig. 1D,G,J–M). Expressing *Retrotango* in DNT-2 neurons labelled medulla neurons, which means that some DNT-2 neurons connect to other medulla neurons; and it also labelled lamina neurons (Fig. 7A). These data showed that lamina neurons are inputs of DNT-2 neurons. Furthermore, knocking down *DNT-2* expression with *DNT-2GAL4>UAS-DNT-2RNAi^VDRC41295^* decreased the expressivity of lamina input detection (i.e. there were fewer optic lobes with ten or more Retro-Tango-labelled lamina neurons than in controls, Fig. 7B,C). The incomplete penetrance of the phenotype could be explained by the incomplete knock-down with RNAi and redundancy with other molecular and cellular mechanisms underlying connectivity. In any case, these data show that DNT-2 is required for, or stabilises, connections between lamina and medulla neurons. *DNT-2* is also expressed in retinal cells; thus, Retro-Tango could reveal connections between DNT-2+ retinal neurons and lamina neurons as well. Either way, these data show that DNT-2 – which is not expressed in lamina neurons – is required for lamina neuron connections to form.

**Fig. 7.**
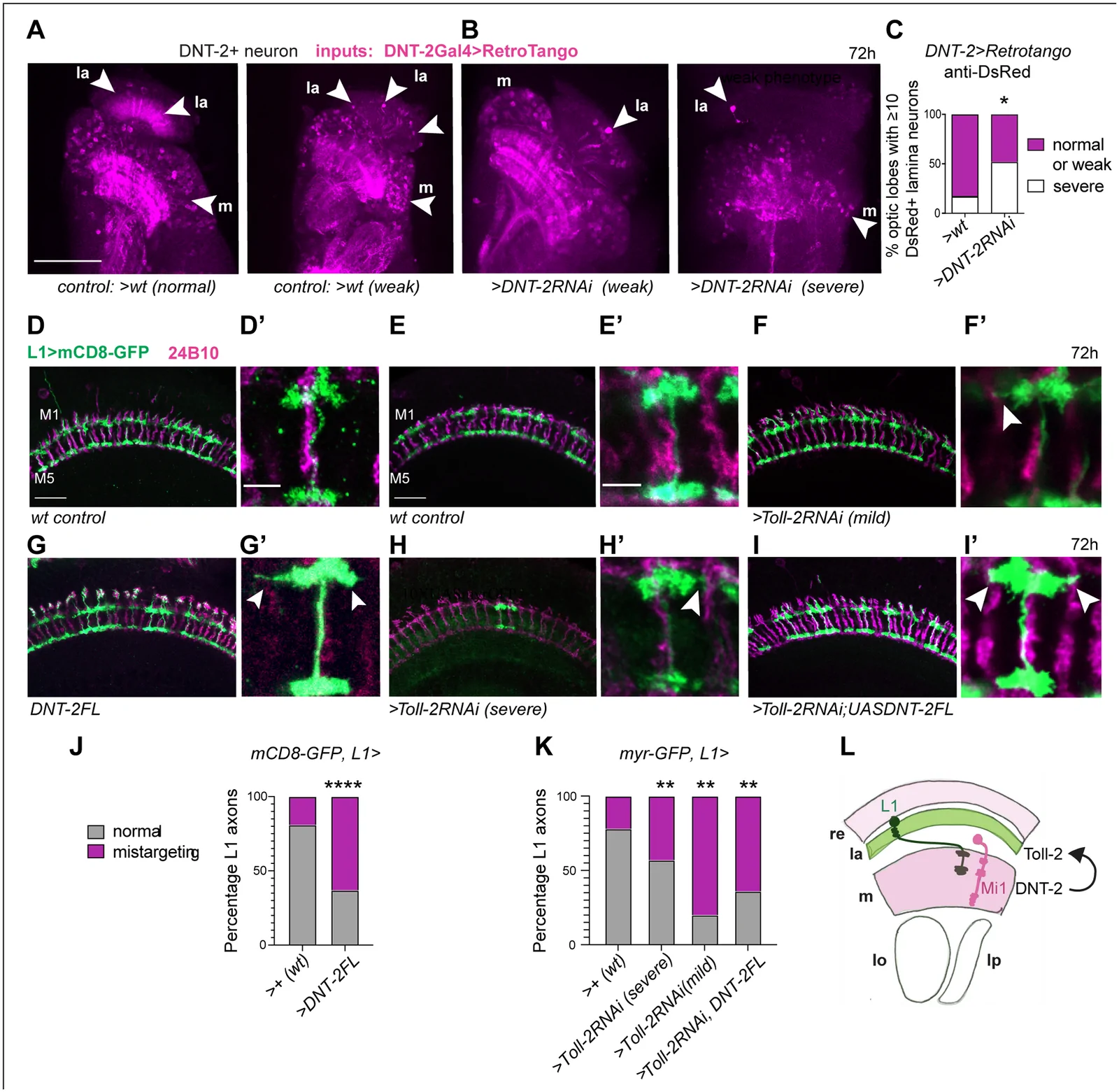
**Altering DNT-2 and Toll-2 levels modifies L1 connectivity and columnar organisation.** *DNT-2>Retrotango* revealed that lamina neurons are inputs of DNT-2 neurons (A). (B,C) *DNT-2>DNT-2*^V*DRC41295*−^*RNAi* knock-down reduced incidence of connecting lamina neurons, quantification in C is given as the percentage of optic lobes with normal (i.e. uncountable), weak (more than ten lamina neurons) or severely reduced (nine or fewer lamina neurons) connections to lamina neurons (72 h APF). Arrowheads indicate positions of lamina and medulla neuron somas. Chi-square: *P*=0.0273. (D,D′,G,G′,J) Overexpression of *DNT-2FL* in L1 lamina neurons (*L1 split>DNT-2FL*) caused axonal misrouting at the M1 layer (arrowheads); quantification in J. (E,E′,F,F′,H,H′,K) *L1 split>Toll-2^GD36305^-RNAi* knock-down in L1 lamina neurons caused a mild phenotype (50% penetrance, *n*=10) where neurons could misroute (F,F′,K, arrowheads) and a severe phenotype in which most L1 neurons were missing (50% penetrance, *n*=10) and the remaining neurons misrouted (arrowheads) (H,H′,K). (I,I′,K) *Toll-2^GD36305^-RNAi* knock-down together with DNT-2 overexpression (*L1 split>Toll-2^GD36305^-RNAi, DNT-2FL*) also caused misrouting. Quantification in K: Fisher's exact test **P<0.01 and *post hoc* Bonferroni multiple comparisons corrections. (L) Diagram illustrating that L1 lamina neurons expressing Toll-2 can receive DNT-2 secreted from the medulla or retina to influence columnar organisation. FL, full length. Asterisks refer to multiple-comparison corrections: *P<0.05, **P<0.01, ****P<0.0001. (D′–I′) Higher-magnification views of D–I. Stage: all 72 h APF. For further details, see Table S4. Scale bars: (A,B) 50 µm (D–I) 20 µm; (D′–I′) 5 µm.

As *Toll-2* is expressed in L1 and L3 lamina neurons (Fig. 2D,H,L), to visualise L1 neurons, we used L1-specific split-GAL4 (R48A08AD; R66A01DBD) (Tuthill et al., 2013) to drive the expression of *mCD8-GFP* or *myr-GFP* reporters and tested the effects of altering gene expression on L1 axonal and dendritic patterns. L1 neurons normally project along columns that can be labelled with the photoreceptor marker mAb24B10 (providing a spatial reference to identify medulla columns) and target layers M1 and M5 of the medulla. The M1 layer is particularly relevant as, here, L1 neurons (which express *Toll-2*) connect with Mi1 medulla neurons (which express *DNT-2*). When we overexpressed *DNT-2* in L1 neurons (i.e. *L1-splitGAL4>mCD8GFP* and *DNT-2FL*), L1 axonal terminals in the M1 layer could cross over to neighbouring 24B10+ columns (Fig. 7D,D′,G,G′,J). To test whether Toll-2 was required for appropriate columnar organisation of L1 neurons, we knocked down *Toll-2* using RNAi (i.e. *L1-split-GAL4>myrGFP* and *Toll-2RNAi^GD36305^*), and this produced both mild and severe phenotypes (50% penetrance for each, *n*=10 brains). In severely affected optic lobes, most L1 neurons were missing, consistent with the increase in cell death in this genotype, and surviving neurons could misroute at the M1 layer to nearby columns (Fig. 7E,E′,H,H′,K). In mildly affected optic lobes, more neurons remained, and L1 axonal terminals could misroute (Fig. 7F,F′,K). Interestingly, *Toll-2RNAi* knock-down did not alter the phenotype caused by *DNT-2FL* overexpression, and fasciculation was impaired to the same extent as with each genetic manipulation alone (Fig. 7I,I′,K). These data showed that both the excess and deficiency of DNT-2, as well as loss of Toll-2, impaired axonal columnar organisation.

These data show that DNT-2 and Toll-2 are required for appropriate connections between L1 neurons and medulla neurons to form and for appropriate columnar organisation at the M1 medulla layer.

### DNT-2 regulates lamina neuron dendritic complexity via Toll-2

Lamina neurons have a distinctive dendritic morphology, including mushroom-shaped dendritic spines, and are known to be plastic (Weber et al., 2009). To investigate whether DNT-2 and Toll-2 may affect L1 neuron dendritic morphology, we used the *L1-split-GAL4* driver line (R48A08AD; R66A01DBD) (Tuthill et al., 2013), as above; visualised L1 dendrites with *UASmCD8-GFP* or UAS-myrGFP; and traced the dendritic shape and spines with Amira in 3D. We overexpressed *DNT-2FL* (i.e. *L1-splitGAL4>mCD8GFP* and *DNT-2FL*) in L1 neurons, and this resulted in a significant increase in dendritic volume compared to controls and larger and more abundant dendritic spines (Fig. 8A,C). To investigate whether Toll-2 was involved, we knocked down *Toll-2* expression with RNAi (i.e. *L1-split-GAL4>myrGFP* and *Toll-2RNAi^GD36305^*). As before, this resulted in some optic lobes with very few L1 neurons, as most of them were missing (‘severe’ phenotype, penetrance 60%, *n*=5 brains), and other optic lobes with multiple L1 neurons (‘mild’, penetrance 40%, *n*=5 brains). In both categories, *Toll-2* RNAi knock-down resulted in a decrease in overall dendrite size and a marked decrease in dendritic spines (Fig. 8B,D). This shows that Toll-2 is required for maintaining both size and spine architecture of L1 dendrites. Finally, overexpressed *DNT-2FL* together with knocking down *Toll-2* (i.e. *L1-split-GAL4>myrGFP*, *Toll-2RNAi^GD36305^*, and *DNT-2FL*) did not alter the phenotype of *Toll-2-RNAi* knock-down (Fig. 8B,D). Altogether, these data showed that *DNT-2* overexpression increased and *Toll-2* loss of function decreased dendrite size and complexity and that *DNT-2* overexpression had no effect when Toll-2 was missing (i.e. *Toll-2* is epistatic to *DNT-2*). These data show that DNT-2 and Toll-2 regulate L1 dendritic morphology, and they support that DNT-2 functions with Toll-2.

**Fig. 8.**
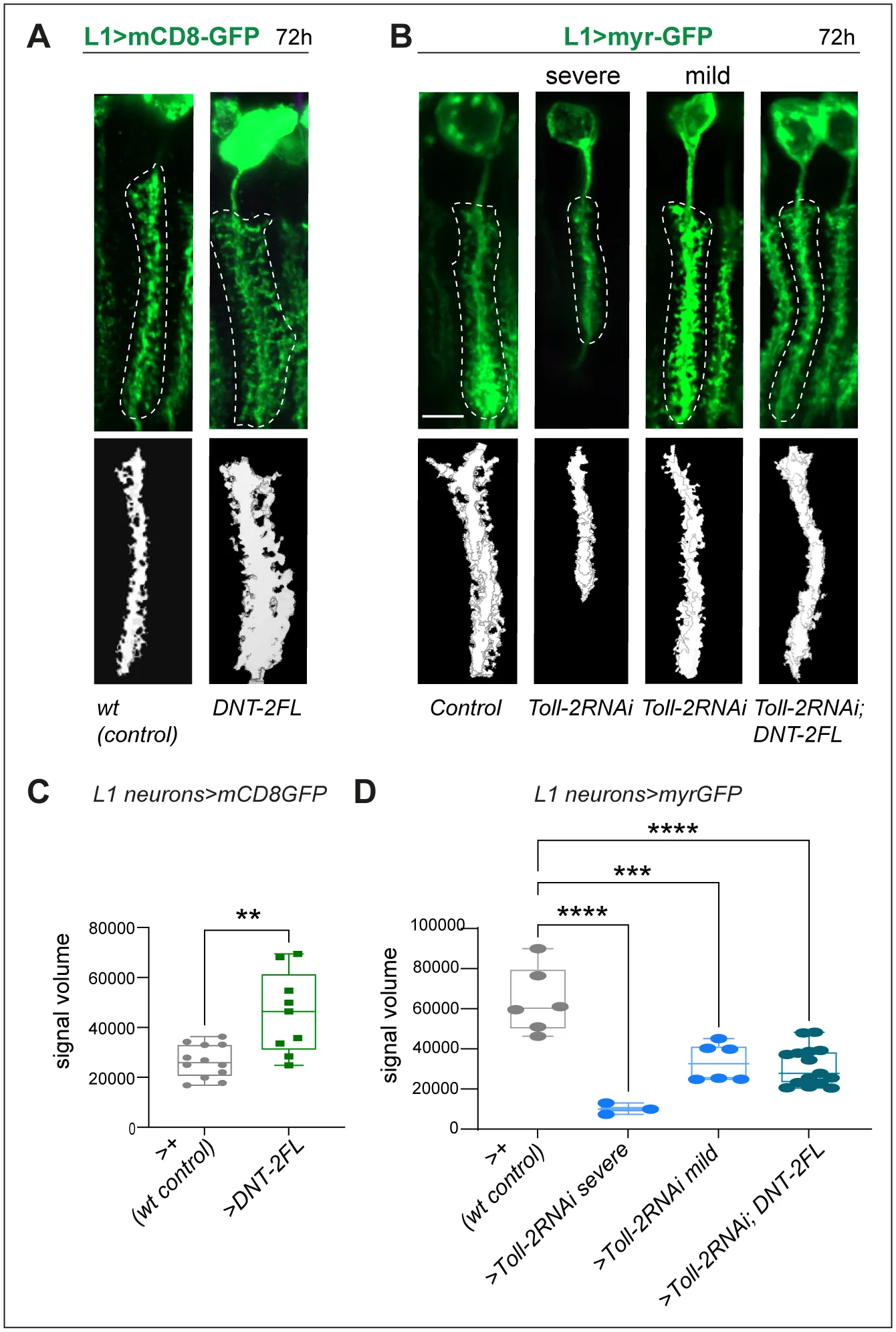
**DNT-2 with Toll-2 regulates L1 dendrite size.** (A) Overexpression of *DNT-2FL* in L1 neurons increased dendrite size (*L1 split>mCD8-GFP, DNT-2FL*); quantification of dendrite volume in C using Amira. Student’s *t*-test *P*<0.01. (B) *Toll-2^GD36305^-RNAi* knock-down decreased dendrite size in severely (40% penetrance, *n*=5) and mildly (60% penetrance, *n*=5) affected specimens (*L1 split>myrGFP*, *Toll-2^GD36305^-RNAi*). *DNT-2FL* overexpression did not modify the phenotype of *Toll-2RNAi^GD3630^* knock-down (*L1 split>myrGFP*, *Toll-2^GD36305^-RNAi*, *DNT-2FL*); quantification of dendrite volume (D): Kruskal–Wallis ANOVA **P<0.01 and Dunn's multiple-comparison correction. (A,B) Projections of raw data images with a dotted line around the dendrites. (A′,B′) Amira 3D rendering of dendrite morphology. Stages: all 72 h APF. FL, full length. Asterisks in D refer to multiple-comparison corrections: **P<0.01 ***P<0.001, ****P<0.0001. For further details, see Table S4. Scale bar: 5 µm.

## DISCUSSION

Neural circuit assembly requires the precise coordination of cell survival, targeting and synaptogenesis. In the *Drosophila* visual system, this process is remarkably stereotypic: 750–800 ommatidia form precise connections across lamina cartridges and medulla columns and layers, preserving retinotopy and enabling appropriate vision. The robustness of circuit development reflects the combined action of genetic cascades controlling stem cell proliferation, guidance molecules directing axonal navigation and glial migration, cell–cell interactions driving axonal reassortment, and molecular matching between synaptic partners (Agi et al., 2024; Courgeon and Desplan, 2019a; Holguera and Desplan, 2018; Malin et al., 2024; Pecot et al., 2014; Melnattur and Lee, 2011; Millard and Pecot, 2018). Concurrent with these events, a wave of naturally occurring cell death sweeps through the pupal optic lobe (Hara et al., 2013, 2018; Togane et al., 2012). Such death likely reflects metamorphic remodelling, elimination of aberrant cells, and adjustment of interacting populations during circuit formation (Hara et al., 2013; Pinto-Teixeira et al., 2016; Togane et al., 2012; Pecot et al., 2014). In fact, throughout development, between 50% (e.g. in *Drosophila*) and 80% (e.g. in vertebrates) of neurons are lost to naturally occurring cell death, and the control of survival during this period enables adjustment of neuron numbers, facilitating neural circuit establishment (Rogulja-Ortmann et al., 2007; Pinto-Teixeira et al., 2016; Davies, 2003; Dekkers et al., 2013; Raff et al., 1993). Our findings demonstrate that *DNT-2* (*spz-5*) and *DNT-3* (*spz-3*) have evolutionarily conserved neurotrophin functions during this critical period of *Drosophila* visual system development.

Both *DNT-2/spz-5* and *DNT-3/spz-3* are expressed in the retina and medulla and are required for, and can promote, the survival of lamina and medulla neurons. Focusing on DNT-2 and Toll-2, we identified medulla neurons expressing DNT-2 as Bsh+ Mi1 neurons and Toll-2 lamina neurons as Svp+ L1 and Erm+ L3 neurons. Furthermore, GRASP and Retro-Tango data confirmed that DNT-2 and Toll-2 neurons connect with or are proximal to each other, consistent with connectome data (Nern et al., 2025). These data suggest that DNT-2 is produced by Mi1 medulla neurons and retinal cells and maintains non-autonomously the survival of L1 lamina neurons expressing Toll-2.

In fact, we have shown that both *DNT-3* and *DNT-2* can rescue naturally occurring cell death throughout the optic lobe; that *DNT-3*, *DNT-2* and *Toll-2* are required for lamina neuron survival; that *DNT-2* functions via *Toll-2* to promote lamina neuron survival; and that loss of function for *DNT-2* in a heterozygous *Toll-2* mutant background caused loss of Toll-2+ neurons throughout the optic lobe. It has been proposed that dying cells release DNT-2, which is received by Toll-6 in glial cells, triggering phagocytosis of dead cells (McLaughlin et al., 2019). Accordingly, an increase in Dcp1 levels in *DNT-2* mutants could be due to reduced phagocytosis rather than increased cell death. However, our combined loss and gain of function and epistasis data support a more direct interpretation that, in the absence of DNT-2, Toll-2+ neurons die. Our findings are consistent with the finding that *DNT-3* (*spz-3*) promotes cell survival in the adult central brain (Coutinho-Budd et al., 2017) and that *DNT-2* promotes cell survival in the embryonic CNS and adult brain and its receptor *Toll-6* in the embryonic, larval and pupal ventral nerve cords and adult brains, and Toll-2 in the pupal and adult central brain Li et al., 2020 (Foldi et al., 2017; Sun et al., 2024; Zhu et al., 2008; McIlroy et al., 2013). Interestingly, when *Toll-2* was knocked down, overexpression of *DNT-2* increased apoptosis relative to *Toll-2RNAi* alone (Fig. 6C). This could be explained because Toll-6 – a DNT-2 receptor (McIlroy et al., 2013) – can promote either cell survival or cell death depending on the cell context, inducing apoptosis in the pupal CNS (Foldi et al., 2017). Thus, in the absence of Toll-2, DNT-2 overexpression could drive cell death via Toll-6. In summary, our data show that during the peak of apoptosis in pupal optic lobe development, DNT-2 and DNT-3 are required for and can promote lamina neuron survival and can promote the survival of medulla and lobula complex neurons.

DNTs/Spzs can function naturally as full-length or cleaved, mature ligands (Delotto and Delotto, 1998; Hu et al., 2004; Weber et al., 2003; Coutinho-Budd et al., 2017; Foldi et al., 2017; McIlroy et al., 2013). Importantly, overexpressed mature forms of Spz-1, DNT-1 and DNT-2 are functional *in vivo*, most likely acting cell-autonomously (Hu et al., 2004; Ligoxygakis et al., 2002; Sun et al., 2024; Ulian-Benitez et al., 2017; Zhu et al., 2008). Overexpressed full-length DNT-2 and DNT-3 are naturally cleaved and secreted, enabling non-cell-autonomous receptor activation (Coutinho-Budd et al., 2017; Sun et al., 2024; Ulian-Benitez et al., 2017; Zhu et al., 2008; McIlroy et al., 2013). Consistent with this, DNT-3FL, but not DNT-3CK, reduced apoptosis when expressed in Toll-8+ neurons, likely because only DNT-3FL is secreted and can act at a distance.

Genetic interaction data strongly suggest that DNT-2 is a candidate ligand for Toll-2: *Toll-2* is expressed in L1 neurons connecting to Mi1 neurons that express *DNT-2*; loss of either gene function increased Dcp1+ apoptosis in the lamina; combined heterozygous *Toll-2* and homozygous *DNT-2* mutations caused semi-lethality and loss of most Toll-2+ neurons; DNT-2 excess increased Toll-2+ neuron number; and *Toll-2* knock-down prevented both the pro-survival and dendritic growth effects of *DNT-2* overexpression. Genetic interaction analysis is commonly used to order genes into signalling pathways and identify ligand–receptor pairs (Ballard et al., 2014; Grosshans et al., 1994; Hecht and Anderson, 1993). With Toll-2, our data expand the receptor repertoire for DNT-2, which was previously shown to bind Toll-6 and Kek-6 and to bind Toll-7 and Kek-2 promiscuously (McIlroy et al., 2013; Ulian-Benitez et al., 2017; Sun et al., 2024; Foldi et al., 2017).

Our data suggest that survival of different lamina neurons might depend on different Tolls or combinations of Tolls and their ligands, whilst the promiscuity of DNT/Spz–Toll interactions means that each ligand could function via multiple receptors in different cell types. L1 neurons express *Toll-2*; L2, *Toll-6*; L3, *Toll-2* and *Toll-6*; and L4, *Toll-6* and *Toll-8*. Since Toll-2 and Toll-6 are both receptors for DNT-2 and Toll-8 is the proposed receptor for DNT-3/Spz-3, different lamina neurons could be regulated by different ligands, Tolls or a combination of Tolls. The finding that loss of either *DNT-2* or *DNT-3* alone is sufficient to cause lamina cell death shows that these ligands do not naturally compensate for each other's absence. This could be due to ligand production in limiting amounts, origin from distinct cellular sources, or preferential receptor binding. Either way, it suggests the intriguing possibility that a combinatorial code of DNT/Spz ligands and Toll receptors could regulate survival of distinct neuronal types in the visual system. In the future, it will be compelling to test the functions of *spz-1*, *spz-4*, *spz-2* (*DNT-1*) and *spz-6* and their Toll receptors in visual system development.

The neurotrophic functions identified here are not unprecedented, as other survival signals also operate during *Drosophila* visual circuit formation: the ligand Jeb is produced by R1–R6 photoreceptor axons to activate the receptor Alk located in L3 lamina neuron dendrites and control their survival (Pecot et al., 2014; Togane et al., 2012); L5 neurons are produced in excess, but only the one that receives Col4A1 and EGF signalling activates MAPK, remains alive and differentiates into L5 neurons (Fernandes et al., 2017; Prasad et al., 2022); and DIP–Dpr interactions regulate the survival of Dm8, Dm12 and Dm14 medulla neurons (Courgeon and Desplan, 2019b; Xu et al., 2022, 2018). Cell number is also regulated through cell proliferation and spatial patterning by Dpp/BMP signalling during neurogenesis in larvae (Malin et al., 2024). These mechanisms are complementary: proliferation and spatial patterning act during neurogenesis, whilst apoptosis and survival regulation refine cell number during pupal development, as neural circuits are assembled.

Interference with the normal levels of DNT-2 and/or Toll-2 also impaired connections between DNT-2 and L1 neurons, L1 axonal columnar organisation at the M1 medulla layer and dendritic morphology. Our Retro-Tango data revealed a requirement for DNT-2 in the formation or stabilisation of contacts between lamina and medulla neurons, whilst the incomplete phenotypic penetrance meant that DNT-2 functions within a context where multiple molecular and cellular cues control the formation of neural circuit establishment. On the other hand, both *DNT-2* overexpression and *Toll-2* knock-down caused L1 axonal terminals to cross into neighbouring columns. Both up- and down-regulation of *DNT-1* and *DNT-2* levels caused the same phenotype in embryonic axon targeting too (Zhu et al., 2008), which was explained as neurotrophic factors being released in limited amounts and both up- or down-regulation disrupting the information carried by secreted ligand gradients (Davies, 2003). In any case, there is no evidence that the ectopic axonal projections result in functional connections. Altered targeting with *Toll-2RNAi* could equally reflect the indirect consequences of loss of neighbouring cells through cell death. In fact, the phenotype is reminiscent of that caused by altered Dscam2 or Fez levels (Millard et al., 2007; Peng et al., 2018), suggesting that impaired contact-mediated repulsion might be involved. Loss of contact repulsion – or other indirect effects – could explain axonal misroutings in *Toll-2* knock-down specimens, where neurons are lost. However, impaired contact-mediated repulsion could not explain *DNT-2* overexpression data where neighbouring cells are not missing but in excess. Thus, the most parsimonious interpretation is that appropriate DNT-2 and Toll-2 levels and the signalling events they induce are instructive for columnar specificity. By contrast, the dendritic phenotype suggested a direct involvement of DNT-2 and Toll-2 in dendritic development, like in adults (Li et al., 2020; Sun et al., 2024). The DNT-2/Toll-2 axonal and dendritic phenotypes could reflect coupling of cell survival control with neural circuit development, as envisioned by the neurotrophic theory, whereby neurotrophins maintain neuronal survival and promote neurite growth and synaptogenesis in connecting neurons (Lu et al., 2005; Park and Poo, 2013; Levi-Montalcini, 1987). Alternatively, DNT-2 and Toll-2 could have pleiotropic functions regulating axonal branching and dendritic morphology at different stages and independent from regulating cell survival. In fact, DNT-2, Toll-2 and Toll-6 positively regulate neurite growth and synaptogenesis in the larval neuro muscular junction (NMJ) and the adult brain (Li et al., 2020; Sun et al., 2024; Ulian-Benitez et al., 2017; Sutcliffe et al., 2013), similarly for mammalian neurotrophins (Lu et al., 2005; Park and Poo, 2013). Our findings do not have sufficient resolution to discriminate between these possibilities, but they do show that evolutionarily conserved neurotrophins regulate neuronal survival and axonal and dendritic growth in the developing visual system of *Drosophila*.

Neurotrophins are required to maintain neuronal survival and enable connectivity in the vertebrate visual system. For example, BDNF, with its receptor TrkB, promotes neuronal survival (Cohen-Cory and Fraser, 1994), as well as axonal arborisation and synapse maturation in retinal ganglion cells during retinotectal synaptic connectivity (Marshak et al., 2007; Sanchez et al., 2006). Disrupting TrkB signalling altered the branching and synaptic maturation of presynaptic axon arbors and suggested that presynaptic TrkB signalling in retinal ganglion cells is a key determinant in the establishment of visual connectivity (Marshak et al., 2007). In *Drosophila* optic lobes, naturally occurring cell death overlaps with targeting and synaptogenesis, it anticipates the onset of patterned spontaneous activity (Akin et al., 2019; Bajar et al., 2022), and DNTs and Tolls are expressed throughout these events, meaning that neurotrophic signalling is concurrent with circuit assembly. Our findings that interfering with DNT-2 and Toll-2 levels affected cell number and axonal and dendritic patterns support a deep evolutionary strategy adjusting cell number during circuit assembly in brain development.

To conclude, our findings show that DNT-2, produced by Mi1 medulla neurons, acts through the Toll-2 receptor in L1 lamina neurons to maintain their survival, enable appropriate axonal columnar organisation and regulate dendritic complexity. DNT-2 might also influence neurons expressing its well-known receptor, Toll-6. DNT-3 similarly promotes neuronal survival in the optic lobe, perhaps together with Toll-8. In this way, the survival of different visual system neurons expressing different Tolls or combinations of Tolls could be regulated by distinct DNT/Spz ligands. The regulation of cell survival by DNTs and Toll receptors provides an additional strategy contributing to the robustness of neural circuit development and further supports neurotrophism as a fundamental evolutionarily conserved principle of nervous system development.

## MATERIALS AND METHODS

### Genetics

Please see Table S1 for the list of stocks used and Table S4 for the full genotypes in each experiment. Mutants: *DNT-2^37^* and *DNT-2^18^* are protein null alleles (Foldi et al., 2017; Ulian-Benitez et al., 2017; Sun et al., 2024). *spz3^46^* and *spz3^27^* are loss-of-function alleles, generated from P-element excision from *spz-3^EY06670^.* GAL4 driver lines: In-frame T2A fusions generate a shared transcript between the gene of interest and GAL4 that liberates a functional GAL4 protein that reproduces the endogenous expression of the gene of interest. Spliced-in fusion transcript was verified by PCR. Therefore, these drivers reproduce the endogenous expression of the gene of interest. *spz-1^MIO2318^-T2A-Gal4* was generated by recombinase-mediated cassette exchange (RMCE) by inserting T2A-Gal4 in frame into the MIMIC allele *spz-1^MIO2318^*. *spz-3/DNT-3-T2A-GAL4* was generated by CRISPR/enhanced homologous recombination to insert T2A-Gal4 in frame within the first intron of the gene (see below). *Spz-4-T2A-GAL4* was generated by RMCE, by inserting T2A-Gal4 in frame into intronic *spz-4^MI15678^* MIMIC allele. *DNT-2-T2A-Gal4* is a CRISPR/Cas9-knock-in allele, with GAL4 at the start of the gene (Sun et al., 2024). *Toll-1-T2A-Gal4* is a CRISPR/Cas9 knock-in at the start of the gene (Singh et al., 2025). *Toll-2^PTV^GAL4* (Li et al., 2020) has a fully functional GAL4 coding region inserted into the attP site of the pTV cassette (Baena-Lopez et al., 2013) that replaces the coding region for Toll-2 (Li et al., 2020). Toll-6GAL4^MIO2127^ was generated by RMCE by inserting GAL4 into the *Toll-6^MIO2127^* MIMIC in the only exon of this intron-less locus (Li et al., 2020). *Toll-8GAL4^MD806^* is a P-element insert-180 bp upstream of the Toll-8 start codon (Li et al., 2020). *Nsyb-Gal4* drives GAL4 in all neurons (BDSC #3917). L1-split GAL4: *w*;*84A08-p65ADZp attp40*; *66A01-ZpGdbd attp2* (gifted by the Reiser Lab, Janelia Research Campus, USA) (Tuthill et al., 2013). Reporter lines: *UAS-mCD8::GFP*, for membrane-tethered GFP; *UAS-histone-YFP*, for YFP-tagged nuclear histone; *10XUAS-myr::GFPattP40* on the second chromosome (BDSC #32198), *10XUAS-myr::GFPattP2* on the third chromosome (BDSC #32197), and 10XUAS-myr::GFP, su(Hw) attP8 on the X-chromosome (BDSC #32196) whereby a myristoylation tail at the N-terminus of GFP associates it to the plasma membrane; 20XUAS-6XmCherry-HA attp2 on the third chromosome (from BDSC #52268) for cytoplasmic expression of a hexameric form of mCherry. UAS for gene overexpression, knock-down and epistasis: *UAS-DNT-2FL* (full length) and *UAS-DNT-2CK* (signal peptide plus cystine knot, mature form) (Foldi et al., 2017; Sun et al., 2024; Ulian-Benitez et al., 2017; Zhu, 2013); *UAS-spz3FL* (full length) and UAS-spz3CK (signal peptide plus cystine knot, mature form) (this work); *UAS Toll-2 RNAi^v36305^* (VDRC36305) (Li et al., 2020); *UAS-DNT-2RNAi^VDRC41295^* (VDRC); GRASP (BDSC #64315); retro-Tango (BDSC #99661). To select pupae of the desired genotype, the fusion balancer SM6aTM6B, marked with Tb− and which segregates the second and third chromosomes together, was used. All experiments were carried out at 25°C unless otherwise indicated.

#### MCFO clones

*hs-FLPG5.PEST;;10xUAS (FRT. stop) myr::smGdP-OLLAS 10xUAS (FRT. stop) myr::smGdP-HA 10xUAS (FRT. stop) myr::smGdP-V5-THS-10xUAS (FRT. stop) myr::smGdP-FLAG* (BSC64086) flies were crossed to *w;Toll-8GAL4*, *w;Toll-6^MIO2127^GAL4* or *w;Toll-2^pTV^GAL4* flies and bred at 25°C. L3 wandering larvae were watched until pupariation; 20 white pupae were fished out at once and moved to a new vial, placed at 25°C for 24 h (24 h APF), heat shocked in a water bath at 37°C for 5 min, then placed at 25°C until they reached 72 h APF, and then dissected, fixed and stained.

### Molecular biology

*Spz-3/DNT-3-T2A-GAL4* was generated by CRISPR/enhanced homologous recombination, by inserting T2A-GAL4 from plasmid pT-GEM(1) phase 1 (Addgene 62893) into the first intron of the *spz-3/DNT-3* coding region (between the first and second coding exons) to create a spliced in-frame fusion transcript with the 5′-terminus of *spz-3/DNT-3* mRNA and bearing T2A-GAL4 mRNA in frame. The guide RNA (gRNA) targeted the same intron (gRNA primers: GTCGTTTGGGTCGCTCGATGTCT and AAACAGACATCGAGCGACCCAAAC, Table S2), and it was designed using the Optimal Target Finder. *Bbs*I enzyme sites were added to the oligos used to generate the gRNA. The gRNA was cloned into pU6.3 using conventional ligation. 1 kb of genomic DNA was amplified for each homology arm, from nos-Cas9 genomic DNA using a Q5 enzyme (Promega), using the following primers: 5′ homology arm, GGTATACCGGTCGAATAAGTGACTCAAGCAGAC and ATAGCGGCCGCAGTATCTGAGTTTTGGTCTTG; 3′ homology arm, TATGGTACCTCTTGGCGCGGCACTCAAGT and CACACTAGTCTCATGCCGGCGAACCTATC. To clone the homology arms into the pT-GEM(1) plasmid, *Age*I and *Not*I cut sites were added at the extreme of the 5′ homology arm, and *Kpn*I and *Spe*I cut sites were added to 3′ homology arm ends. Both constructs were injected into flies expressing Cas9. After the selection of transformants carrying the 3xP3-RFP marker, stocks were balanced, and the red fluorescent marker was removed by Cre recombinase. Cleaved UAS-spz-3CK/DNT-3CK was made by cloning the signal peptide of *spz-5* (100 bp) inserted between *Eco*RI and *Bgl*II, followed by the cystine-knot domain of *spz-*3 (primers: spz-5 SP, CGGAATTCATGCAAATCGACGGCGAATGA with the *Eco*RI site and GAAGATCTCGAGCTGTGGGCGGCTACTGT with *Bgl*II; start of spz-3 cystine knot, GAAGATCTGCCGGAGGAAGTCGAAATAGA with the *Bgl*II site and end CCGCTCGAGCAGAGTCAGGTAATCTAGGGA with *Xho*I), into pUAS-attB, and Phi-C31 transgenesis into 86Fa. Full-length UAS-spz-3FL/DNT-3-FL was generated by Gateway cloning from cDNA full-length clone RE22741. With primers GGGGACAAGTTTGTACAAAAAAGCAGGCTCGCTAGCATATTTCGCACGCCC and GGGGACCACTTTGTACAAGAAAGCTGGGTCGGGATTACATCTACAGACAC (Table S2), the coding sequence of spz-3/DNT-3 was amplified, and the PCR product was inserted with a BP reaction into attB sites of the pDONR221 vector to generate an entry clone with attL sites. Next, *spz-3/DNT-3-FL* was inserted into attR sites of the destination vector *pUAS-GW-attB* with an LR reaction to generate the final expression clone with attB sites, and the final construct was inserted with Phi-C31 transgenesis into the attP2 landing site. 3xP3-RFP was removed with Cre recombinase.

#### Analysis of scRNA-seq from published databases

We analysed scRNA-seq data from the study by Kurmangaliyev et al. (2020) (GSE156455), specifically the preprocessed dataset covering 24–96 h APF, accessed via Zenodo (https://doi.org/10.5281/zenodo.4264808). All downstream analyses were performed using Seurat v3 in R. The data were imported as a Seurat object, and cells corresponding to specific timepoints (e.g. 24 and 36 h) were subsetted based on the provided metadata. Dimensionality reduction was carried out using principal component analysis, followed by Uniform Manifold Approximation and Projection (UMAP) embedding computed on the first 30 principal components. Cluster annotations provided by the original authors were used for all cluster-level analyses and visualisations. For feature visualisation, FeaturePlot and custom ggplot2-based UMAP plots were generated, incorporating manually defined colour palettes and cluster-specific labelling where appropriate.

### Immunostaining

Immunostaining of pupal brains was carried out following standard protocols. Pupae were staged by picking the white pupa at the end of L3 wandering. For each stage (24, 48, 72 h APF), the date and time of fishing were recorded to start dissection at the appropriate time point. Dissections were carried out in PBS with forceps in 20-min windows. Dissected brains were placed in 4% formaldehyde and kept on ice: for Dcp1, brains were fixed for 20 min; for all other staining procedures, they were fixed for 35 min. After fixation, brains were blocked for 2 h in 10% normal goat serum, and primary antibodies were incubated overnight. This was followed by incubation in secondary antibodies and washes. Primary antibodies used were as follows: mouse anti-24B10 (DSHB, Iowa) at 1:250 dilution; rabbit anti-GFP (Thermo Fisher Scientific) at 1:250 for normal stainings; mouse GRASP-specific anti-GFP (Sigma/Merck G6539) at 1:200; rabbit anti-Dcp1 (Cell Signalling) at 1:250; rat anti-N-cadherin (MAb DN-Ex) at 1:250; chicken anti-HA (Aves) at 1:100; rabbit anti-DsRed (Clontech, 632496) at 1:100; mouse anti-Svp (DSHB, Iowa) at 1:10; rabbit anti-Erm (a kind gift from Uwe Walldorf) at 1:100; guinea pig anti-Bsh (a kind gift from Larry Zipursky) at 1:500; and mouse anti-mCherry (DSHB, Iowa) at 1:50. Secondary antibodies used were as follows: Alexa Fluor 488 donkey anti-rabbit at 1:250; Alexa Fluor 647 goat anti-mouse at 1:250; Alexa Fluor 647 goat anti-rat at 1:250; Alexa Fluor 647 goat anti-chicken at 1:250; Alexa Fluor 488 goat anti-guinea pig at 1:250; Alexa Fluor 647 donkey anti-rabbit at 1:250; and Alexa Fluor 594 goat anti-mouse at 1:250. Details of primary and secondary antibodies used and their origin and working dilutions are also provided in Table S3.

### Microscopy and imaging

#### Mounting of optic lobes

For visualisation of expression profiles (Figs 1 and 2), optic lobes were mounted vertically; for all other data, they were mounted horizontally.

Laser scanning confocal microscopy was carried out using Zeiss (LSM710) (Figs 1 and 2) at a resolution of 512×512 or 1024×1024 pixels, speed 6 or 7, 20× objective and step 1 µm. For DCP-1 staining in Figs 5 and 6A,B and Fig. S8, samples were scanned using Zeiss LSM900 with Airyscan 2, at 512×512 pixels, using a 25× oil objective with 0.9× zoom, speed 7 and step 1 µm. For histone-YFP in Fig. 6C–F, Zeiss LSM900 was used at 1024×1024 pixels, 25× oil objective 0.8× zoom, speed 7 and step 1 µm. Figs 7 and 8 data were acquired with Zeiss LSM900 at 1024×1024, no zoom and 2× averaging for axons (Fig. 7) and 2× zoom and 8× averaging for dendrites (Fig. 8), step 1 µm.

### Quantification of apoptosis

Anti-Dcp1-labelled pupal cells do not have a regular morphology and are not suitable for automatic cell counting with DeadEasy or Imaris Spot Function; thus, volume intensity measurements were made instead. Variations in fixation, staining, confocal settings, and ageing of the confocal lasers over time can affect the quality of anti-Dcp1 staining. Most importantly, all genotypes for each experimental block (i.e. loss of function or gain of function) were handled, tested, and scanned as close in time as possible, and confocal settings were maintained constant within each of these groups. To analyse Dcp1 signal volume intensity using Imaris (Figs 5 and 6A,B; Fig. S8), a 3D region of interest (ROI) was selected for either the lamina or optic lobe minus lamina, followed by creating a surface to mask the ROI. Next, the Wizard Wand was selected to render the volume ROI, setting a threshold to match the DCP-1 signal whilst eliminating small non-specific background dots. Care was taken to ensure that samples were of equivalent quality prior to processing and that the thresholding worked consistently across samples. Once adjusted, the Split Touching Objects feature was enabled to isolate the DCP-1 signal from background. ROI volume signal intensity was selected.

#### Automatic cell counting

Nuclei labelled with histone-YFP did not require antibody staining. 3D ROIs through the stack of confocal images covering the entire optic lobe of each specimen were selected using Imaris. For automatic cell counting in the lamina (Fig. 6C,D), the ROI.tif files were then opened in ImageJ, and automatic cell counting was carried out using DeadEasy Optic Lobe, as previously described (Li et al., 2020), and publicly available at UBIRA: https://edata.bham.ac.uk/1213/. For the medulla plus lobula complex (Fig. 6E,F), there were too many closely adjacent cells for DeadEasy or Imaris to count unambiguously; therefore, signal intensity was measured instead.

#### Quantification of signal intensity using Fiji (Fig. 6E,F)

Maximum projections of optical sections through the anterior (corresponding approximately to the medulla) or posterior (corresponding approximately to the lobula complex) optic lobes mounted horizontally were generated. An ROI was traced around the optic lobe excluding the lamina, and signal intensity [integrated density (IntDen)] was measured using the ‘Measure’ tool in Fiji. ROI IntDen was normalised by subtracting the background IntDen, obtained from the mean intensity density of a small ROI within the tissue but not containing hisYFP+ cells multiplied by the ROI area.

#### Lamina L1 axonal misrouting

Lamina L1 neurons were visualised with *L1-splitGAL4>GFP*, and dissected pupal CNSs were counterstained with 24B10 to visualise all photoreceptor axons. Using Fiji, we generated Z-projections of one to five optical 1 µm sections to trace individual L1 projections. 24B10+ photoreceptors R7 and R8 project into the medulla, arranged in neat columns that do not intersect with each other. L1 neurons target medulla layers M1 and M5. If the axon of an L1 neuron was constrained within a single column, this was considered ‘a wild-type phenotype’. If the axon crossed into neighbouring columns on either side, this was considered ‘a misrouting phenotype’, as in Millard et al. (2007). Scoring was carried out manually.

#### Quantification of L1 dendrite volume

Amira was used to measure the volume of the L1 neuron dendrites in 3D. Stacks of confocal images of L1 neurons were imported into Amira. Within the segmentation section, the magic wand was used to select the dendritic spines, and thresholding was applied to faithfully trace them across the stack. Following the selection, volume retendering was applied to select and measure the full dendrite volume. Dendrite volume signal intensity was selected.

### Statistical analysis

Statistical analysis was carried out using GraphPad Prism. The confidence interval was 95%, and significance was set at *P*<0.05. For quantitative continuous data, D'Agostino or Shapiro–Wilk normality tests were carried out. Data that were normally distributed were analysed using Student’s *t*-test for comparisons between two genotypes. When more than two samples were being compared, equality of variances was tested (Levene's or Bartlett's test), and if equal, one-way ANOVA was used, followed by a *post hoc* Dunnett test for multiple comparisons to a fixed control. When normally distributed data did not pass the equality-of-variance test (Brown–Forsythe), Welch ANOVA was used, followed by a *post hoc* Dunnett test for multiple comparisons to a fixed control. For quantitative data that were not normally distributed, Kruskal–Wallis was used for comparisons between more than two genotypes, followed by *post hoc* Dunn's test for multiple comparisons to a fixed control. Categorical data were analysed with Fisher's exact tests and *post hoc* Bonferroni multiple comparison corrections. For further details, *P*-values, genotypes and sample sizes, please see Table S4.

## Supplementary Material



10.1242/biolopen.062837_sup1 Supplementary information

Table S4. Statistical Analysis

Table S5.
