# Supplementary material for: The neurotrophin DNT-2 via the Toll-2 receptor regulates neuronal survival and morphology during visual system development
Source: Biol Open. 2026 Sep 17;15(9):bio062837. doi: 10.1242/bio.062837 (PMC13617978; doi:10.1242/bio.062837)
Supplement: Supplementary file 1 [file biolopen-15-062837-s1.pdf]

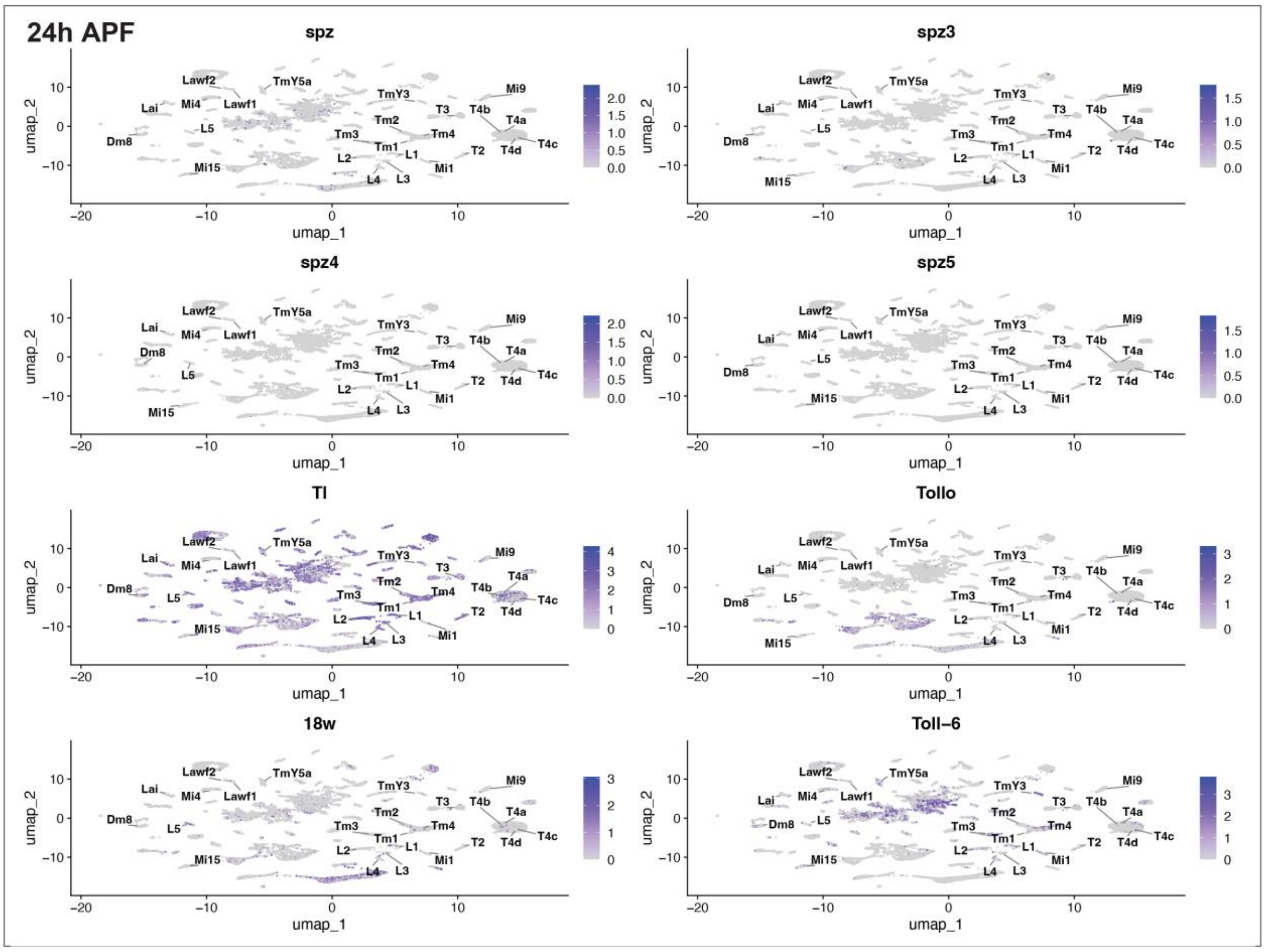

Fig. S1. scRNAse analysis at 24h APF (Kurmangaliyev et al, 2020 dataset)

Biology Open • Supplementary information

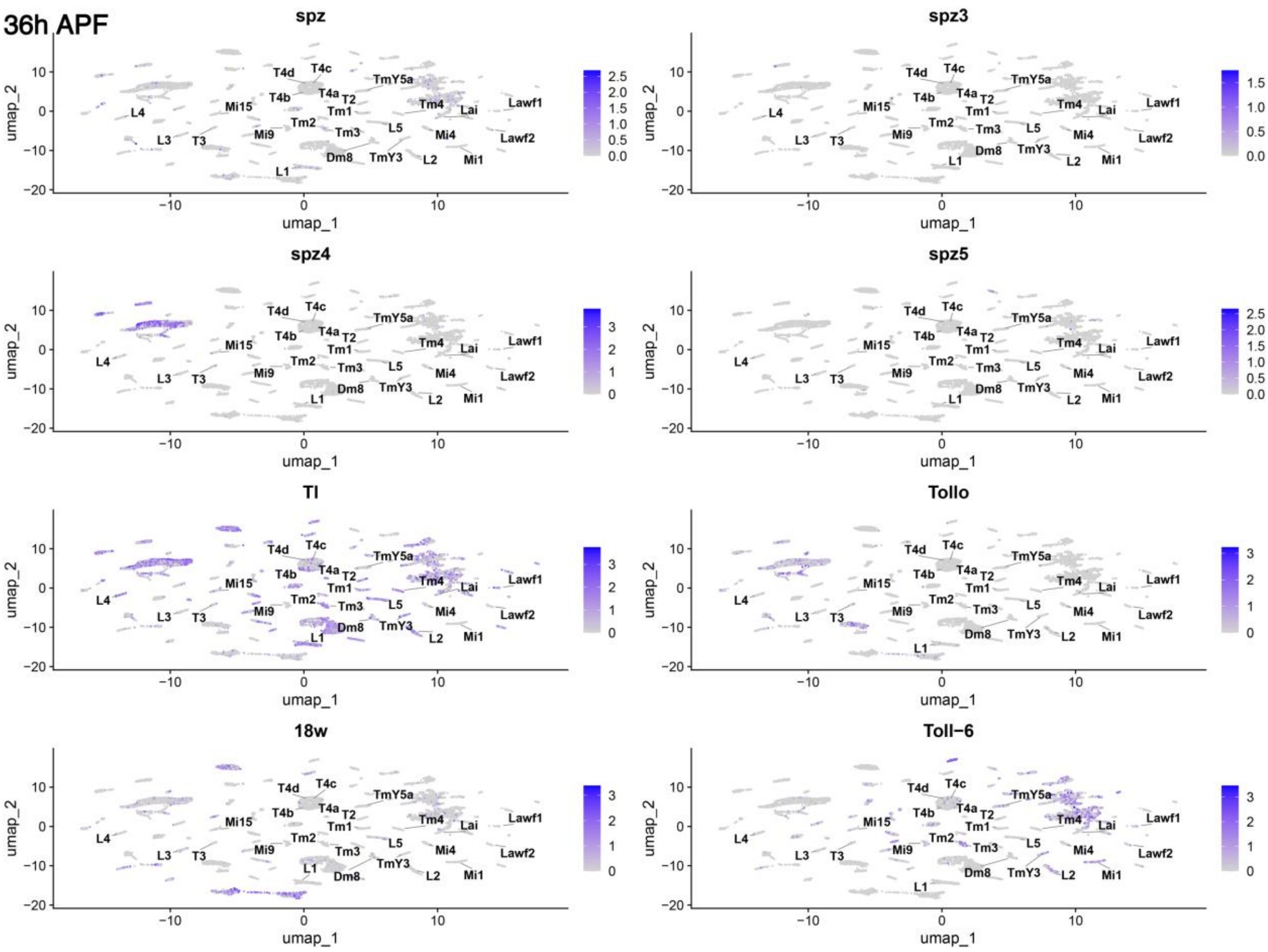

Fig. S2.

Biology Open • Supplementary information

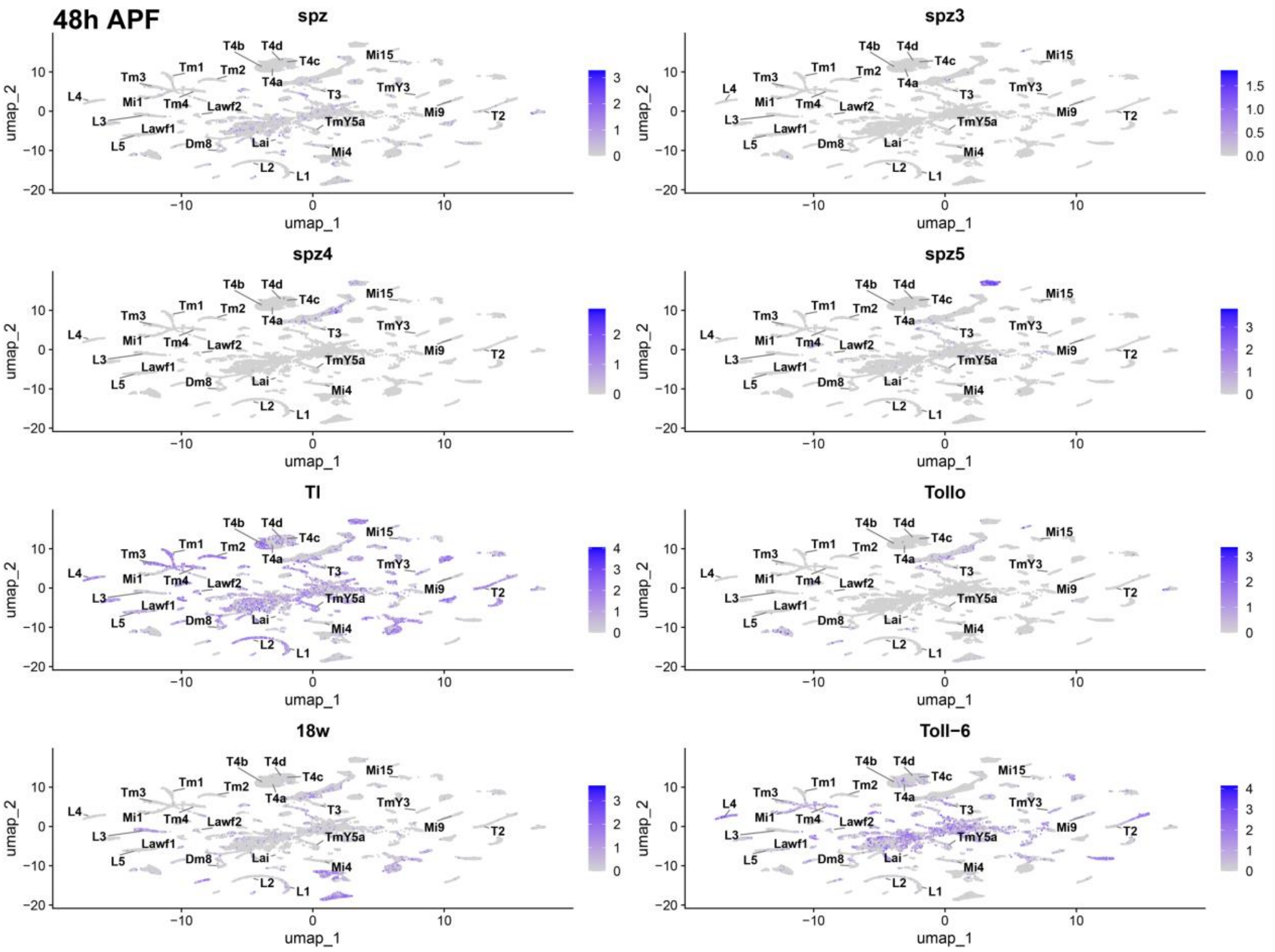
48h APF
spz
spz3
spz4
spz5
Tl
Tollo
18w
Toll−6
T4b
T4d
T4c
Mi15
Tm1
Tm2
Tm3
T4a
L4
TmY3
T3
Mi1
Tm4
Lawf2
Mi9
T2
L3
TmY5a
Lawf1
L5
Dm8
Lai
Mi4
L2
L1
umap_1
umap_2

Fig. S3.

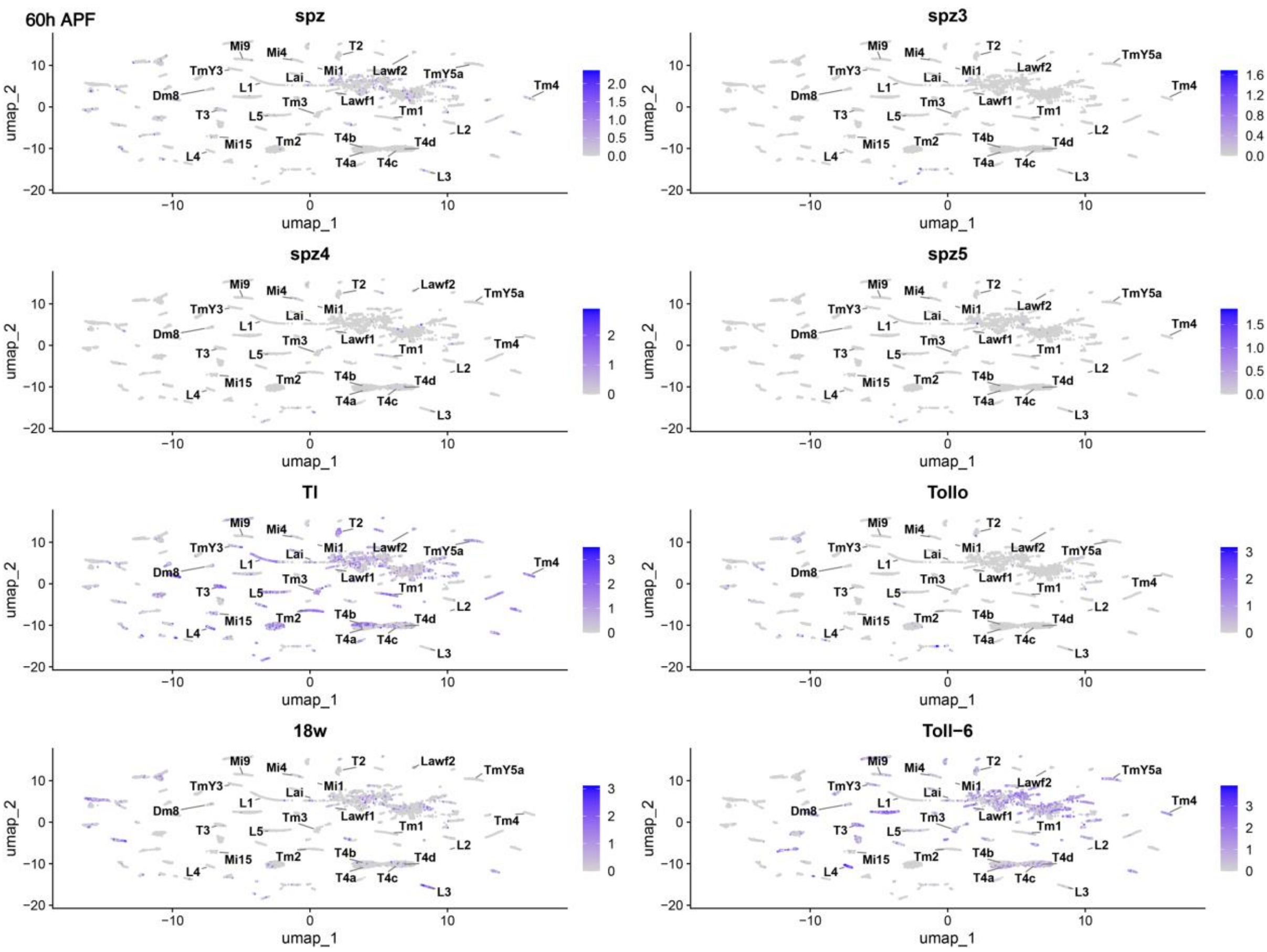

Fig. S4.

Biology Open • Supplementary information

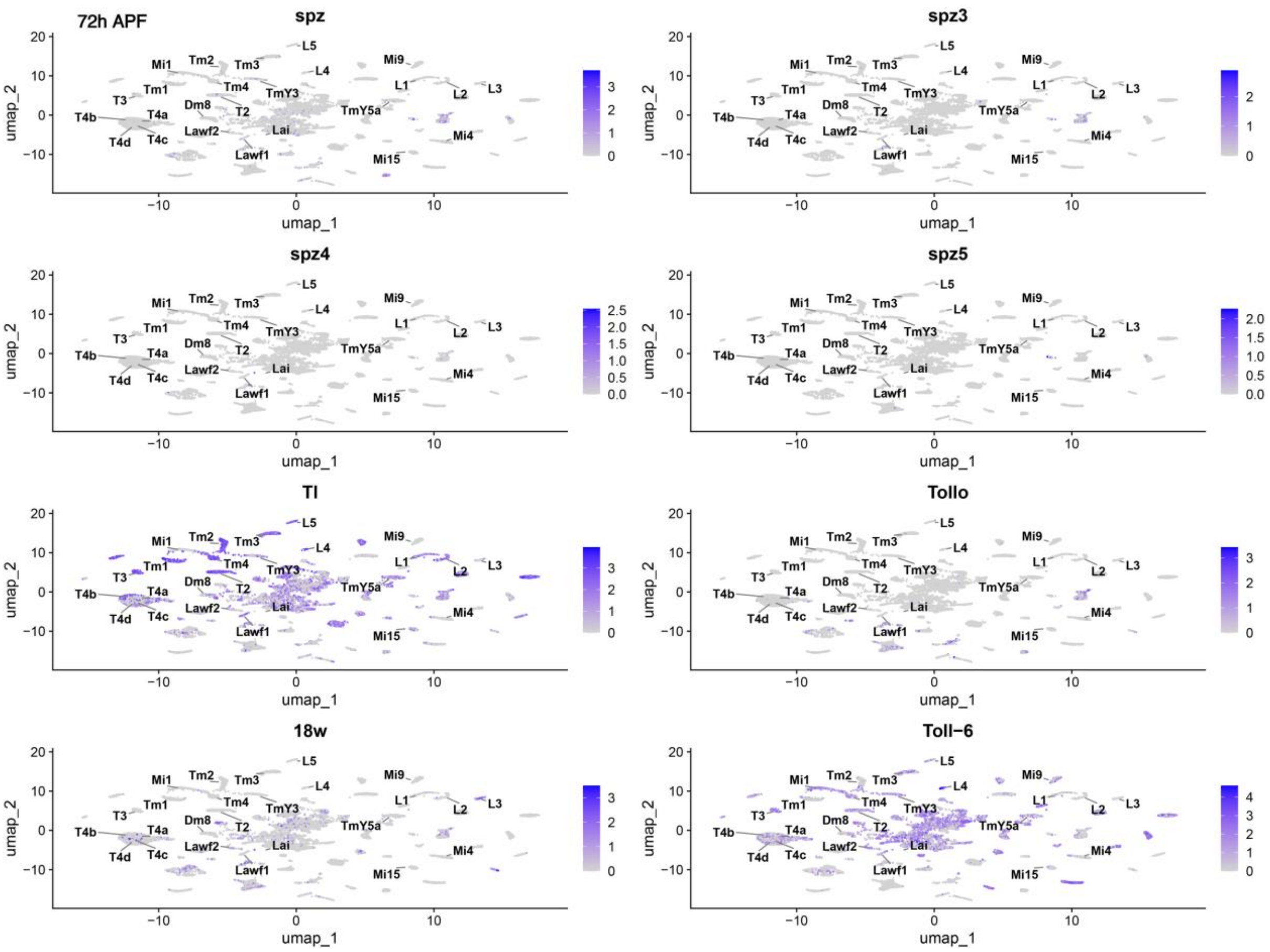

Fig. S5.

Biology Open • Supplementary information

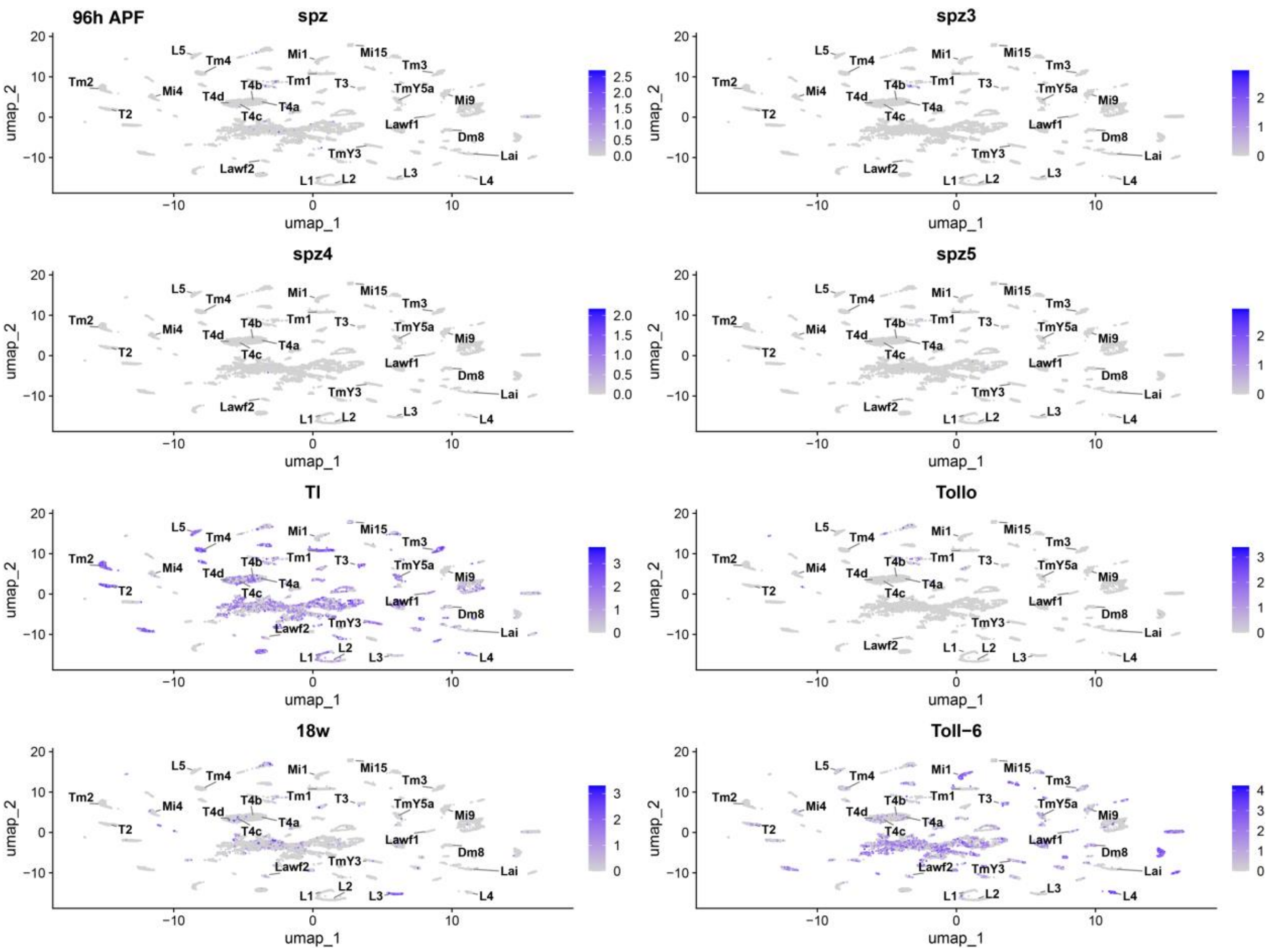

Fig. S6.

Biology Open • Supplementary information

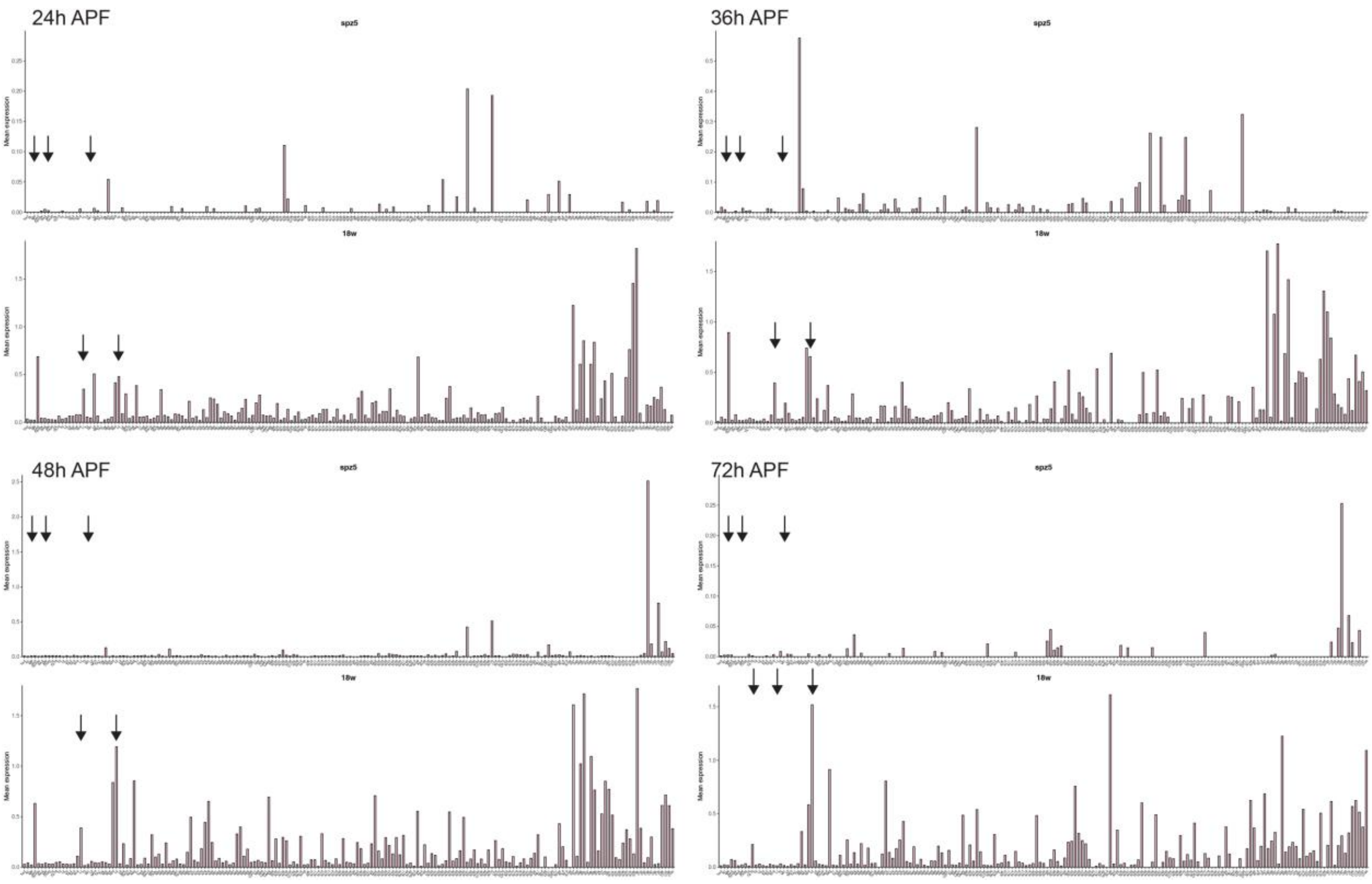

Fig. S7. scRNAseq: expression levels of spz-5/DNT-2 and 18w/Toll-2 over time in each cell type (Kurmangaliyev et al dataset)

Biology Open • Supplementary information

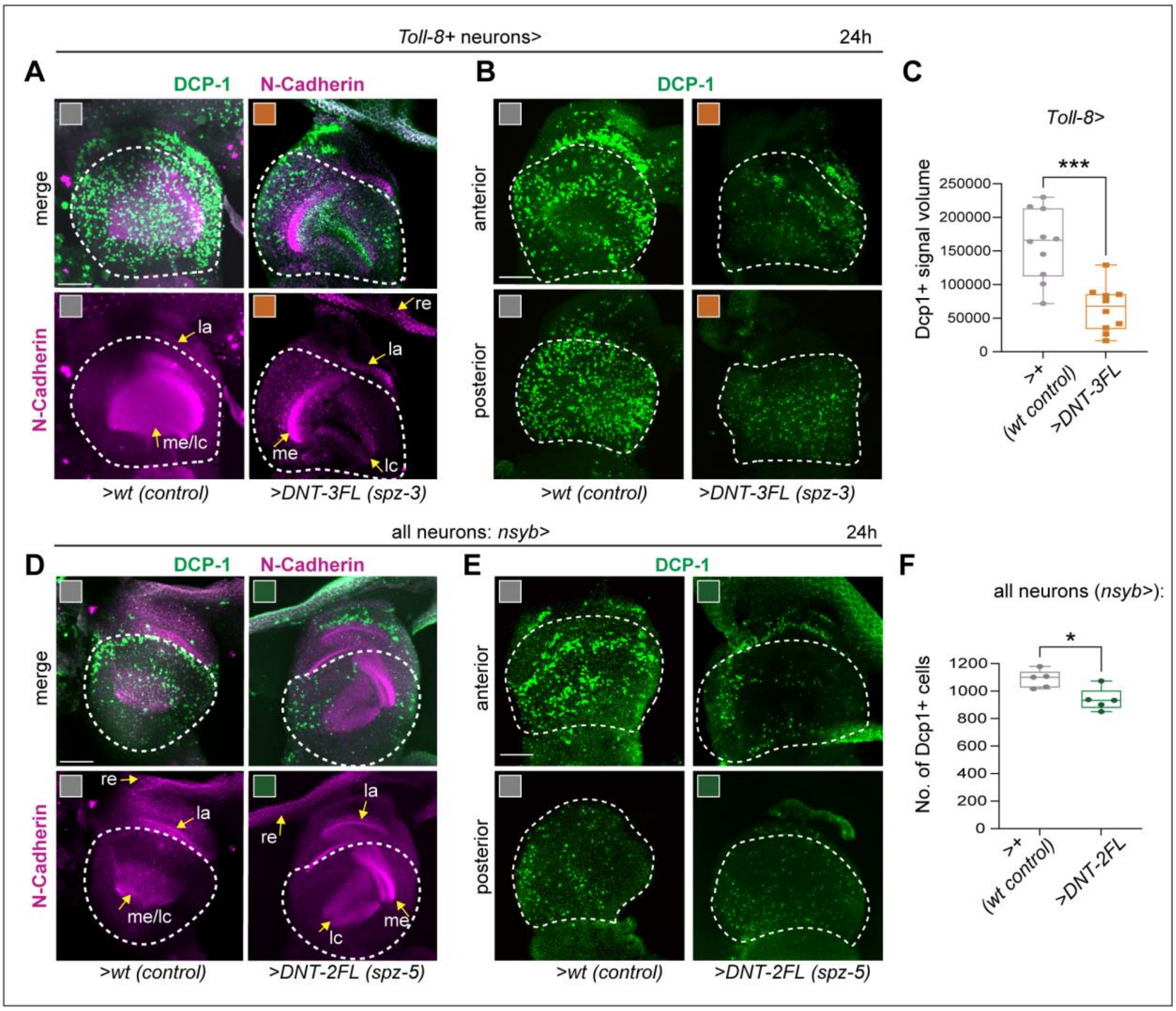

Fig. S8. DNT-2 and DNT-3 can maintain cell survival during optic lobe development also outside the lamina

Biology Open • Supplementary information

Table S1. Stock list

What?	Genotype	Source
Oregon	Oregon R	
nsybGAL4 on 3rd	w; nsybGAL4 attp2	BDSC#39171
10XUASmyrGFP on 2nd	P{10XUAS-IVS-myr::GFP}attP40	BDSC# 32198
10XUASmyrGFP on 3rd	P{10XUAS-IVS-myr::GFP}attP2	BDSC# 32197
10XUASmyrGFP on X	P{10XUAS-IVS-myr::GFP}su(Hw)attP8	BDSC# 32196
UASHistoneYFP on 2nd	w; UAS histone YFP (2)	
UAS-6xmCherry	y[1] w[*]; wg[Sp-1]/CyO, P{Wee-P.ph0}Bacc[Wee-P20]; P{y[+t7.7] w[+mC]=20XUAS-6XmCherry-HA}attP2	BDSC# 52268
MCFO on 3rd	w hsFLP:PEST ;; HA-V5-FLAG-OLLAS MCFO	BSC #64086
UAS mCD8 GFP on 2nd	w; UAS mCD8 GFP	
Toll-1 Gal4 on 3rd	w;+/(CYO);Toll-1-T2A-Gal4 [CRISPR]/ TM6B	Hidalgo lab Singh et al 2025
Toll-2 Gal4 on 2nd	yw; Toll-2^{pTV-}Gal4/CyO	Hidalgo lab ; Li et al 2020
Toll-6 Gal4 on 3rd	yw; Toll-$6^{MI02127}$ Gal4/TM3	Hidalgo lab Li et al 2020
Toll-8 Gal4 on 3rd	W; $Tollo^{MD806}$Gal4/ TM6B	BDSC #36548
DNT-2 Gal4 on 3rd	w; DNT-2 [CRISPR] T2A-GAL4/MKRS	Hidalgo lab Sun et al 2024
spz-3 GAL4 on 2nd	w; spz-3-T2A-GAL4/CyO	This work; Hidalgo lab
spz-4 GAL4 on 2nd	yw; spz-$4^{MI15678}$-T2A-Gal4 / SM6a	This work; Hidalgo lab
spz-3^{27} LOF mutant	w; spz-3^{27}/ CyO	This work; Hidalgo lab
spz-3^{46} LOF mutant	w; spz-3^{46}/ CyO	This work; Hidalgo lab
DNT-2^{18} LOF mutant	DNT-2^{18}	Hidalgo lab ; Sun et al 2024
DNT-2^{37} LOF mutant	DNT-2^{37} / TM6B	Hidalgo lab; Sun et al 2024
UAS-spz-3FL on 3rd	w; UAS spz-3FL Full length @attP2	This work; Hidalgo lab
UAS-spz-3CK on 3rd	w; UAS spz-3 Cysknot 22A @86FA	This work; Hidalgo lab
UAS-DNT-2FL on 3rd	w; UAS DNT-2 FL 22A @86FA	Hidalgo lab; Zhu et al 2008
UAS-DNT-2CK on 3rd	UAS DNT-2 CK6A @86FA	Hidalgo lab ; Zhu et al 2008
UAS-Toll-2RNAi on 2nd	W; UASToll-2RNAi[GD36305]/CyO	VDRC36305
UAS-DNT-2-RNAi on 3rd	W; UAS-DNT-2RNAi[VDRC41295]	VDRC41295]
L1-Split-GAL4	R48A08AD;R66A01DBD	Tuthill Reiser Lab
GRASP	w[*]; P{w[+mC]=lexAop-nSyb-spGFP1-10}2, P{w[+mC]=UAS-CD4-spGFP11}2; MKRS/TM6B	BDSC#64315
Retrotango	y[1] w[*] P{y[+t7.7] w[+mC]=QUAS-mtdTomato-3xHA.S}su(Hw)attP8; P{y[+t7.7] w[+mC]=retro-Tango(panneuronal)}attP40/SM6b; P{y[+t7.7] w[+mC]=10xUAS-retro-Tango(ligand)-P2A-EGFP-F}attP2	BDSC#99661

Biology Open • Supplementary information

Table S2. List of primers

Original name	Construct or what for	Primer Sequence 5→3
Sense gRNA	Spz3-T2A-Gal4	GTCGTTTGGGTCGCTCGATGTCT
Antisene gRNA	Spz3-T2A-Gal4	AAACAGACATCGAGCGACCCAAAC
Fw-Spz3 5'HA	Spz3-T2A-Gal4	GGTATACCGGTCGAATAAGTGACTCAAGCAGAC
Rv-Spz3 5'HA	Spz3-T2A-Gal4	ATAGCGGCCGCAGTATCTGAGTTTTGGTCTT
Fw-Spz3 3'HA	Spz3-T2A-Gal4	tatGGTACCTCTTGGCGCGGCACTCAAGT
Rv-Spz3 3'HA	Spz3-T2A-Gal4	cacACTAGTCTCATGCCGGCGAACCTATC
SP-EcoRI-Fw	UAS-Spz3-CK (SP of spz-5)	CGGAATTCATGCAAATCGACGGCGAATGA
SP-BglII-Rev	UAS-Spz3-CK (SP of spz-5)	GAAGATCTCGAGCTGTGGGCGGCTACTGT
Spz-3-CK-BglII	UAS-Spz3-CK	GAAGATCTGCCGGAGGAAGTCGAAATAGA
Spz-3-CK-XhoI	UAS-Spz3-CK	CCGCTCGAGCAGAGTCAGGTAATCTAGGGA
attB1 F spz-3FL	spz3FL pDONR	GGGGACAAGTTTGTACAAAAAAGCAGGCTCGCTAGCATATTTCGCACGCCC
attB2 R spz-3FL	spz3FL pDONR	GGGGACCACTTTGTACAAGAAAGCTGGGTCGGGATTACATCTACAGACAC
UAS-Spz3-FLGW (F) New	UAS-Spz3-FL	GAGGGTTCGTACTCCCGTTA
UAS-Spz3-FLGW (R) new	UAS-Spz3-FL	CGGCTTCGACGGTCTCCCCG

Biology Open • Supplementary information

Table S3. Key resources: List of antibodies

Antibody	Donor	Working dilution	Supplier
**Primary**			
Anti-GFP	Rabbit	1:250	Thermo Fisher A11122
GRASP-specific anti-GFP	Mouse	1:200	Sigma/Merk G6539
Anti-Ncadherin	Rat	1:250	DSHB-Iowa
Anti-24B10	Mouse	1:250	DSHB-Iowa
Anti-DCP-1	Rabbit	1:250	Cell Signalling 9578S
Anti-HA	Chicken	1:100	Aves
Anti-Bsh	Guinea pig	1:500	Kind gift from Larry Zipursky
Anti-Svp	Mouse	1:10	DSHB-Iowa
Anti-Erm	Rabbit	1:100	Kind gift from Uwe Walldorf
**Secondary**			
Anti-rabbit 488	Donkey	1:250	Molecular Probes A21206
Anti-rabbit 546	Goat	1:250	Molecular Probes
Anti-mouse 647	Goat	1:250	Molecular Probes A21236
Anti-rat 647	Goat	1:250	Molecular Probes A21247
Anti-Chicken 647	Goat	1:250	Molecular Probes A21103
Anti-Guinea Pig 488	Goat	1:250	Invitrogen A11073

Table S4. Statistical Analysis

Available for download at
https://journals.biologists.com/bio/article-lookup/doi/10.1242/bio.062837#supplementary-data

Table S5.

Available for download at
https://journals.biologists.com/bio/article-lookup/doi/10.1242/bio.062837#supplementary-data

Biology Open • Supplementary information
