# Supplementary material for: The neurotrophin DNT-2 via the Toll-2 receptor regulates neuronal survival and morphology during visual system development
Source: Biol Open. 2026 Sep 17;15(9):bio062837. doi: 10.1242/bio.062837 (PMC13617978; doi:10.1242/bio.062837)
Supplement: Supplementary file 2 [file biolopen-15-062837_review_history.pdf]

Biology Open
Review COMMONS

The neurotrophin DNT-2 via the Toll-2 receptor regulates neuronal survival and morphology during visual system development

Naser Alshamsi, Francisca Rojo-Cortes, Jervis Fernandes, Callum Perrett, Bangfu Zhu, Samaher Fahy, Guiyi Li, Anna Lassota, Marta Moreira and Alicia Hidalgo
10.1242/bio.062837

Editor: Tristan Rodríguez

Review timeline

Submission to sister journal:	2 June 2026
Editorial decision at sister journal	6 July 2026
Transfer to Biology Open:	28 July 2026
Accepted:	29 July 2026

Reviewer 1

Evidence, reproducibility and clarity

Alshamsi et al. investigate the role of Drosophila neurotrophins (DNTs) and their Toll receptors in regulating neuronal apoptosis during optic lobe development. The authors provide compelling evidence that different DNTs and their Toll receptors are expressed in optic lobe neurons, and their activity regulates neuronal survival during optic lobe development. They further show that disruption of DNT/Toll signaling impacts neuronal morphologies.

Comments

ABSTRACT

"Over-expression of DNT-3 (spz-3) and DNT-2 (spz-5) could rescue natural occurring cell death, whereas their loss of function caused cell death, showing that DNT-3 and DNT-2 can, and are required to, promote cell survival during optic lobe development."

I find it more appropriate to say that OE prevents naturally occurring cell death because it inhibits a normal physiological process."rescue" would only be correct if there were an experimental or genetic loss (e.g., deletion of a survival factor) and you are restoring normal survival levels "Importantly, DNT-2 is expressed in Mi1 neurons and Toll-2 in connecting L1 neurons. We show that DNT-2 functions in concert with Toll-2, as Toll-2 RNAi knock-down prevented the rescue of apoptosis by DNT-2 over-expression and all Toll-2+ neurons were lost in DNT-2 mutants."

I find this sentence very difficult to follow.

i suggest moving "Importantly, DNT-2 is expressed in Mi1 neurons and Toll-2 in connecting L1 neurons." to the next sentence.

"by specifically investigating the Mi1 (DNT-2+) and L1 (Toll-2) synaptic partners", alterations in DNT-2 or Toll-2 expression levels impaired connectivity of L1 neurons at the M1 medulla layer and altered dendritic morphology of L1 neurons.

"As DNT-3 (spz-3) and DNT-2 (spz-5) are expressed in the medulla and they could influence both lamina and medulla neurons, this suggests that their function maintaining cell survival could enable

© 2026. Published by The Company of Biologists under the terms of the Creative Commons Attribution License (https://creativecommons.org/licenses/by/4.0/).

the stabilisation or alignment of connected neurons across medulla columns." influence what? this is very vague and needs a temporal understanding of when neurons die, synapses are formed, and consider the phenotypes of the mutants and RNAi experiments.

INTRODUCTION

"Neuronal survival is maintained by neurotrophic factors secreted in limited amounts by target cells, leading to the survival of only those neurons that receive trophic support (LeviMontalcini, 1987, Davies, 2003)."

Perhaps the authors can be more precise, e.g. One mechanism by which Neuronal survival is regulated is through neurotrophic factors secreted in limited amounts by to be synaptic partners and adjacent cells

"In this context, if neurotrophism is fundamental for nervous system development, it could have been enabled by evolutionarily conserved molecular mechanisms."

I think the authors want to suggest that given the fundamental and widespread role of neurotrophism in nervous system development, it remains unknown if it relies on evolutionarily conserved molecular players.

"Neurotrophins - NGF, BDNF, NT3, NT4 - are the main growth factors maintaining neuronal survival in the vertebrate nervous system (Levi-Montalcini, 1987, Lu et al., 2005). Importantly, they can also promote cell death, depending on context (Lu et al., 2005). They can promote cell survival via their Trk receptors and ERK and AKT downstream and via p75NTR and NFB downstream, or cell death via p75NTR, Sortilin, and JNK signalling instead (Lu et al., 2005). mechanisms."

The authors should avoid the repeated use of "they"

"There are six spz and nine Toll paralogous genes in Drosophila, which could play distinct functions. In fact, at least full-length DNT-1 and Toll-1 can promote cell death instead, and at least Toll-6 can promote either cell survival or cell death, depending on context (Foldi et al., 2017, Singh et al., 2025, Zhu et al., 2008). Importantly, mature DNT-1 and DNT-2 with Toll-6 and Toll-7 are required for and can promote neuronal survival during circuit formation in the embryonic ventral nerve cord (McIlroy et al., 2013, Zhu et al., 2008)."

I find this paragraph difficult to follow. I suggest the following editing, which the authors might want to consider:

"There are six spz and nine Toll paralogous genes in Drosophila, which could play distinct functions. In fact, at least full-length DNT-1 and Toll-1 can promote cell death instead, while and at least Toll-6 can promote either cell survival or cell death, depending on context (Foldi et al., 2017, Singh et al., 2025, Zhu et al., 2008). Importantly, mature DNT-1 and DNT-2 with Toll-6 and Toll-7 are required necessary and sufficient to promote for and can promote neuronal survival during circuit formation in the embryonic ventral nerve cord (McIlroy et al., 2013, Zhu et al., 2008).

"During this time (24-50h APF), connectivity between photoreceptors, lamina and medulla neurons is established; this is followed by medulla neurons connecting to lobula neurons;"

I find this sentence misleading, if not incorrect. If by connectivity the authors mean synaptogenesis, for all that is known, synaptogenesis has been shown to occur from mid-pupal development (P50) onwards If by connectivity, the authors mean the targeting of specific neuropiles and layer organization, it is also incorrect that lamina and medulla organization precedes the connectivity between medulla and lobula neurons. These processes are all concurrent. Can the authors please clarify?

"and by 72h APF cell death has greatly diminished and synaptogenesis completes connectivity patterns, in preparation for adult eclosion at 96h APF (Millard and Pecot, 2018, Melnattur and Lee, 2011, Hadjieconomou et al., 2011, Kurmangaliyev et al., 2020)"

© 2026. Published by The Company of Biologists under the terms of the Creative Commons Attribution License (https://creativecommons.org/licenses/by/4.0/).

Kurmangaliyev et al., 2020 is probably not an appropriate citation here as it mostly deals with transcriptional programs of circuit assembly in the developing optic lobe 02/06/2026, 12:04 RC-2025-03128R https://reviewcommons.msubmit.net/cgi-bin/main.plex?el=A7PK7BHHk5B4Czzq5CG7A9ftdO7r9jpB7vA7GLG1ZTRYgZ 2/14 "Thus, 24-48h APF is a critical period to maintain necessary lamina and medulla neurons alive in the optic lobe."

Perhaps the authors want to revisit this sentence and explicitly say that 24-48h APF is a period where apoptosis defines cell numbers.

"The development of the Drosophila visual system has been well described (Holguera and Desplan, 2018, Melnattur and Lee, 2011, Millard and Pecot, 2018, Hadjieconomou et al., 2011, Behnia and Desplan, 2015)."

I find that Behnia and Desplan, 2015 is not appropriate, as it is a review that describes the characterization of neuronal circuits underlying visual modalities in the fly brain, and not their development. The following reviews dealing with different aspects of neurogenesis, neuropile development and circuit formation are likely more relevant:

Bakshi et al Current Opinion in Neurobiology 2025 Malin et al PNAS 2021 Ngo Dev Bio 2017

"R7 and R8, together with lamina neurons target to medulla layers M6 and M3, respectively, organizing into medullar columns that respond to the same point in visual space and maintain retinotopy."

I find this sentence misleading because lamina neurons do not target M6 and only L3 targets M3.

"Medullar interneurons also form connections across multiple layers, where each layer represents different visual features (Fischbach and Hiesinger, 2008, Millard and Pecot, 2018)."

Here, Behnia and Desplan, 2015, Matsliah et al Nature 2024, Borst and Groschner, Annu. Rev. Neurosci. 2023, and even Schnaitmann et al J Comp Physiol A Neuroethol Sens Neural Behav Physiol 2020 are better references that review feature detection and circuit organization in the optic lobe.

Neurons within the lobula complex integrate signals from the medulla and project to the optic glomeruli in the central brain and motor outputs to enable appropriate behavior (Behnia and Desplan, 2015, Borst et al., 2020, Courgeon and Desplan, 2019b)."

I find Courgeon and Desplan, 2019b is not very appropriate here, as it reviews the coordination of neural patterning in the Drosophila visual system. More adequate and relevant manuscripts and reviews are Wu et al. elife 2016, Tanaka and Clark, 2022, Lapoetke et al. Neuron 2022, even Zhao et al. elife 2024.

"Spz-5 is well known as Drosophila neurotrophin-2 (DNT-2), and as Spz-3 has been proposed to have neurotrophin functions which we expand on and demonstrate here, we refer to Spz-3 as DNT-3 (Zhu et al., 2008, Coutinho-Budd et al., 2017, Sun et al., 2024, Ballard et al., 2014, Ulian-Benitez et al., 2017)."

This last paragraph of the introduction seems out of place, and partly redundant with page 3. Perhaps the authors would like to finish the introduction with a paragraph highlighting the major findings and conceptual advance of the manuscript? This seems to be a good and natural way of following their previous sentence "Here, we asked whether neurotrophin family ligands encoded by DNTs (spzs) and their Toll receptors could regulate cell survival during neural circuit formation, in the Drosophila pupal optic lobe."

RESULTS

I suggest the following edit:

© 2026. Published by The Company of Biologists under the terms of the Creative Commons Attribution License (https://creativecommons.org/licenses/by/4.0/).

To ask whether DNTs (spzs) are expressed in the pupal optic lobe, we generated T2A-Gal4 driver lines for spz-1,3,4,5 fly lines, crossed them to 10xUASmyrGFP or 20UAS6xmCherry reporter flies, and analysed resulting progeny optic lobes during development and in the adult with anti-GFP antibodies, as required (Figure 1A).

Also, If the lines were also crossed with mcherry, mentioning anti-GFP antibodies is incomplete.

"spz-1MIO2318-T2A>myrGFP and spz-1MIO2318-T2A>6xmCherry revealed expression in a few centrifugal neurons in the lobula complex that projected to the lamina,"

At which stage?

"subsequently medulla neurons and abundant arborisations into the lobula complex and medulla." Subsequent to what? I am sorry, but I don't understand this description.

"Expression from spz4MI5678 -T2A->myrGFP was not detected until 72h APF" Data before 72h APF is missing Where was it expressed? Which cell types? Which neuropiles?

"And then was found in the medulla and lobula complex and followed by the trachea."

At which stage?

What was followed by the trachea? I think the authors mean that at later staged (in the adult) expression was restricted to the trachea in both medulla and lobula.

"spz-3-T2A>6xmCherry (hereby named DNT-3) was highly expressed in non-neuronal retinal cells and medulla neurons;"

At what stage?

“And subsequently in the trachea and possibly glia."

The authors could and should explain how they reach this conclusion. Given that no cell type specific markers were used, this identification was likely based on morphological features.

"Finally, DNT-2-T2A>6xmCherry (spz-5) was found in medulla neurons, which could be tentatively identified as Mi1 medulla neurons (Nern et al., 2025) by 48h APF, and this pattern was maintained."

The authors suggest these neurons are Mi1 based on what?

Also, Fischbach's Cell Tissue Res (1989) seminal paper is probably worth mentioning.

"Abundant cells expressed spz-1 in the lobula complex (Figure 1B, left) and medulla (Figure 1B, right), and DNT-3 (spz-3) and DNT-2 (spz-5) in the medulla (Figure 1B) during optic lobe development. DNT-3 (spz-3) and DNT-2 (spz-5) were expressed in distinct nonneuronal cells in the retina, seen in Multi Colour Flip Out (MCFO) clones (Figure 1C)."

This sentence is misleading. These experiments allow the authors to conclude that spz+ neurons innervate these neuropiles. It does not allow the authors to conclude that spz molecules localize to the neuropiles.

The authors should revise these claims in the main text and relevant figure legends.

"To visualize the distribution of Tolls in the optic lobes during pupal development, we used the GAL4 lines previously described (Li et al., 2020), driving expression of the reporter myrGFP (Figure 2A)."

Same comment as above

© 2026. Published by The Company of Biologists under the terms of the Creative Commons Attribution License (https://creativecommons.org/licenses/by/4.0/).

"Using MCFO clones as well as myrGFP, we could identify some of the Toll-8+ neurons as Lawf1, feedback neurons projecting from the medulla to the lamina (Figure 2B), and L2 and L4 lamina neurons (Figure 2B, C); Toll-6+ cells to include L3 and L4 lamina neurons (Figure 2B,D); and Toll-2+ neurons as L1 lamina neurons which target to M1 and M5 medulla layers and L3 lamina neurons that project to M3 (Figure 3B,E) (Hakeda-Suzuki and Suzuki, 2014, Behnia et al., 2014)."

Hakeda-Suzuki and Suzuki, 2014, Behnia et al., 2014 are not the most appropriate references. Instead, I suggest the authors should cite Fischbach's Cell Tissue Res.

"Overall, the expression in the scRNAseq dataset (Kurmangaliyev et al., 2020) of the spz ligands and Toll-8 (also known as Tollo) data were less consistent with the cell biology data, whereas the expression of Toll-1, -2 and -6 confirmed cells seen with the cell-biology based reporters"

It is perhaps more accurate to refer to the cell-biology based reporters as translation reporters, which is what T2a based Gal4 drivers are.

"Most particularly, Toll-2 mRNA (synonym 18w) was found in L1 and L3 lamina neurons over time, plus also in L5 at 24h APF, and Toll-6 mRNA was found in L2, L3, L4 over time, plus also in L1 at 24h (Supplementary Figure 1-6)."

This sentence is difficult to read and could be considered poorly written for several reasons including:

- "plus also" is repetitive. "Plus" and "also" serve the same function.

- Inconsistent punctuation. The lack of commas before "and Toll-6 mRNA..." makes the sentence feel unbalanced.

-Vague time reference. "Over time" is imprecise. It's unclear whether it means during development, at multiple timepoints, or something else.

Also regarding scRNAseq analysis:

- the authors mention "We compared our reporter-based profiles with published scRNAseq datasets of the optic lobe through development (Kurmangaliyev et al., 2020, Ozel et al., 2021)" however the Ozel dataset doesn't seem to be used.

Also, from the Material and Methods section:

"The data were imported as a Seurat object, and cells corresponding to specific timepoints (e.g., 24 h, 36 h) were subsetted based on the provided metadata. Dimensionality reduction was carried out using principal component analysis (PCA), followed by Uniform Manifold Approximation and Projection (UMAP) embedding computed on the first 30 principal components. Cluster annotations provided by the original authors were used for all clusterlevel analyses and visualisations."

The Kurmangaliyev dataset is already processed. I am probably missing something here, but is not obvious to me why the authors performed PCA again.

"To conclude, at the time of naturally occurring cell death (0-48h APF), Toll-1 is highly expressed throughout the optic lobe; Toll-2, -6 and -8 are expressed in the medulla; Toll-8 and Toll-6 are prominently expressed in the lobula complex, and Toll-6 and Toll-2 are prominent in the lamina."

This is misleading. These experiments allow the authors to conclude that toll+ neurons innervate these neuropiles. It does not allow us to conclude that toll molecules localize to the neuropiles. The authors should revise these claims in the main text and relevant figure legends.

"To ask whether DNTs can promote cell survival in developing optic lobes, we overexpressed DNT-2 (spz-5) and DNT-3 (spz-3) and visualized dying cells with the apoptotic marker anti-Dcp1 at the peak of naturally occurring cell death (24h APF)."

© 2026. Published by The Company of Biologists under the terms of the Creative Commons Attribution License (https://creativecommons.org/licenses/by/4.0/).

These experiments were done using Toll8-Gal4 and nsyb-Gal4 drivers. What's nsyb-Gal4 expression during development? Is the expression of this driver consistent with the conclusions drawn from these experiments?
"To test whether DNT-2 could promote cell survival during optic lobe development, we overexpressed full-length DNT-2FL or cleaved DNT-2CK in all neurons with nsybGAL4. This reduced the incidence of Dcp1+ apoptosis in the lamina and outside the lamina too (Figure 3C-D and Supplementary Figure S9C,D)."

How do the authors explain that DNT-2CK reduced the number of Dcp1+ cells?

"We generated DNT-3 (spz-3) loss of function mutants by P-element mobilization. DNT-2 and DNT-3 loss of function mutants caused considerable cell debris in the medulla and lobula complex, which compromised the analysis in this region, so we focused on the lamina."

Perhaps, rather than stating that the mutants caused considerable cell debris, the authors could say that the mutants displayed considerable cell debris.

More importantly, I have concerns with the data from these experiments (Figure 3). Dcp1 signal volume intensity using Imaris. In all panels (A,C,E) the segmented images do not match the raw DCP1 staining, raising concerns on how much can one rely on this quantification.

Could this be because the Dcp1 staining shown is a single z plane and the segmentation is a 3d rendering? The authors should carefully and robustly explain this discrepancy which is present in all images where Dcp1 signal volume intensity was quantified.

Also, could the authors explain why the quantifications in Figure 3B and 3C differ by an order of magnitude (10×) from those in panel 3D? Please look at the WT control, there is a 10X difference in signal volume intensity.

"Toll-2pTVGAL4 flies are heterozygous mutant for Toll-2, and, remarkably, in combination with DNT-2 homozygous mutants resulted in semi-lethality, revealing a functional interaction between these two genes.

I cannot entirely follow this conclusion. I understand the authors propose that the combination of partial loss of Toll-2 and full loss of DNT-2 affects viability, more than either mutation alone. Is this what they mean? Can the authors comment on the viability of DNT-2 mutants?

"Macrophages loaded with HisYFP and distributed mostly between the retina and lamina could be observed across these samples (Figure 4C), suggesting they had engulfed dead cells"

How do the authors identify these YFP+ cells as macrophages?

"Together, these data show that DNT-2 functions as a ligand for Toll-2 to maintain the survival of neurons in the lamina, medulla and lobula complex during optic lobe development."

While the results from Figure 4 showing that DNT-2 acts as a ligand for Toll-2 to support neuron survival are solid (in particular panels C-F), it doesn't necessarily mean all neurons die directly due to loss of Toll-2 signaling.

It is plausible that Neurons that express Toll-2 die because they lose critical survival signals.

The death of these Toll-2-expressing neurons could then cause a cascade effect, where neighboring or connected neurons die indirectly due to loss of trophic support, disrupted circuits, or secondary damage.

So, the observed cell death in multiple regions may be a combination of direct effects on Toll2-positive neurons and indirect effects on other neurons.

© 2026. Published by The Company of Biologists under the terms of the Creative Commons Attribution License (https://creativecommons.org/licenses/by/4.0/).

"In the Drosophila pupa, connectivity of lamina to medulla neurons takes place at 30-48h APF, and between medulla and lobula complex at 60-70h APF (Kurmangaliyev et al., 2020, Millard and Pecot, 2018, Pecot et al., 2014, Hadjieconomou et al., 2011)."

I have the same comment as mentioned above regarding the timing of connectivity. If by connectivity, the authors mean the targeting of specific neuropiles and layer organization, it is incorrect that lamina and medulla organization precedes the connectivity between medulla and lobula neurons. These processes happen concurrently. Can the authors please clarify?

"Importantly, the expression of synaptic markers starts at 24h, peaks at 60h APF and spontaneous neuronal activity takes place at 48h APF, meaning that at least some neural circuits are already connected by this point (Kurmangaliyev et al., 2020)."

The correct placement of the reference to Kurmangaliyev et al. is after "peaks at 60h APF " A reference to Akin et al and Bajar et al when referring to PSINA is missing.

"Thus, the period of naturally occurring cell death overlaps with connectivity" I think the authors mean that the period of cell death is concurrent with the development of synaptic connectivity.

"L1 neurons normally project along columns that can be labelled with mAb24B10, and target to layers M1 and M5 of the medulla."

The authors should mention that 24b10 labels the photoreceptors, providing a spatial reference to identify medulla columns.

"Interestingly, Toll-2RNAi knock-down did not alter the phenotype caused by DNT- 112FL overexpression, and impaired targeting to the same extent as each genetic manipulation alone (Figures 5B,D)."

How do the authors interpret these results? And perhaps the authors would like to explain the rationale of overexpressing DNT-2fl in L1 neurons, that do endogenously express it. "

To conclude, these data show that DNT-2 and Toll-2 are required for appropriate connectivity of L1 neurons to target Mi1 medulla neurons at M1 medulla layer."

The authors characterize neuronal morphologies but do not directly assess connectivity using synaptic markers. While defective morphologies are likely to impact connectivity, the conclusion that DNT-2 and Toll-2 are required for appropriate connectivity should be tempered. The authors should revise their wording to reflect that their data support morphological defects rather than direct evidence of altered synaptic connectivity.

DISCUSSION

"In fact, throughout animal development, between 50% (e.g. in Drosophila) and 80% (e.g. in vertebrates) are lost to naturally occurring cell death"

"of neurons" is missing before "are lost"

"Consistently with these findings, we have shown that the survival of L1 neurons depends on DNT-2 functioning together with Toll-2."

The authors state that "the survival of L1 neurons depends on DNT-2 functioning together with Toll-2." It seems that what they intend to convey is that DNT-2 acts as a ligand for Toll-2. The text should be clarified to explicitly indicate this ligand-receptor relationship rather than implying a cooperative function.

"These data demonstrate that DNT-2 and Toll-2 function together in visual system development."

Since the authors did not use tub-GAL80 or another temporal control to restrict gene expression specifically to development, the observed phenotypes could reflect combined developmental and

© 2026. Published by The Company of Biologists under the terms of the Creative Commons Attribution License (https://creativecommons.org/licenses/by/4.0/).

adult effects. Throughout the text, the authors should revise their wording to acknowledge this limitation.

"Finally, interference with the normal levels of DNT-2 and Toll-2 also impaired axon targeting and dendritic morphology, consistently with the coupling between cell survival with connectivity."

The authors state that interference with normal levels of DNT-2 and Toll-2 "impaired axon targeting and dendritic morphology, consistently with the coupling between cell survival and connectivity." This statement seems tautological, as neurons that die cannot form connections. The authors should clarify whether they are referring to a specific mechanistic link beyond this obvious consequence.

"Our findings are consistent with prior reports that had shown the maintenance of cell survival to be required during neural circuit formation."

This statement seems tautological. It is generally expected that neurons must survive in order to contribute or be part of neural circuits. The authors should clarify if they are highlighting a specific mechanistic insight beyond this obvious requirement.

"In the medulla, Dm8 medulla neurons are produced in excess and are eliminated during connectivity to their R7 inputs (Courgeon and Desplan, 2019a). This is enabled by the cell surface molecular tags DIP in yDm8 binding Dpr11 in yR7, during synaptic matching (Courgeon and Desplan, 2019a)."

The statement that "Dm8 medulla neurons are produced in excess and are eliminated during connectivity to their R7 inputs" is both unclear and inaccurate. It is not evident what is meant by "during connectivity." Moreover, Courgeon and Desplan (2019a) show that Dm8 neurons undergo cell death before or by P40, prior to synaptogenesis. The authors should correct this statement and clarify the timing and mechanism of Dm8 neuron elimination.

"Importantly, the maintenance of cell survival takes place during connectivity, and enables synaptic matching between connecting neurons."

It is unclear what is meant by "during connectivity." Moreover, both Courgeon et al. (2019a) and Xu et al. (2018, 2022) show that these neurons (e.g., Dm8, Dm12, Dm14) undergo cell death before or by P40, prior to synaptogenesis. The authors should clarify the timing and mechanism of cell survival and revise this statement accordingly.

"By contrast, it has also been proposed that apoptosis plays a minor role in cell number control during visual system development, depending instead on cell proliferation and spatial patterning through Dpp/BMP signalling (Malin et al., 2024). However, those findings were based on events taking place at the larval third instar wandering stage, when proliferation and spatial patterning are prevalent, whereas apoptosis peaks in pupa."

It seems that the authors are trying to suggest that different mechanisms control cell numbers at different developmental stages: during larval neurogenesis (L3), cell numbers are regulated primarily by proliferation and spatial patterning, whereas in the pupal stage, neuronal survival via apoptosis plays a key role. If this is the intended point, it should be stated more clearly, as the current comparison to Malin et al. (2024) is confusing and does not make this distinction explicit.

"However, Toll-2 mutant MARCM clones generated in the pupa result in a dramatic loss of lamina neuron dendrites and aberrant axonal navigation in the medulla, as well as widespread neuronal loss (Li et al., 2020)."

This statement is puzzling. Neurogenesis occurs during the larval stage until P15, and MARCM requires progenitor cell division. The authors should clarify how MARCM clones were generated during pupation and provide the relevant experimental details in the Materials and Methods.

"Importantly, connectivity between L1 and medulla neurons takes place between 20-48h APF, during the period of naturally occurring cell death, and spontaneous activity in the optic lobe takes place at 48h, meaning at least some circuits are connected by then (Kurmangaliyev et al., 2020)."

© 2026. Published by The Company of Biologists under the terms of the Creative Commons Attribution License (https://creativecommons.org/licenses/by/4.0/).

The authors state that "connectivity between L1 and medulla neurons takes place between 20-48h APF," but no reference is provided for this timing. To my knowledge, no study has directly demonstrated this, so the authors should either provide supporting evidence or revise this statement. Additionally, citing Kurmangaliyev et al. (2020) for spontaneous activity in the optic lobe is not appropriate for this point, as PSINA was originally described by Orkun Akin.

"When altering DNT-2 or Toll-2 levels, L1 axonal terminals in the medulla were misrouted, rather than being confined to a single column. This is reminiscent of the phenotypes caused by alterations in Dscam and Fez levels (Millard et al., 2007, Peng et al., 2018)."

The authors note that altering DNT-2 or Toll-2 levels causes L1 axonal terminal phenotypes reminiscent of phenotypes caused by changes in Dscam and Fez levels (Millard et al., 2007; Peng et al., 2018). However, they only reference these previous studies without discussing whether there could be a shared mechanism. While these comparisons are interesting, the manuscript would benefit from either a deeper discussion of potential mechanistic links or a clear statement that the comparison is purely phenotypic.

"As DNT-2 is secreted in medulla neurons and Toll-2 is expressed along neurons that connect in the medulla (e.g. L1, Mi1, Tm3, Dm9, T4), DNT-2 could help keep connecting neurons together during dynamic cellular events in development."

This sentence is poorly written and vague. It is unclear what "keep connecting neurons together" means mechanistically. Likely, DNT-2 is secreted by postsynaptic medulla neurons (e.g., Mi1), whereas Toll-2 is expressed in neurons innervating the medulla (e.g., L1, Mi1, Tm3, Dm9, T4). The authors should rephrase this sentence to clearly convey their mechanistic and cellular interpretation.

FIGURES

Figure 1

Arrowheads point to what?

OL orientations should be described in the figure captions

Figure 5 T
he title of Figure 5 ("Altering the levels of DNT-2 and Toll-2 modifies L1 axon targeting at medulla M1 layer") is misleading. The correct layer targeting is preserved; what changes is the pattern of unicolumnar innervation. When DNT-2 or Toll-2 levels are altered, L1 neurons innervate multiple columns rather than maintaining their normal single-column specificity. The title should be revised to reflect that the defect is in columnar specificity rather than layer targeting.

Supplementary Figures

S1 to S6 Combined UMAPs showing the expression of spz-1, -3 (DNT-3),-4 and -5 (DNT-2) and Toll-1, -2 (18w), -6 and -8 (Tollo) in distinct cells over time.Information about which UMAP corresponds to which time point is missing

MATERIALS AND METHODS

Genetics. Please see S1 Table for the list of the stocks used and Table S3 for full genotypes for each experiment. Table S3 is not the full genotypes for each experiment. **This information is partly available in the Source Data excel file**

Significance

During development, neurons are initially produced in excess. One mechanism by which the final neuronal numbers are refined relies on trophic support, which maintains the survival of necessary neurons, while excess neurons are eliminated.

© 2026. Published by The Company of Biologists under the terms of the Creative Commons Attribution License (https://creativecommons.org/licenses/by/4.0/).

In the Drosophila optic lobe, a wave of apoptosis occurs during pupation, peaking at a critical period thought to be essential for establishing final neuronal numbers and supporting proper neural circuit formation. However, the mechanisms underlying this developmental process remain poorly understood. In this manuscript, Alshamsi et al. investigate this wave of apoptosis by examining the role of Drosophila neurotrophins (DNTs), which are encoded by spätzle (spz) paralogous genes and signal through Toll receptors to regulate neuronal survival during brain development.

The authors use translation reporters to demonstrate that DNTs and Toll receptors are differentially expressed across various neuronal types innervating all optic lobe neuropils during development. They then focus on DNT-3 and DNT-2, which they show to be necessary for controlling neuronal numbers, likely by maintaining neuronal survival during pupal stages.

Notably, the results reveal a previously uncharacterized interaction between DNT-2 and Toll2. The findings suggest that DNT-2 acts as a neurotrophic factor produced by medulla intrinsic neurons, binding to the Toll-2 receptor expressed in other neurons innervating the medulla. By examining Toll-2+ L1 neurons, which are postsynaptic in the lamina and presynaptic in the medulla, the authors provide compelling evidence that DNT-2/Toll-2 signaling regulates L1 neuronal numbers.

Interestingly, the authors also show that disruption of DNT-2/Toll-2 signaling affects L1 axonal and dendritic morphologies. However, the extent to which changes in neuronal survival and neuronal morphology are mechanistically or cellularly linked is not addressed. These findings are consistent with previous reports showing that DNTs and Toll receptors regulate neuronal survival in embryonic, larval, and pupal ventral nerve cords, as well as in the adult. Importantly, DNTs and Tolls can also promote cell death, highlighting their dual role in controlling neuronal number and circuit formation.

While the data is for the most part solid, I have concerns regarding the execution, interpretation of certain results and the conclusions drawn. Additionally, references to previous work are often incorrect or incomplete; I provide several examples below along with non-exhaustive suggestions for improvement. Finally, the manuscript would benefit from careful text revision and improvements to figure presentation, for which I also offer non-exhaustive guidelines.

Overall, I would recommend this manuscript undergo revision before its publication and I would be happy to reassess a revised version that addresses the comments above.

Reviewer 2

Evidence, reproducibility and clarity

Summary: The authors investigate the role of neurotrophins in development of the Drosophila visual system, where multiple waves of cell death eliminate populations of neurons. Using transgenic reporter lines, the authors examine expression of four spz genes, which encode ligands and four Toll receptor genes in the optic lobe. The authors argue that two of these ligands, spz-3/DNT-3 and spz-5/DNT-2, are expressed in the optic lobe concurrently with a wave of cell death. Through gain- and loss of function studies they argue that DNT-3 promotes survival through Toll-8 whilst DNT-2 promotes survival through novel interactions with Toll-2 (with a focus on the lamina neurons). Finally, the authors argue that DNT-2 and Toll-2 regulate L1 neuron targeting and size.

The involvement of neurotrophins in neuronal apoptosis has not been explored in detail in the visual system, making this a potentially interesting topic. However, there are several issues with the manuscript: The primary concern is that most of the authors' conclusions are not supported by the data presented. Many of the figures are difficult to interpret without the addition of appropriate markers. In addition, there are several inconsistencies both within the manuscript and within the context of existing literature that are not addressed. For example, the authors have not demonstrated that lamina neurons undergo apoptosis. This point is critical, as apoptosis, particularly that likely to be affected by neurotrophic factors, fine-tunes multicolumnar neuron numbers after they establish connections with their partners. On the other hand, unicolumnar neurons such as those in the lamina are not thought to need much fine-tuning because of their unicolumnar status. This distinction is not addressed in the manuscript. At present, the evidence

© 2026. Published by The Company of Biologists under the terms of the Creative Commons Attribution License (https://creativecommons.org/licenses/by/4.0/).

presented does not convincingly support the authors' conclusions, and substantial further work is needed before the claims can be firmly established. Overall, these limit the credibility and significance of this study.
Major comments

In general, the conclusions drawn about neuropil regions and specific cell identities are not supported by sufficient evidence. Across the figures, the identity of neurons and neuropils remains unclear due to the absence of subtype-specific markers or neuropil labelling that would place the data in the anatomical context of the optic lobe.

(1) The authors claim that spz and Toll genes are differentially expressed in the optic lobe throughout pupal development. However, the data presented do not convincingly support this conclusion. The primary concern is that the Gal4 reporter insertions used to infer gene expression do not match published single-cell transcriptomic datasets (as noted by the authors). This discrepancy calls into question the accuracy of these reporters (or the transcriptomics) and raises uncertainty about which dataset can be trusted. Given that these data are inconsistent, gene expression should be assessed directly by HCR to draw reliable conclusions.

Related comments: The authors claim that spz and Toll genes are expressed differentially in the optic lobe throughout pupal development. However, the characterisation of these expression patterns is lacking in the following aspects: - Time courses are inconsistent and incomplete across the different genes. - The conclusion that DNT-2/3 are expressed concurrently with cell death lacks evidence is not supported: DNT2/3 expression does not appear restricted to a clear time window and without co-staining for apoptotic markers this conclusion cannot be substantiated. - There is insufficient evidence to support the claim that spz-2 is specifically expressed in Mi1 neurons or that Toll-8 is specific to L4/L2. These claims are largely based on the transcriptomics data, which were inconsistent with the reporters in many other contexts. There are no neuron type markers costained to validate this claim. - Sample sizes are too low for some reporter lines (~2-4)

(2) The authors quantified cell death using Dcp-1 signal volume and concluded that overexpression of DNT-2 or DNT-3 reduces cell death, while loss of either increases cell death in the lamina. However, the lamina is not labelled with an appropriate marker, nor are neuropils labelled to make the structure recognisable making it unclear whether this wave of cell death is truly occurring in the lamina. This is a recurring issue throughout the manuscript. Related comments: the reported values for Dcp-1 signal volume differ dramatically between the Gal4 (~40,000) and mutant (~2,000) controls, without an explanation for this discrepancy.

(3) The claim that DNT-2 and DNT-3 promote survival via Toll-2 and Toll-8, respectively, is not sufficiently supported. The authors do not show that manipulation of each ligand specifically affects neurons expressing the relevant receptor. To substantiate this conclusion, the specificity of ligand manipulation should be tested. For example, through compensation experiments or by demonstrating that DNT-2 loss of function specifically induces cell death in Toll-2+ neurons.

(4) The authors further investigate a potential interaction between DNT-2 and Toll-2, concluding that DNT-2 promotes survival in the lamina, medulla, and lobula complex through Toll-2. However, no evidence is presented showing altered levels of apoptosis in the medulla or lobula/lobula plate following DNT-2 manipulation. The conclusion that DNT-2 promotes survival via Toll-2 is based on a Toll-2RNAi;DNT-2FL epistasis experiment, but this interpretation does not account for the reduced expression of each construct due to Gal4 titration. This issue also applies to subsequent experiments (Figs. 5 and 6). A Gal4 titration control is required for each of these experiments to exclude this confound.

(5) The authors conclude that DNT-2 production in Mi1 medulla neurons is required for L1 connectivity, survival, and morphology. However, these conclusions are based on manipulating DNT-2 and Toll-2 expression in L1 neurons, which does not directly test the requirement for DNT-2 in Mi1 neurons. In addition, the image quality in Figure 5 is problematic: brightness and contrast differ visibly between panels, making them

© 2026. Published by The Company of Biologists under the terms of the Creative Commons Attribution License (https://creativecommons.org/licenses/by/4.0/).

challenging to interpret. Finally, the discussion states that "loss of function for DNT-3 (spz-3) causes lamina cell death that is not naturally compensated for by DNT-2," but no evidence is presented in this manuscript to support that conclusion.

Other general comments

- Lack of labels for each individual image makes figures and text difficult to interpret/reference. - Gene names not italicised in figures - Inconsistent aspect ratio for graph axes (Obvious in figure 5C,D) - Image borders often not completely vertical/horizontal making them appear jagged in some figures e.g. Figure1A. - General typos e.g. (n=10 10 brains), inhibitor pf apoptosis p35 - Lobula complex should not be used to refer to lobula plate and lobula plug at this stage as they are distinct neuropils.

Figure 1:

- Arrows not explained

- N-Cadherin absent from some images without explanation

- First two images of 1B are identical.

- Images in panel B at different timepoints and spz-4 is missing without explanation.
Figure 3:

- Dark spot in DNT-3FL 3A image.

- Descriptions in text often lack specific details "Dcp1+ apoptosis in the lamina and outside the lamina too." Supplementary: -

Figures S2-S6 lack labels denoting the timepoint of each figure

- Figure S7 is illegible

- Supplementary figure 8 is referred to as S9 in text and is missing genotype information as well as neuropil labels on images

Significance

This is an interesting topic of investigation, and the findings-if substantiatedwould be of interest to the field of Drosophila visual system development and programmed cell death. In particular, it would be valuable to place these results in the broader context of programmed cell death as a mechanism for eliminating excess multicolumnar neurons during visual system development. However, at present the evidence presented does not convincingly support the authors' conclusions, and substantial further work is needed before the claims can be firmly established.
Expertise: Drosophila neurogenesis, neuron specification, cell signalling

Reviewer 3

Evidence, reproducibility and clarity

Summary

Building a functional nervous system with the correct number of correctly specified and wired pre- and post-synaptic neurons requires a tight control of proliferation and cell survival during development. Focusing on the latter, Naser Alshami and colleagues in the laboratory of Alicia Hidalgo investigated the role of three neurotrophins and their respective Toll receptors in regulating neuronal survival in the visual system of Drosophila. A combination of expression and genetic loss-of-function and gain-of-function analyses revealed a novel role for the neurotrophin

© 2026. Published by The Company of Biologists under the terms of the Creative Commons Attribution License (https://creativecommons.org/licenses/by/4.0/).

DNT2 (spaetzle-5) in controlling survival, dendritic spine formation, columnar axonal innervation of lamina neuron L1 expressing the receptor Toll-2.

Major Comments

- DNT-2 (spz-5) is described to be expressed in non-neuronal cells in the retina. However, the clustered arrangement of cells in cross-sections of the retina during pupal development (72h DNT-2 bottom panel) suggests that photoreceptors could be labeled by the reporter as well. This should be assessed by co-labeling experiments with Elav and/or mAb24B10. This is important, as photoreceptors may represent a presynaptic additional source of this neurotrophin to lamina neurons L1 (see below).

The most right hand panel of Figure 2A, first row, Toll-2, does not resemble a 24h APF optic lobe, and would need to be replaced by a correctly staged younger sample. This is relevant, as the image at 48 h APF shows extensive staining in a cluster at the medulla edge, suggesting that expression in medulla neurons may be more abundant at younger stages. The expression of Toll-2 in L3 neurons is not unambiguously demonstrated at the angle shown in Figure 1E first panel. Normally, MCFO clones should be able to reveal single cells instead of L1 and L3 together.

- Related to this part of the study, the authors describe that Toll-2 is expressed by connecting neurons (L1, Mi1, Tm3...). This is solely based on RNAseq data. In the next paragraph, the authors describe that Toll-2 is expressed in L1 and DNT-2 in Mi1 (or related) medulla neurons. However, this is rather vague, as it is not clear what "related" medulla neurons refers to. As expression in Mi1 neurons is at the heart of this study, it would be essential to verify that Mi1 is included in the Toll-2-Gal4 and DNT-2 expression pattern. Indeed, in Figure 1A (right panel, row 2, 48 h APF) expression of DNT-2 in this neuron subtype may be visible. This should be verified by markers and single cell labeling such as MCFO. Finally, the authors mention that the DNT-2 ligand could "reach" the Toll-2 receptor. Here some more precision would be helpful, as it is not clear, how far neurotrophins would need to travel, and synaptic contacts may form too late for mediating survival. Furthermore, there seems to be also the possibility that there is a substantial contribution of autocrine signaling, if L1 neurons indeed express both the ligand and the receptor, which would need to be presented more clearly.

- The next set of experiments uses over-expression of DNT-2 and DNT-3 in Toll-8 expressing neurons and assessment of survival rates in lamina neurons. Survival is determined by evaluating Dcp-1 signals rather than cell numbers. During normal larval development, 7 future lamina neurons per column are formed, two of which are eliminated by apoptosis. Thus, it would be important to verify whether it is these neurons that are surviving, using for instance Elav labeling; moreover lamina neuron specific markers (see Xu et al., 2024) may enable assessment whether the surviving neurons adopt specific lamina neuron identities in excess to the normal 5. It may also need to be considered that both neurotrophins could affect proliferation, if the numbers do not add up (as drivers may be active already during the third instar larval stage, this is possible).

- Gain-of-function experiments are followed by loss-of-function experiments, assessing the impact of loss of DNTs in entire animal mutants on lamina neuron survival. As for the gain-offunction studies, it would be important to know, which lamina neuron subtypes are dying in the absence of DNT-2 and DNT-3. Indeed, Figure 4C reveals a substantial loss of L1 and L3 neurons. The over-expression experiments of the ligands using the Toll-2 driver are complicating matters, considering that the driver line is described to be a mutant in Toll-2, and the ligand is expressed in the same neuron, creating an autocrine signaling situation. Moreover, since Toll-2-Gal4 is used to drive expression in all lamina neurons L1 and L3, and entire optic lobes are assessed, the numbers should be around 1600 in wild type, but are not, suggesting that there might have been some cell death in controls. If there are more lamina neurons in the over-expression situation, it would mean that additional neurons are adopting L1 and L3 identity and thus reveal an additional direct role in cell fate determination by DNT-2 and Toll-2 or an indirect role considering the intricate interactions of lamina neurons to adopt specific identities via N signaling (see Xu et al. 2024). This would need to be assessed more in detail using for instance independent drivers or markers for lamina neuron subtypes.

© 2026. Published by The Company of Biologists under the terms of the Creative Commons Attribution License (https://creativecommons.org/licenses/by/4.0/).

- The statement, that the remaining signal is coming from macrophages would need to supported by additional markers or described more carefully (as it could be glia). - Page. 9. The study reports a new interaction between DNT-2 and Toll-2 as its possible receptor. This is in part based on whole animal lethality, without providing quantification. This should be added.

- Interestingly, knock-down of Toll-2 in all neurons led to increased cell death of lamina neurons, which cannot be overcome by over-expression of DNT-2 [FL]. It would be interesting to assess whether cell death is even higher by providing a statistical test comparing the knock-down of Toll-2 with the knock-down of Toll-2 and the simultanous overexpression of DNT-2 in Figure 4B.

- Page 11 (also page 4). The authors describe that "connectivity of lamina neurons to medulla neurons takes place at 30-48 h APF and between medulla and lobula complex at 60-70h APF, and that expression of synaptic markers starts at 24h APF". It is not clear how the authors define connectivity (here and throughout the manuscript). While axonal and dendritic projections are established during the first half of pupal development, functional synapses are thought to be solely established from mid-pupal development onwards (early expression of synaptic markers may not be indicative of synapse formation as early as 24h APF). Moreover, there is no solid evidence yet that connections form sequentially between the lamina and medulla, and medulla and lobula and lobula plate neurons. This description would need to be adjusted. A reference for the occurrence of spontaneous activity (prior to synapse formation) should be provided: Akin et al. 2019. This is important for the subsequent interpretation that cell death overlaps with "connectivity": if this term is used to refer to synapse formation, this may not hold, and need adjustment

- Next, the study assesses the impact of loss of Toll-2 in surviving lamina neurons L1 on columnar axonal branching. The authors observed that loss of Toll-2 or over-expression of DNT-2 leads to extension of axonal branches into neighboring columns in the medulla neuropil layer M1. Here, it would be important to assess, whether this phenotype is really due to the loss of Toll-2 or the fact that neighboring L1 neurons are missing (even just one neighbor missing may be enough to create this defect as contact-dependent repulsion may no longer work, Millard et al. 2007). Toll-2 MARCM clones would be able to address this point. Without further experiments conclusions need adjustments. Finally, the authors conclude that this is an indication for a role in connectivity, but indeed, it is not clear whether these projections lead to abnormal connections, as the thin projections may not form synapses. This statement would need to be adjusted or supported by including synaptic markers, such as Brp/or TransTango experiments.

- The study concludes with the statement that over-expression of DNT-2 FL and knock-down of Toll-2 alters dendritic spine formation of lamina neurons L1 in the lamina, it increases by over-expression and decreases in Toll-2 knock-down. This is in contrast to the axonal projections, where additional extensions are observed in both genotypes. This should be discussed, as the opposite effects may be in line with an indirect effect through loss of neighbors in medulla columns (in contrast to the lamina).

- The authors conclude in the discussion (page 15 and 17) that DNT-2 by medulla neurons Mi1 or related medulla neurons is key to promoting survival of lamina neurons L1, expressing the Toll-2 receptor. However, the manuscript does not provide any direct evidence that it is indeed Mi1 (or other medulla neurons) playing this role. Considering that DNT-2 may also be provided by photoreceptors (see above), this statement may need support by additional experiments, such as a Mi1 specific or photoreceptor-specific overexpression of DNT-2 in a DNT-2 whole animal mutant background and assessment of survival of labeled L1 neurons (e.g. using the markers Svp/Zfh1 (Xu et al. 2024). Moreover, it would be crucial to indeed determine the time point of when cells are undergoing apoptosis and to assess whether this would coincide with synaptogenesis. Such experiments would allow to comment on a potential retrograde signaling process between connecting neurons.

- The data and molecular and genetic methods are presented in detail in the Material and Method sections, as well as through pertinent supplementary tables. Similarly, the statistical analyses and sample numbers are indicated in the Material and Methods section and related supplementary tables. Table S4 does not indicate genotypes, but short versions of crosses, which should be corrected. Moreover, the list of fly lines seems to miss one line (UASmCherry). Concerning the antibody list, sources are not provided (unlike stated in the main text). In the Result section,

© 2026. Published by The Company of Biologists under the terms of the Creative Commons Attribution License (https://creativecommons.org/licenses/by/4.0/).

differences are described simply as statistically significant decreases and increases, however, at times it would be useful to add some indication about the level of differences (percentage or fold times).

Minor comments

- The figures are informative, however, their orientations are not as indicated horizontal or lateral, but often oblique and not consistent (e.g. Fig 1a, last four panels). This makes comparisons more difficult. The authors could possibly find more suitable optical sections and present images more consistently. - Please provide the correct reference describing the occurrence of spontaneous activity (Akin et al. 2019). - The manuscript would be more easily accessible to non-experts, if schematic drawings would be provided (neuron subtypes, model). - The final paragraph of the discussion about DNT-2 keeping connecting neurons together is not easily understandable, as the term "keeping together" remains undefined.

Significance

As in the vertebrate nervous system, insect nervous systems rely on neurotrophins to control the survival and correct wiring of neurons. This study proposes a model, that advances our understanding as to how a specific neurotrophin, delivered by a post-synaptic neuron, acting via a specific receptor, controls several developmental steps of one of its pre-synaptic partners. The study assessed the expression patterns of three neurotrophins and the Toll receptor family, using the T2A-Gal4 expression system, revealing their dynamic expression in photoreceptors, specific lamina and medulla neurons, as well as tracheae and glia during pupal development. A strength of the study represents the identification of DNT-2 and Toll-2 as interacting partners, which could be assessed by the extensive set of specifically generated reagents for this study (Gal4 drivers and mutants). However, a number of statements may require additional experiments to conclusively support the proposed model, in particular the action of DNT-2 and Toll-2 in Mi1 and L1, respectively. Moreover, it would be important to critically assess the timing of events of cell death, setting up of projection patterns and synapse formation.

Audience

The outlined findings, if strengthened further, will be of interest for scientists studying nervous system development in vertebrates and invertebrates in general, and the action of neurotrophins in particular.

Author response to reviewers' comments

Manuscript number: RC-2025-03128

Corresponding author(s): Alicia Hidalgo

[Please use this template only if the submitted manuscript should be considered by the affiliate journal as a full revision in response to the points raised by the reviewers.

1. General Statements [optional]

We would like to thank all three Reviewers for their overall positive comments and constructive criticisms that have helped improved our manuscript. We are delighted that **Reviewer 1** considered that we "provide compelling evidence that different DNTs and their Toll receptors are expressed in optic lobe neurons, and their activity regulates neuronal survival during optic lobe development", for considering the data "mostly solid" and for their overall positive recommendation for publication following revision. We are very pleased that **Reviewer 2** considered the topic of neurotrophin function in the visual system "an interesting topic of investigation, and the findings-if substantiated-would be of interest to the field of Drosophila visual system development and programmed cell death. In particular, it would be valuable to place these results in the broader context of programmed cell death as a mechanism for

© 2026. Published by The Company of Biologists under the terms of the Creative Commons Attribution License (https://creativecommons.org/licenses/by/4.0/).

eliminating excess multicolumnar neurons during visual system development". We are pleased that **Reviewer 3** considered our identification of DNT-2 and Toll-2 as ligand-receptor partners as a "key strength", and for commenting that "the …study… advances our understanding as to how a specific neurotrophin, delivered by a post-synaptic neuron, acting via a specific receptor, controls several developmental steps of one of its pre-synaptic partners" which would be of interest to "scientists studying nervous system development in vertebrates and invertebrates in general, and the action of neurotrophins in particular".

2. Point-by-point description of the revisions

We have addressed all the criticisms raised by the Reviewers, as best we could. The revision has required the addition of two further authors.

For this revision, **we provide new data in:**

- New Figure 3: identification of DNT-2 and Toll-2 cells
- New Figure 4: GRASP connections between DNT-2 and Toll-2 neurons
- New Figure 5A: showing *DNT-2* expression concurrently with Dcp1+ apoptosis
- New Figure 6G,H: showing that excess Toll-2+ cells upon DNT-2 over-expression lack L1 and L3 markers
- New Figure 7A-C: showing that lamina neurons are input cells of DNT-2+ neurons and that *DNT-2* function is required for the connection between DNT-2+ and lamina neurons.

We have also made changes to the figures, text, references and presentation, following the Reviewers' recommendations. We very much hope the Reviewers will be satisfied with these improvements.

Reviewer #1 (Evidence, reproducibility and clarity (Required)):

SUMMARY

Alshamsi et al. investigate the role of Drosophila neurotrophins (DNTs) and their Toll receptors in regulating neuronal apoptosis during optic lobe development.

The authors provide compelling evidence that different DNTs and their Toll receptors are expressed in optic lobe neurons, and their activity regulates neuronal survival during optic lobe development. They further show that disruption of DNT/Toll signaling impacts neuronal morphologies.

COMMENTS

ABSTRACT

"Over-expression of DNT-3 (spz-3) and DNT-2 (spz-5) could rescue natural occurring cell death, whereas their loss of function caused cell death, showing that DNT-3 and DNT-2 can, and are required to, promote cell survival during optic lobe development."

I find it more appropriate to say that OE prevents naturally occurring cell death because it inhibits a normal physiological process."rescue" would only be correct if there were an experimental or genetic loss (e.g., deletion of a survival factor) and you are restoring normal survival levels

Done.

"Importantly, DNT-2 is expressed in Mi1 neurons and Toll-2 in connecting L1 neurons. We show that DNT-2 functions in concert with Toll-2, as Toll-2 RNAi knock-down prevented the rescue of apoptosis by DNT-2 over-expression and all Toll-2+ neurons were lost in DNT-2 mutants."

I find this sentence very difficult to follow

We have rewritten this sentence, we hope it is now clearer.

© 2026. Published by The Company of Biologists under the terms of the Creative Commons Attribution License (https://creativecommons.org/licenses/by/4.0/).

I suggest moving "Importantly, DNT-2 is expressed
in Mi1 neurons and Toll-2 in connecting L1
neurons." to the next sentence.
Thank you, this has been edited.

"by specifically investigating the Mi1 (DNT-2+) and L1 (Toll-2) synaptic partners", alterations in DNT-2 or Toll-2 expression levels impaired connectivity of L1 neurons at the M1 medulla layer and altered dendritic morphology of L1 neurons
"As DNT-3 (spz-3) and DNT-2 (spz-5) are expressed in the medulla and they could influence both lamina and medulla neurons, this suggests that their function maintaining cell survival could enable the stabilisation or alignment of connected neurons across medulla columns."

influence what? this is very vague and needs a temporal understanding of when neurons die, synapses are formed, and consider the phenotypes of the mutants and RNAi experiments.
This has now been reworded.

INTRODUCTION

"Neuronal survival is maintained by neurotrophic factors secreted in limited amounts by target cells, leading to the survival of only those neurons that receive trophic support (Levi-Montalcini, 1987, Davies, 2003)."

Perhaps the authors can be more precise, e.g. One mechanism by which Neuronal survival is regulated is through neurotrophic factors secreted in limited amounts by to be synaptic partners and adjacent cells
This has been edited.

"In this context, if neurotrophism is fundamental for nervous system development, it could have been enabled by evolutionarily conserved molecular mechanisms."

I think the authors want to suggest that given the fundamental and widespread role of neurotrophism in nervous system development, it remains unknown if it relies on evolutionarily conserved molecular players.
This has been edited.

"Neurotrophins - NGF, BDNF, NT3, NT4 - are the main growth factors maintaining neuronal survival in the vertebrate nervous system (Levi-Montalcini, 1987, Lu et al., 2005). Importantly, they can also promote cell death, depending on context (Lu et al., 2005). They can promote cell survival via their Trk receptors and ERK and AKT downstream and via p75NTR and NF¤B downstream, or cell death via p75NTR, Sortilin, and JNK signalling instead (Lu et al., 2005). mechanisms."

The authors should avoid the repeated use of "they".
Done.

"There are six spz and nine Toll paralogous genes in Drosophila, which could play distinct functions. In fact, at least full-length DNT-1 and Toll-1 can promote cell death instead, and at least Toll-6 can promote either cell survival or cell death, depending on context (Foldi et al., 2017, Singh et al., 2025, Zhu et al., 2008). Importantly, mature DNT-1 and DNT-2 with Toll-6 and Toll-7 are required for and can promote neuronal survival during circuit formation in the embryonic ventral nerve cord (McIlroy et al., 2013, Zhu et al., 2008)."

I find this paragraph difficult to follow. I suggest the following editing, which the authors might want to consider:

"There are six spz and nine Toll paralogous genes in Drosophila, which could play distinct functions. In fact, at least full-length DNT-1 and Toll-1 can promote cell death instead, while and at least Toll-6 can promote either cell survival or cell death, depending on context (Foldi et al., 2017, Singh et al., 2025, Zhu et al., 2008). Importantly, mature DNT-1 and DNT-2 with Toll-6 and Toll-7 are required necessary and sufficient to promote for and can promote neuronal survival during circuit formation in the embryonic ventral nerve cord (McIlroy et al., 2013, Zhu et al., 2008).

© 2026. Published by The Company of Biologists under the terms of the Creative Commons Attribution License (https://creativecommons.org/licenses/by/4.0/).

done

"During this time (24-50h APF), connectivity between photoreceptors, lamina and medulla neurons is established; this is followed by medulla neurons connecting to lobula neurons;"

I find this sentence misleading, if not incorrect. If by connectivity the authors mean synaptogenesis, for all that is known, synaptogenesis has been shown to occur from from mid-pupal development (P50) onwards
If by connectivity, the authors mean the targeting of specific neuropiles and layer organization, it is also incorrect that lamina and medulla organization precedes the connectivity between medulla and lobula neurons. These processes are all concurrent. Can the authors please clarify?
Thanks for the feedback. We have now modified this text.

"and by 72h APF cell death has greatly diminished and synaptogenesis completes connectivity patterns, in preparation for adult eclosion at 96h APF (Millard and Pecot, 2018, Melnattur and Lee, 2011, Hadjieconomou et al., 2011, Kurmangaliyev et al., 2020) "

Kurmangaliyev et al., 2020 is probably not an appropriate citation here as it mostly deals with transcriptional programs of circuit assembly in the developing optic lobe
Thanks, removed.

"Thus, 24-48h APF is a critical period to maintain necessary lamina and medulla neurons alive in the optic lobe."
Perhaps the authors want to revisit this sentence and explicitly say that 24-48h APF is a period where apoptosis defines cell numbers.
Thanks, done.

"The development of the Drosophila visual system has been well described (Holguera and Desplan, 2018, Melnattur and Lee, 2011, Millard and Pecot, 2018, Hadjieconomou et al., 2011, Behnia and Desplan, 2015)."

I find that Behnia and Desplan, 2015 is not appropriate, as it is a review that describes the characterization of neuronal circuits underlying visual modalities in the fly brain, and not their development. The following reviews dealing with different aspects of neurogenesis, neuropile development and circuit formation are likely more relevant:
Bakshi et al Current Opinion in Neurobiology 2025
Malin et al PNAS 2021
Ngo Dev Bio 2017
Thank you very much, very helpful.

"R7 and R8, together with lamina neurons target to medulla layers M6 and M3, respectively, organizing into medullar columns that respond to the same point in visual space and maintain retinotopy."
I find this sentence misleading because lamina neurons do not target M6 and only L3 targets M3.
Thank you, this has now been corrected.

"Medullar interneurons also form connections across multiple layers, where each layer represents different visual features (Fischbach and Hiesinger, 2008, Millard and Pecot, 2018)."

Here, Behnia and Desplan, 2015, Matsliah et al Nature 2024, Borst and Groschner, Annu. Rev. Neurosci. 2023, and even Schnaitmann et al J Comp Physiol A Neuroethol Sens Neural Behav Physiol 2020 are better references that review feature detection and circuit organization in the optic lobe.
Thank you very much, this is super-helpful. These have now been added.

"Neurons within the lobula complex integrate signals from the medulla and project to the optic glomeruli in the central brain and motor outputs to enable appropriate behavior (Behnia and Desplan, 2015, Borst et al., 2020, Courgeon and Desplan, 2019b)."

© 2026. Published by The Company of Biologists under the terms of the Creative Commons Attribution License (https://creativecommons.org/licenses/by/4.0/).

I find Courgeon and Desplan, 2019b is not very appropriate here, as it reviews the coordination of neural patterning in the Drosophila visual system. More adequate and relevant manuscripts and reviews are Wu et al. elife 2016, Tanaka and Clark, 2022, Lapoetke et al. Neuron 2022, even Zhao et al. elife 2024
Thank you, these have now been added.

"Spz-5 is well known as Drosophila neurotrophin-2 (DNT-2), and as Spz-3 has been proposed to have neurotrophin functions which we expand on and demonstrate here, we refer to Spz-3 as DNT-3 (Zhu et al., 2008, Coutinho-Budd et al., 2017, Sun et al., 2024, Ballard et al., 2014, Ulian-Benitez et al., 2017)."

This last paragraph of the introduction seems out of place, and partly redundant with page 3. Perhaps the authors would like to finish the introduction with a paragraph highlighting the major findings and conceptual advance of the manuscript? This seems to be a good and natural way of following their previous sentence "Here, we asked whether neurotrophin family ligands encoded by DNTs (spzs) and their Toll receptors could regulate cell survival during neural circuit formation, in the Drosophila pupal optic lobe."
Thank you, now done.

RESULTS

I suggest the following edit:
To ask whether DNTs (spzs) are expressed in the pupal optic lobe, we generated T2A-Gal4 driver lines for spz-1,3,4,5 fly lines, crossed them to 10xUASmyrGFP or 20UAS6xmCherry reporter flies, and analysed resulting progeny optic lobes during development and in the adult with anti-GFP antibodies, as required (Figure 1A).
Thank you, done.

Also, If the lines were also crossed with mcherry, mentioning anti-GFP antibodies is incomplete.
Thank you for spotting this mistake, now corrected.

"spz-1MI02318-T2A>myrGFP and spz-1MI02318-T2A>6xmCherry revealed expression in a few centrifugal neurons in the lobula complex that projected to the lamina,"
At which stage?
This has been edited.

"subsequently medulla neurons and abundant arborisations into the lobula complex and medulla."
Subsequent to what? I am sorry, but I don't understand this description.
This has now been changed.

"Expression from spz4MI5678 -T2A->myrGFP was not detected until 72h APF"
Data before 72h APF is missing
Where was it expressed? Which cell types? Which neuropiles?
it was not expressed, which is the reason we are not showing it, and we said this in the text: "….expression…. was not detected until 72h APF…".

"And then was found in the medulla and lobula complex and followed by the trachea." At which stage?
What was followed by the trachea? I think the authors mean that at later staged (in the adult) expression was restricted to the trachea in both medulla and lobula
No, we meant at 72h APF. I think using then in this context to refer to 72h APF is grammatically correct, but the text has been edited to "at this time-point" for clarity.

"spz-3-T2A>6xmCherry (hereby named DNT-3) was highly expressed in non-neuronal retinal cells and medulla neurons;"
At what stage?
This has been edited.

"And subsequently in the trachea and possibly glia."

© 2026. Published by The Company of Biologists under the terms of the Creative Commons Attribution License (https://creativecommons.org/licenses/by/4.0/).

The authors could and should explain how they reach this conclusion. Given that no cell type specific markers were used, this identification was likely based on morphological features.
Yes, the identification was based on morphology. The pattern is also highly distinct from that seen by neuronal drivers and typical neuronal projections in the optic lobe. This clarification has now been added.

"Finally, DNT-2-T2A>6xmCherry (spz-5) was found in medulla neurons, which could be tentatively identified as Mi1 medulla neurons (Nern et al., 2025) by 48h APF, and this pattern was maintained." The authors suggest these neurons are Mi1 based on what?
Also, Fischbach's Cell Tissue Res (1989) seminal paper is probably worth mentioning.
In the original submission, this was based on morphology, indeed by comparing to Fischbach's drawings
- which however we forgot to cite, and we have now added, thank you. For this revision, we provide new evidence that DNT-2>mCherry+ neurons colocalise with the Mi1 marker anti-Bsh, demonstrating that indeed they are Mi1 neurons. **These new data are given in new Figure 3A-C.**

"Abundant cells expressed spz-1 in the lobula complex (Figure 1B, left) and medulla (Figure 1B, right), and DNT-3 (spz-3) and DNT-2 (spz-5) in the medulla (Figure 1B) during optic lobe development. DNT-3 (spz-3) and DNT-2 (spz-5) were expressed in distinct non-neuronal cells in the retina, seen in Multi Colour Flip Out (MCFO) clones (Figure 1C)."
This sentence is misleading. These experiments allow the authors to conclude that spz+ neurons innervate these neuropiles. It does not allow the authors to conclude that spz molecules localize to the neuropiles.
The authors should revise these claims in the main text and relevant figure legends.
We respectfully disagree with the reviewer or perhaps there is a misunderstanding. The T2A-GAL4 with reporter expression reveal transcription and translation profiles for these genes in those neurons. That is, the cells visualised with the reporters are indeed the cells expressing each of the ligands.

If we understand the Reviewer's point, it presumably means that expression in a neuronal type as revealed by these membrane reporters does not reveal the point from which the ligands are secreted (i.e. axonal terminals, dendrites or somas), as reporters are distinct proteins from the secreted ligands. This is precisely the reason why we had shown in the original Figure 1B (and now relabelled Figure 1E-G) the location of the somas, to take into account the distribution of the whole neurons regardless of the ligand secretion site. We understand that we had not made this point clear before, and we have now edited it.

"To visualize the distribution of Tolls in the optic lobes during pupal development, we used the GAL4 lines previously described (Li et al., 2020), driving expression of the reporter myrGFP (Figure 2A)." Same comment as above
The same rationale as above does not quite apply here, as in Drosophila Toll receptors are all located at the plasma membrane. The Gal4 drivers used reveal gene expression patterns. As the Tolls are transmembrane receptors, they are more likely than the ligands to be distributed along the membrane in the patterns revealed by the reporters. Nevertheless, the Tolls could be restricted to certain neuronal compartments. We have edited the text to only use the term "expression" to refer to transcription (ie mRNA), and "distribution" to refer to protein location, as per convention.

"Using MCFO clones as well as myrGFP, we could identify some of the Toll-8+ neurons as Lawf1, feedback neurons projecting from the medulla to the lamina (Figure 2B), and L2 and L4 lamina neurons (Figure 2B, C); Toll-6+ cells to include L3 and L4 lamina neurons (Figure 2B,D); and Toll-2+ neurons as L1 lamina neurons which target to M1 and M5 medulla layers and L3 lamina neurons that project to M3 (Figure 3B,E) (Hakeda-Suzuki and Suzuki, 2014, Behnia et al., 2014)."
Hakeda-Suzuki and Suzuki, 2014, Behnia et al., 2014 are not the most appropriate references. Instead, I suggest the authors should cite Fischbach's Cell Tissue Res (1989).
Thank you very much, this has now been edited.

"Overall, the expression in the scRNAseq dataset (Kurmangaliyev et al., 2020) of the spz ligands and Toll- 8 (also known as Tollo) data were less consistent with the cell biology data, whereas the expression of Toll-1, -2 and -6 confirmed cells seen with the cell-biology based reporters"

© 2026. Published by The Company of Biologists under the terms of the Creative Commons Attribution License (https://creativecommons.org/licenses/by/4.0/).

It is perhaps more accurate to refer to the cell-biology based reporters as translation reporters, which is what T2a based Gal4 drivers are.
Good point, although as they depend on splicing, they are both transcriptional (ie expression) and translational. This reference to translation has now been added in multiple points, and most particularly for the ligand reporters, thank you. Regarding the Tolls, Toll-2Gal4, Toll-6Gal4 and Toll-8Gal4 do not carry T2A-Gal4, but Gal4, as explained in the manuscript.

"Most particularly, Toll-2 mRNA (synonym 18w) was found in L1 and L3 lamina neurons over time, plus also in L5 at 24h APF, and Toll-6 mRNA was found in L2, L3, L4 over time, plus also in L1 at 24h (Supplementary Figure 1-6)."
This sentence is difficult to read and could be considered poorly written for several reasons including:
- "plus also" is repetitive. "Plus" and "also" serve the same function.
- Inconsistent punctuation. The lack of commas before "and Toll-6 mRNA..." makes the sentence feel unbalanced.
-Vague time reference. "Over time" is imprecise. It's unclear whether it means during development, at multiple timepoints, or something else.
Also regarding scRNAseq analysis:

- the authors mention "We compared our reporter-based profiles with published scRNAseq datasets of the optic lobe through development (Kurmangaliyev et al., 2020, Ozel et al., 2021)" however the Ozel dataset doesn't seem to be used.

Thank you, this has now been improved and reference removed.

Also, from the Material and Methods section:

"The data were imported as a Seurat object, and cells corresponding to specific timepoints (e.g., 24 h, 36 h) were subsetted based on the provided metadata. Dimensionality reduction was carried out using principal component analysis (PCA), followed by Uniform Manifold Approximation and Projection (UMAP) embedding computed on the first 30 principal components. Cluster annotations provided by the original authors were used for all cluster-level analyses and visualisations."

The Kurmangaliyev dataset is already processed. I am probably missing something here, but is not obvious to me why the authors performed PCA again.
I see. I engaged a further author (Anna Lassotta, who had experience in this kind of analysis) to carry out this analysis, as I am not an expert, and this is how they approached it, which seemed reasonable to me.

"To conclude, at the time of naturally occurring cell death (0-48h APF), Toll-1 is highly expressed throughout the optic lobe; Toll-2, -6 and -8 are expressed in the medulla; Toll-8 and Toll-6 are prominently expressed in the lobula complex, and Toll-6 and Toll-2 are prominent in the lamina."
This is misleading. These experiments allow the authors to conclude that toll+ neurons innervate these neuropiles. It does not allow us to conclude that toll molecules localize to the neuropiles. The authors should revise these claims in the main text and relevant figure legends
I'm afraid this is a misunderstanding. We used italics, which is the conventional font to refer to genes (eg *spz*, *Toll*); instead "regular font and capitalised name" (eg Spz, Toll), is the conventional way to refer to proteins, which in this paragraph we were careful to avoid. Thus, in this paragraph we refer to gene expression. It is correct to say that the *Tolls* are expressed in the neurons described.

The Reviewer is correct in saying that gene expression does not reveal where the actual proteins are located (ie in dendrite, axon, soma), but we did not make any claim on the sub-cellular distribution of the Toll receptors. Thus, no change is required in the text, figures or legends where we refer to gene expression. Still, we have made some minor edits for clarity.

"To ask whether DNTs can promote cell survival in developing optic lobes, we over-expressed DNT-2 (spz-5) and DNT-3 (spz-3) and visualized dying cells with the apoptotic marker anti-Dcp1 at the peak of naturally occurring cell death (24h APF)."
These experiments were done using Toll8-Gal4 and nsyb-Gal4 drivers.
What's nsyb-Gal4 expression during development? Is the expression of this driver consistent with the conclusions drawn from these experiments?

© 2026. Published by The Company of Biologists under the terms of the Creative Commons Attribution License (https://creativecommons.org/licenses/by/4.0/).

nsybGal4 is the most commonly, widely and preferred used pan-neuronal Gal4 driver by the Drosophila neuroscience community to drive expression in all neurons, and exclusively in neurons.

Once upon a time elavGAL4 used to be the common pan-neuronal driver, but following the discovery that it is also transiently expressed in glial cells and neuroblasts during metamorphosis, its use as a pan-neuronal driver was virtually discontinued and substituted by nsybGAL4. Nsyb-Gal4 drives Gal4 expression downstream of the regulatory region of neuronal-synaptobrevin gene. Synaptobrevin forms part of the SNARE complex, which controls synaptic vesicle fusion for neurotransmitter release.

"To test whether DNT-2 could promote cell survival during optic lobe development, we over-expressed full-length DNT-2FL or cleaved DNT-2CK in all neurons with nsybGAL4. This reduced the incidence of Dcp1+ apoptosis in the lamina and outside the lamina too (Figure 3C-D and Supplementary Figure S9C,D)."
How do the authors explain that DNT-2CK reduced the number of Dcp1+ cells?
As explained in page 11, the effect of DNT-2CK is cell autonomous. This means that cells producing DNT- 2CK can also respond to it. This is possible because medulla neurons also express at least Toll-2. The interaction between the DNT-2CK ligand and the Toll-2 receptor could occur at the plasma membrane or intracellularly, before Toll-2 reaches the plasma membrane. Previous published reports have shown that cleaved forms of spz-1 (also called activated Spz and SpzC06), spz-2/DNT-1 (called DNT-1CK and DNT- 1CTD), and spz-5/DNT-2 (called DNT-2CK) all are functional in vivo, reproduce gain of function phenotypes and can rescue the respective null mutants (Foldi et al., 2017 JCB; Sun et al., 2024 eLife; Zhu et al., 2008 PLoS Biology; Ulian-Benitez et al. 2017 PLoS Genetics; Ligoxighakis et al 2002 Science).

"We generated DNT-3 (spz-3) loss of function mutants by P-element mobilization. DNT-2 and DNT-3 loss of function mutants caused considerable cell debris in the medulla and lobula complex, which compromised the analysis in this region, so we focused on the lamina."
Perhaps, rather than stating that the mutants caused considerable cell debris, the authors could say that the mutants displayed considerable cell debris.
Done.

More importantly, I have concerns with the data from these experiments (Figure 3). Dcp1 signal volume intensity using Imaris. In all panels (A,C,E) the segmented images do not match the raw DCP1 staining, raising concerns on how much can one rely on this quantification.
Could this be because the Dcp1 staining shown is a single z plane and the segmentation is a 3d rendering? The authors should carefully and robustly explain this discrepancy which is present in all images where Dcp1 signal volume intensity was quantified.
Yes, the microscopy images on the left show projections of only a few sections from the raw image stacks that comprise each over 100 sections. The Imaris images on the right show the entire rendered volume of the total Dcp1 signal. Projections of a full stack of over 100 images result in images of very poor resolution that do not reflect the data. For this reason, to show what the raw data look like we show thin optical projections, but these do not reflect the extent of Dcp1 signal. This is the reason we show both image types.
We have added an explanation in the manuscript text.

Also, could the authors explain why the quantifications in Figure 3B and 3C differ by an order of magnitude (10×) from those in panel 3D? Please look at the WT control, there is a 10X difference in signal volume intensity.
The experiments were carried out in blocks (ie a loss of function block, an over-expression block), at different periods in time. This resulted in different laser age, confocal settings, staining quality, image processing and analysis with Imaris, amongst other variables. The expertise of the PhD student carrying out the experiments also changed, as his skills improved over time. Dcp1 experiments are difficult as they require accurate staging, careful fixations and staining, and are extremely time consuming. Most importantly, each experimental block had internal consistency, with the values obtained from the controls not differing exorbitantly from those of the mutants or over-expressing lines. Importantly too, quantification of Dcp1 volumes was done automatically with Imaris, and it was not possible to anticipate the result until the analysis was completed. Thus, the procedure and analysis was unbiased.

© 2026. Published by The Company of Biologists under the terms of the Creative Commons Attribution License (https://creativecommons.org/licenses/by/4.0/).

"Toll-2pTVGAL4 flies are heterozygous mutant for Toll-2, and, remarkably, in combination with DNT-2 homozygous mutants resulted in semi-lethality, revealing a functional interaction between these two genes.
I cannot entirely follow this conclusion.
I understand the authors propose that the combination of partial loss of Toll-2 and full loss of DNT-2 affects viability, more than either mutation alone.
Is this what they mean? Can the authors comment on the viability of DNT-2 mutants?
Trans-heterozygous $DNT\text{-}2^{18}/DNT\text{-}2^{37}$ mutants are fully viable, which we have published before (e.g. Ulian-Benitez et al 2017 PLoS Genetics; Sun et al 2024 eLife). Heterozygous $Toll\text{-}2^{pTV\text{-}Gal4}$ mutants are fully viable, which we have published before (Li et al 2020 eLife). Lethality only occurs when the two are combined.
We have clarified this in the text.

"Macrophages loaded with HisYFP and distributed mostly between the retina and lamina could be observed across these samples (Figure 4C), suggesting they had engulfed dead cells" How do the authors identify these YFP+ cells as macrophages?
We suggested they were macrophages based on morphology, large size and their un-patterned, erratic locations. Unfortunately, we were unable to verify that these are indeed macrophages. We tried, using an anti-croquemort antibody, but the staining was not satisfactory. Since we were unable to demonstrate they were macrophages, in response to this Reviewer, **we have now removed the claim and added a speculation instead.**

"Together, these data show that DNT-2 functions as a ligand for Toll-2 to maintain the survival of neurons in the lamina, medulla and lobula complex during optic lobe development."
While the results from Figure 4 showing that DNT-2 acts as a ligand for Toll-2 to support neuron survival are solid (in particular panels C-F), it doesn't necessarily mean all neurons die directly due to loss of Toll- 2 signaling.
It is plausible that Neurons that express Toll-2 die because they lose critical survival signals. The death of these Toll-2-expressing neurons could then cause a cascade effect, where neighboring or connected neurons die indirectly due to loss of trophic support, disrupted circuits, or secondary damage.
So, the observed cell death in multiple regions may be a combination of direct effects on Toll-2-positive neurons and indirect effects on other neurons.
DNT-2 is a neurotrophin-family ligand, and neurotrophins maintain neuronal survival. We have published before that Tolls maintain neuronal survival via NFkB and ERK signalling downstream (see Zhu et al 2008 PLoS Biology; McIlory et al 2013 Nature Neuroscience; Foldi et al 2017 J Cell Biol). Thus, indeed, Toll-2+ neurons die in the absence of Toll-2 because they lack the neurotrophin-induced signalling that would maintain their survival.

It is conceivable that dying cells could have non-autonomous secondary effects on other neurons, causing more widespread death. Whether this takes place or not, the data show that in the absence of DNT-2 in a Toll-2 heterozygous background, all Toll-2+ cells die, and this is the important point. These data strongly support that DNT-2 is a ligand for Toll-2. **We have added a comment in the text.**

"In the Drosophila pupa, connectivity of lamina to medulla neurons takes place at 30-48h APF, and between medulla and lobula complex at 60-70h APF (Kurmangaliyev et al., 2020, Millard and Pecot, 2018, Pecot et al., 2014, Hadjieconomou et al., 2011)."
I have the same comment as mentioned above regarding the timing of connectivity. If by connectivity, the authors mean the targeting of specific neuropiles and layer organization, it is incorrect that lamina and medulla organization precedes the connectivity between medulla and lobula neurons. These processes happen concurrently. Can the authors please clarify?
Thank you for this feedback on the timing, we have now corrected the errors. By “targeting and connectivity” we refer to the developmental context when during axon guidance, the growth cone identifies the target region or cell, slows down, and stops at the target – regardless of whether synaptogenesis occurs at the same time or not. I have now used the term “synaptogenesis” and “synaptic connectivity” to refer to the formation of synaptic contacts. **The text has now been improved.**

© 2026. Published by The Company of Biologists under the terms of the Creative Commons Attribution License (https://creativecommons.org/licenses/by/4.0/).

"Importantly, the expression of synaptic markers starts at 24h, peaks at 60h APF and spontaneous neuronal activity takes place at 48h APF, meaning that at least some neural circuits are already connected by this point (Kurmangaliyev et al., 2020)."
The correct placement of the reference to Kurmangaliyev et al. is after "peaks at 60h APF " A reference to Akin et al and Bajar et al when referring to PSINA is missing.
Thank you for this feedback, now added. Throughout the manuscript, we have generally given the references at the end of the sentences, regardless of where eactly the information was used within the sentence. In this case, we have inserted the reference as requested.

"Thus, the period of naturally occurring cell death overlaps with connectivity"
I think the authors mean that the period of cell death is concurrent with the development of synaptic connectivity.
Indeed, thank you. The text has been edited.

"L1 neurons normally project along columns that can be labelled with mAb24B10, and target to layers M1 and M5 of the medulla."
The authors should mention that 24b10 labels the photoreceptors, providing a spatial reference to identify medulla columns
Thank you. The text has been edited.

"Interestingly, Toll-2RNAi knock-down did not alter the phenotype caused by DNT- 112FL overexpression, and impaired targeting to the same extent as each genetic manipulation alone (Figures 5B,D)."
How do the authors interpret these results? And perhaps the authors would like to explain the rationale of overexpressing DNT-2fl in L1 neurons, that do endogenously express it.
Unfortunately, we do not have DNT-2LexA flies, we tried twice making them, and it didn't work. L1 neurons do not normally express DNT-2. The rationale for over-expressing DNT-2FL in L1 neurons is to activate Toll-2 in these neurons. We could not over-express or knock-down DNT-2 in medulla neurons, because we were using an L1 split GAL4 driver. Similarly, using this L1 split GAL4 prevented us from using the DNT-2 mutant background. Since DNT-2FL is a secreted protein, over-expressed DNT-2 from L1 neurons should also be received autocrinely by L1 neurons.

The fact that both loss of function for the receptor and gain of function for the ligand give the same phenotype can be explained in two ways: (1) that the phenotypes are an indirect consequence of altering the normal cellular organisation. (2) That excess or deficit for ligand or receptor disrupt targeting, as is common with neurotrophin signalling both in Drosophila and in mammals (see Zhu et al 2008 PLoS Biology; McIlroy et al 2013 Nature Neuroscience, where we reported similar effects). This is typically explained because neurotrophins are both survival factors and axons guidance and targeting molecules, and their instructive role depends on them being produced in limiting amounts. Both deficit and excess disrupt the information carried by gradients of secreted survival/guidance molecules.

The text has been edited to clarify this point.

"To conclude, these data show that DNT-2 and Toll-2 are required for appropriate connectivity of L1 neurons to target Mi1 medulla neurons at M1 medulla layer."
The authors characterize neuronal morphologies but do not directly assess connectivity using synaptic markers. While defective morphologies are likely to impact connectivity, the conclusion that DNT-2 and Toll-2 are required for appropriate connectivity should be tempered. The authors should revise their wording to reflect that their data support morphological defects rather than direct evidence of altered synaptic connectivity.
In response to this criticism, we have now tested synaptic connectivity between DNT-2 and Toll-2 neurons using GRASP and Retrotango.

- GFP Reconstitution Across Synaptic Partners **(GRASP) was used to test if DNT-2 and Toll-2 neurons establish synaptic connections**. We used pre-synaptic nsyb-GFP1-10 (under LexAOP control) and the general CD4-spGFP11 (under UAS control), driven by Toll-2LexA and DNT-2Gal4 (Bloomington stock: BSC64314). This revealed very clear GRASP signal in the lamina and the medulla. The GRASP signal in the medulla is consistent with the expression of DNT-2 in Mi1 neurons, and Toll-2 in L1 lamina and medulla neurons. The GRASP signal in the lamina is consistent with DNT-2 expression in the retina and Toll-2 neurons in the lamina.

© 2026. Published by The Company of Biologists under the terms of the Creative Commons Attribution License (https://creativecommons.org/licenses/by/4.0/).

Using DNT-2GAL4 we cannot see retinal axonal projections reaching the lamina, perhaps because the DNT-2 Gal4 drives is weak or because DNT-2 is not expressed in photoreceptors but in interommatidial bristles.
During early pupal development, the retina is in close contact with the lamina. These data show that DNT-2 and Toll-2 neurons orm synaptic connections. **The data are shown in new Figure 4A,B.**

- **RETROTANGO was used to identify inputs of DNT-2 neurons**. The data show that using DNT-2 GAL4 to drive Retrotango, L1 lamina neurons are identified as DNT-2 neuronal inputs. This is consistent with the expression of DNT-2 in Mi1 medulla neurons that are known to connect to L1 lamina neurons. Furthermore, we now provide new data showing that upon DNT-2 RNAi knock-down, fewer L1 neurons were seen using Retrotango. This shows that DNT-2 is required for the establishment of connectivity between DNT-1/Mi1 and Toll-2/L1 neurons. **These new data are shown in new Figure 7A-C.**

To conclude, the new data show that DNT-2 and Toll-2 neurons are synaptically connected and that this depends on DNT-2.

DISCUSSION

"In fact, throughout animal development, between 50% (e.g. in Drosophila) and 80% (e.g. in vertebrates) are lost to naturally occurring cell death"
"of neurons" is missing before "are lost"
Thank you for spotting this mistake, that has now been corrected.

"Consistently with these findings, we have shown that the survival of L1 neurons depends on DNT-2 functioning together with Toll-2."
The authors state that "the survival of L1 neurons depends on DNT-2 functioning together with Toll-2." It seems that what they intend to convey is that DNT-2 acts as a ligand for Toll-2. The text should be clarified to explicitly indicate this ligand-receptor relationship rather than implying a cooperative function.

Done.

"These data demonstrate that DNT-2 and Toll-2 function together in visual system development."
Since the authors did not use tub-GAL80 or another temporal control to restrict gene expression specifically to development, the observed phenotypes could reflect combined developmental and adult effects. Throughout the text, the authors should revise their wording to acknowledge this limitation.
Corrected.

"Finally, interference with the normal levels of DNT-2 and Toll-2 also impaired axon targeting and dendritic morphology, consistently with the coupling between cell survival with connectivity."
The authors state that interference with normal levels of DNT-2 and Toll-2 "impaired axon targeting and dendritic morphology, consistently with the coupling between cell survival and connectivity." This statement seems tautological, as neurons that die cannot form connections. The authors should clarify whether they are referring to a specific mechanistic link beyond this obvious consequence.
It is actually the other way round: the neurons that do not form connections, die. This is classical neurotrophism, as described by the neurotrophic theory proposed of Rita Levi-Montalcini and Viktor Hamburguer. **Anyway, this text has been improved.**

"Our findings are consistent with prior reports that had shown the maintenance of cell survival to be required during neural circuit formation."
This statement seems tautological. It is generally expected that neurons must survive in order to contribute or be part of neural circuits. The authors should clarify if they are highlighting a specific mechanistic insight beyond this obvious requirement.
The text has been edited to improve clarity.

© 2026. Published by The Company of Biologists under the terms of the Creative Commons Attribution License (https://creativecommons.org/licenses/by/4.0/).

"In the medulla, Dm8 medulla neurons are produced in excess and are eliminated during connectivity to their R7 inputs (Courgeon and Desplan, 2019a). This is enabled by the cell surface molecular tags DIP in yDm8 binding Dpr11 in yR7, during synaptic matching (Courgeon and Desplan, 2019a)."
The statement that "Dm8 medulla neurons are produced in excess and are eliminated during connectivity to their R7 inputs" is both unclear and inaccurate. It is not evident what is meant by "during connectivity." Moreover, Courgeon and Desplan (2019a) show that Dm8 neurons undergo cell death before or by P40, prior to synaptogenesis. The authors should correct this statement and clarify the timing and mechanism of Dm8 neuron elimination.
By "connectivity" we meant targeting, the point when axon guidance terminates and the growth cone stops navigating, prior to establish synaptogenesis. Accordingly, Courgeon and Desplan (2019) say: "We found that correct matching is achieved through the generation of supernumerary target neurons of each subtype. Neurons that fail to connect to their corresponding R7 photoreceptor are culled by apoptosis. Recognition of future synaptic partners is achieved using a pair of interacting cell adhesion molecules from the Dpr/DIP families expressed in R7 or their Dm8 targets". **We have now clarified this point in the text.**

"Importantly, the maintenance of cell survival takes place during
connectivity, and enables synaptic matching between connecting neurons."
It is unclear what is meant by "during connectivity." Moreover, both Courgeon et al. (2019a) and Xu et al. (2018, 2022) show that these neurons (e.g., Dm8, Dm12, Dm14) undergo cell death before or by P40, prior to synaptogenesis. The authors should clarify the timing and mechanism of cell survival and revise this statement accordingly.
This has now been reworded.

"By contrast, it has also been proposed that apoptosis plays a minor role in cell number control during visual system development, depending instead on cell proliferation and spatial patterning through Dpp/BMP signalling (Malin et al., 2024). However, those findings were based on events taking place at the larval third instar wandering stage, when proliferation and spatial patterning are prevalent, whereas apoptosis peaks in pupa."
It seems that the authors are trying to suggest that different mechanisms control cell numbers at different developmental stages: during larval neurogenesis (L3), cell numbers are regulated primarily by proliferation and spatial patterning, whereas in the pupal stage, neuronal survival via apoptosis plays a key role. If this is the intended point, it should be stated more clearly, as the current comparison to Malin et al. (2024) is confusing and does not make this distinction explicit.
Thanks for this feedback, we have now improved the text.

"However, Toll-2 mutant MARCM clones generated in the pupa result in a dramatic loss of lamina neuron dendrites and aberrant axonal navigation in the medulla, as well as widespread neuronal loss (Li et al., 2020)."
This statement is puzzling. Neurogenesis occurs during the larval stage until P15, and MARCM requires progenitor cell division. The authors should clarify how MARCM clones were generated during pupation and provide the relevant experimental details in the Materials and Methods.
Here, we were referring to data published in Li et al 2020 eLife, and the details on how those clones were generated were given in that publication. There are no MARCM clones in this manuscript under review.

"Importantly, connectivity between L1 and medulla neurons takes place between 20-48h APF, during the period of naturally occurring cell death, and spontaneous activity in the optic lobe takes place at 48h, meaning at least some circuits are connected by then (Kurmangaliyev et al., 2020)."
The authors state that "connectivity between L1 and medulla neurons takes place between 20-48h APF," but no reference is provided for this timing. To my knowledge, no study has directly demonstrated this, so the authors should either provide supporting evidence or revise this statement.
Additionally, citing Kurmangaliyev et al. (2020) for spontaneous activity in the optic lobe is not appropriate for this point, as PSINA was originally described by Orkun Akin.

Thank you for the feedback, the text and the citations have been improved, and that reference has been removed.

© 2026. Published by The Company of Biologists under the terms of the Creative Commons Attribution License (https://creativecommons.org/licenses/by/4.0/).

"When altering DNT-2 or Toll-2 levels, L1 axonal terminals in the medulla were misrouted, rather than being confined to a single column. This is reminiscent of the phenotypes caused by alterations in Dscam and Fez levels (Millard et al., 2007, Peng et al., 2018)."
The authors note that altering DNT-2 or Toll-2 levels causes L1 axonal terminal phenotypes reminiscent of phenotypes caused by changes in Dscam and Fez levels (Millard et al., 2007; Peng et al., 2018).
However, they only reference these previous studies without discussing whether there could be a shared mechanism. While these comparisons are interesting, the manuscript would benefit from either a deeper discussion of potential mechanistic links or a clear statement that the comparison is purely phenotypic.
We did not carry out any tests of whether these phenotypes are linked, and we could not find any published work that could provide any insight on whether Dscam, Fez and Tolls could share common mechanisms. Thus, the comparison is purely phenotypic.
We have added a comment to clarify this point.

"As DNT-2 is secreted in medulla neurons and Toll-2 is expressed along neurons that connect in the medulla (e.g. L1, Mi1, Tm3, Dm9, T4), DNT-2 could help keep connecting neurons together during dynamic cellular events in development."
This sentence is poorly written and vague. It is unclear what "keep connecting neurons together" means mechanistically. Likely, DNT-2 is secreted by postsynaptic medulla neurons (e.g., Mi1), whereas Toll-2 is expressed in neurons innervating the medulla (e.g., L1, Mi1, Tm3, Dm9, T4). The authors should rephrase this sentence to clearly convey their mechanistic and cellular interpretation.
We have removed those sentences, thank you for the feedback.

FIGURES

Figure 1
Arrowheads point to what?
OL orientations should be described in the figure captions
done, thanks.

Figure 5
The title of Figure 5 ("Altering the levels of DNT-2 and Toll-2 modifies L1 axon targeting at medulla M1 layer") is misleading. The correct layer targeting is preserved; what changes is the pattern of unicolumnar innervation. When DNT-2 or Toll-2 levels are altered, L1 neurons innervate multiple columns rather than maintaining their normal single-column specificity. The title should be revised to reflect that the defect is in columnar specificity rather than layer targeting.

Thank you for the feedback, this has now been corrected.

Supplementary Figures S1 to S6 Combined UMAPs showing the expression of spz-1, -3 (DNT-3),-4 and -5 (DNT-2) and Toll-1, -2 (18w), -6 and -8 (Tollo) in distinct cells over time.
Information about which UMAP corresponds to which time point is missing.
The time points were written on the figure filenames, and we have now also added them to the figures.

MATERIALS AND METHODS
Genetics.
Please see S1 Table for the list of the stocks used and Table S3 for full genotypes for each experiment. Table S3 is not the full genotypes for each experiment. This information is partly available in the Source Data excel file
Genotypes given in abbreviated form (i.e. with the symbol “>” meaning GAL4/UAS) and emphasising what is knocked-down or over-expressed rather than the chromosomal location, were given in Table S4 with the statistical analysis and sample sizes.

Reviewer #1 (Significance (Required)):

© 2026. Published by The Company of Biologists under the terms of the Creative Commons Attribution License (https://creativecommons.org/licenses/by/4.0/).

During development, neurons are initially produced in excess. One mechanism by which the final neuronal numbers are refined relies on trophic support, which maintains the survival of necessary neurons, while excess neurons are eliminated.

In the Drosophila optic lobe, a wave of apoptosis occurs during pupation, peaking at a critical period thought to be essential for establishing final neuronal numbers and supporting proper neural circuit formation. However, the mechanisms underlying this developmental process remain poorly understood. In this manuscript, Alshamsi et al. investigate this wave of apoptosis by examining the role of Drosophila neurotrophins (DNTs), which are encoded by spätzle (spz) paralogous genes and signal through Toll receptors to regulate neuronal survival during brain development.
The authors use translation reporters to demonstrate that DNTs and Toll receptors are differentially expressed across various neuronal types innervating all optic lobe neuropils during development. They then focus on DNT-3 and DNT-2, which they show to be necessary for controlling neuronal numbers, likely by maintaining neuronal survival during pupal stages.
Notably, the results reveal a previously uncharacterized interaction between DNT-2 and Toll-2. The findings suggest that DNT-2 acts as a neurotrophic factor produced by medulla intrinsic neurons, binding to the Toll-2 receptor expressed in other neurons innervating the medulla. By examining Toll-2+ L1 neurons, which are postsynaptic in the lamina and presynaptic in the medulla, the authors provide compelling evidence that DNT-2/Toll-2 signaling regulates L1 neuronal numbers. Interestingly, the authors also show that disruption of DNT-2/Toll-2 signaling affects L1 axonal and dendritic morphologies. However, the extent to which changes in neuronal survival and neuronal morphology are mechanistically or cellularly linked is not addressed.
These findings are consistent with previous reports showing that DNTs and Toll receptors regulate neuronal survival in embryonic, larval, and pupal ventral nerve cords, as well as in the adult. Importantly, DNTs and Tolls can also promote cell death, highlighting their dual role in controlling neuronal number and circuit formation.

While the data is for the most part solid, I have concerns regarding the execution, interpretation of certain results and the conclusions drawn.
Additionally, references to previous work are often incorrect or incomplete; I provide several examples below along with non-exhaustive suggestions for improvement. Finally, the manuscript would benefit from careful text revision and improvements to figure presentation, for which I also offer non- exhaustive guidelines.

Overall, I would recommend this manuscript undergo revision before its publication and I would be happy to reassess a revised version that addresses the comments above.

We are very grateful for the positive evaluation of our work by Reviewer 1. Mot particularly, we are very grateful for the exhaustive feedback that has helped improve our work. We have followed the recommendations and we very much hope that this Reviewer finds this new version satisfactory for publication.

Reviewer #2 (Evidence, reproducibility and clarity (Required)):

Summary:
The authors investigate the role of neurotrophins in development of the Drosophila visual system, where multiple waves of cell death eliminate populations of neurons. Using transgenic reporter lines, the authors examine expression of four spz genes, which encode ligands and four Toll receptor genes in the optic lobe. The authors argue that two of these ligands, spz-3/DNT-3 and spz-5/DNT-2, are expressed in the optic lobe concurrently with a wave of cell death. Through gain- and loss of function studies they argue that DNT-3 promotes survival through Toll-8 whilst DNT-2 promotes survival through novel interactions with Toll-2 (with a focus on the lamina neurons). Finally, the authors argue that DNT-2 and Toll-2 regulate L1 neuron targeting and size.

The involvement of neurotrophins in neuronal apoptosis has not been explored in detail in the visual system, making this a potentially interesting topic. However, there are several issues with the manuscript: The primary concern is that most of the authors' conclusions are not supported by the data presented. Many of the figures are difficult to interpret without the addition of appropriate markers. In addition, there are several inconsistencies both within the manuscript and within the context of existing literature that are not addressed. For example, the authors have not demonstrated that lamina neurons undergo apoptosis. This point is critical, as apoptosis,

© 2026. Published by The Company of Biologists under the terms of the Creative Commons Attribution License (https://creativecommons.org/licenses/by/4.0/).

particularly that likely to be affected by neurotrophic factors, fine-tunes multicolumnar neuron numbers after they establish connections with their partners. On the other hand, unicolumnar neurons such as those in the lamina are not thought to need much fine-tuning because of their unicolumnar status. This distinction is not addressed in the manuscript. At present, the evidence presented does not convincingly support the authors' conclusions, and substantial further work is needed before the claims can be firmly established. Overall, these limit the credibility and significance of this study.

We thank this Reviewer for taking the time to scrutinise our manuscript and for their constructive feedback. We are very pleased that the Reviewer finds the topic interesting. Regarding the evidence for apoptosis in the lamina, it might have escaped the Reviewer that we had shown that:

(1) that the apoptotic marker anti-Dcp1 can be found in the lamina of wild-type optic lobes and apoptosis, it increased in DNT-2 or -3 mutants (old Figure 3E-F) and upon Toll-2RNAi knockdown (old Figure 4A,B).
(2) Toll-2 is expressed in lamina neurons (old Figure 2E) and the increased apoptosis seen with Dcp1 in the lamina in DNT-2 mutants resulted in total Toll-2+ lamina neuron loss (old Figure 4C,D)
(3) Using an L1 split-Gal4 reporter, we had shown that Toll-2 RNAi knock-down resulted in a severe phenotype with partial penetrance in which virtually all L1 lamina neurons were missing.
(4) We had had shown that over-expression of DNT-2 and DNT-3 rescued Dcp1 in the lamina
(5) and that over-expression of DNT-2 increased the number of lamina neurons.

Altogether, we had provided evidence that there is apoptosis in the lamina, that can be rescued by DNT- 2 and DNT-2, and that the survival of lamina neurons depends on DNT-2, DNT-3 and at least Toll-2.

Nevertheless, we now provide further evidence, to address the comments raised by this Reviewer.

Major comments:

In general, the conclusions drawn about neuropil regions and specific cell identities are not supported by sufficient evidence. Across the figures, the identity of neurons and neuropils remains unclear due to the absence of subtype-specific markers or neuropil labelling that would place the data in the anatomical context of the optic lobe.

After looking at the overall expression patterns of various DNT/Spz family ligands, we focused a more in- depth analysis into DNT-2 and Toll-2. We claimed that DNT-2 is expressed in Mi1 neurons and Toll-2 in L1 lamina neurons. L1 and Mi1 neurons are well known to form synaptic connections. We also claimed that Toll-2 is also expressed in L3 neurons. We appreciate the Reviewer's comments that this claim required to be supported by Mi1 and L1 specific markers.

We have now obtained new data that show:

- **Co-localisation of the Mi1 marker anti-Bsh in DNT-2+ neurons** visualised with DNT-2>6xmCherry. This demonstrates that DNT-2 is expressed in Mi1 neurons. **New Figure 3A-C.**

- **Co-localization of the L1 marker anti-Svp in L1 Toll-2+ neurons** labelled with Toll-2Gal4 UAShis- YFP, demonstrating that Toll-2 is expressed in L1 neurons. **New Figure 3D,E.**

- **Co-localization of the L3 marker anti-Erm in L3 Toll-2+ neurons** labelled with Toll-2Gal4 UAShis- YFP, demonstrating that Toll-2 is expressed in L3 neurons. **New Figure 3F,G.**

(1) The authors claim that spz and Toll genes are differentially expressed in the optic lobe throughout pupal development. However, the data presented do not convincingly support this conclusion. The primary concern is that the Gal4 reporter insertions used to infer gene expression do not match published single-cell transcriptomic datasets (as noted by the authors). This discrepancy calls into question the accuracy of these reporters (or the

© 2026. Published by The Company of Biologists under the terms of the Creative Commons Attribution License (https://creativecommons.org/licenses/by/4.0/).

transcriptomics) and raises uncertainty about which dataset can be trusted. Given that these data are inconsistent, gene expression should be assessed directly by HCR to draw reliable conclusions.

The discrepancy between single-cell-RNAseq data, reporter expression and protein translation is common. This is explained because: (1) the scRNAseq data reveal a very limited temporal snapshot of gene expression. Our meta-analysis of these transcriptomic data revealed that the expression of these genes is limited and dynamic. (2) Reporter gene expression is subject to multiple levels of gene regulation and protein translation that scRNAseq data are not. That is, Gal4/UAS expression requires first Gal4 to be expressed and translated, to then subsequently drive the expression of a reporter gene, which in turn has to be translated. Thus, there is necessarily a temporal delay between detecting the transcript and the protein reporter (i.e. GFP). (3) The perdurance of reporter proteins contribute to reporter patterns. (4) Gal4 introduces an amplification, which is missing from the endogenous transcriptomic samples. This means that a gene is expressed in very low levels in scRNAseq can be easily visualised with GAL4/UAS. (5) Like all methods, scRNAseq also bears variability and error. To conclude, whilst it is interesting to obtain information from scRNAseq data as well as from visual reporter gene expression, it would be erroneous to make inferences from direct, exact comparisons.

With the exception of Toll-8Gal4 (which is a transposon-based insertion upstream of the Toll-8 coding region), **all the other Gal4 driver lines we used should reproduce the endogenous expression pattern of the relevant genes because:** (1) spz-1-T2A-Gal4, spz-3-T2A-Gal4, DNT-2Gal4, spz-4-T2A-Gal4 and Toll-1Gal4 were all engineered using the **T2A self-cleaving peptide**. With this method, mRNA is transcribed from each of these genes (e.g. spz-1, spz-3 etc), and during splicing, engineered **mRNA encoding for T2A plus Gal4 is spliced in frame** with the previous genes, generating **fusion proteins** (eg. Spz-1-T2A-Gal4 fusion protein). The self-cleaving T2A peptide then cleaves itself, **liberating the Gal4 transcription factor in the cells that expressed the mRNA for the gene of interest** (eg spz-1). In other cases, Toll-2Gal4 reproduces the endogenous pattern of Toll-2 because the coding sequence for Toll-2 was replaced by that of Gal4 (Li et al 2020 eLife). Toll-6Gal4 reproduces the endogenous pattern of Toll- 6 because Gal4 is inserted into the single Toll-6 exon (see McIloy et al 2013 Nature Neuroscience). For these reasons, we considered it unnecessary to carry out HCR experiments as they would be highly unlikely to reveal any novel information not already revealed by these reporters.

Related comments: The authors claim that spz and Toll genes are expressed differentially in the optic lobe throughout pupal development. However, the characterisation of these expression patterns is lacking in the following aspects:

- Time courses are inconsistent and incomplete across the different genes.

The experiments were carried out at equivalent time points for all genotypes, but for some genotypes (eg spz-4) we did not detect any expression at the earlier time points, as we had explained in the text.

- The conclusion that DNT-2/3 are expressed concurrently with cell death lacks evidence is not supported: DNT2/3 expression does not appear restricted to a clear time window and without co- staining for apoptotic markers this conclusion cannot be substantiated.

In response to this point, we have carried out new experiments and **provide new evidence that DNT- 2>6xmCherry is co-expressed during the period of naturally occurring cell death, visible with anti-Dcp1 stainings. These new data are provided in new Figure 5A.**

- There is insufficient evidence to support the claim that spz-2 is specifically expressed in Mi1 neurons or that Toll-8 is specific to L4/L2. These claims are largely based on the transcriptomics data, which were inconsistent with the reporters in many other contexts. There are no neuron type markers costained to validate this claim.

Presumably the Reviewer means DNT-2 (also called spz-5), as we did not provide any data on spz-2 (also called DNT-1) in this manuscript. We showed the patterns of multiple Tolls in the optic lobe in Figure 2, and we carried out a more in-depth study of the relationship between DNT-2 and Toll-2. We had claimed that DNT-2 (also called spz-5) is expressed in Mi1 neurons and Toll-2 in L1 lamina neurons, based on neuronal morphology and location. In response to the criticism by this Reviewer, **we have now provided new data that demonstrate the identity of DNT-2 and Toll-2 expressing neurons:**

© 2026. Published by The Company of Biologists under the terms of the Creative Commons Attribution License (https://creativecommons.org/licenses/by/4.0/).

- **Co-localisation of the Mi1 marker anti-Bsh in DNT-2+ neurons visualised with DNT- 2>6xmCherry**. This demonstrates that DNT-2 is expressed in Mi1 neurons.
These new data are provided in New Figure 3A-C.

- **Co-localization of the L1 marker anti-Svp in L1 Toll-2+ neurons labelled with Toll-2Gal4 UAShis- YFP,** demonstrating that Toll-2 is expressed in L1 neurons.
These new data are provided in New Figure 3D,E.

- **Co-localization of the L3 marker anti-Erm in L3 Toll-2+ neurons labelled with Toll-2Gal4 UAShis-YFP,** demonstrating that Toll-2 is expressed in L3 neurons.
These new data are provided in New Figure 3F,G.

- Sample sizes are too low for some reporter lines (~2-4).
We respectfully disagree that the Reviewer. Whereas large sample sizes are necessary for functional genetic analyses (ie of loss and gain of functions conditions and mutations), in the context of the question we asked, the description of wild-type expression profiles does not require large sample sizes. N=3 is the gold standard for many experiment types.

(2) The authors quantified cell death using Dcp-1 signal volume and concluded that overexpression of DNT-2 or DNT-3 reduces cell death, while loss of either increases cell death in the lamina. However, the lamina is not labelled with an appropriate marker, nor are neuropils labelled to make the structure recognisable making it unclear whether this wave of cell death is truly occurring in the lamina. This is a recurring issue throughout the manuscript.
The Dcp1 experiments were carried out without counterstaining (eg N-Cadherin) because anti-Dcp1 is a rather tricky antibody and works best when used alone. **Importantly, we are confident we identified lamina signal correctly and we now provide further evidence in revised Figures 5A,B and 6A.**

Images supplied for the referees below have been removed and included in Figure 5A and Figure 6A of the manuscript.

In early pupal development (24 APF), the lamina is very tightly associated to both the retina and the medulla. In vertical mounts, the lamina physically detaches from the medulla, but in **horizontal mounts it does not, and the lamina is sandwiched between the retina and the medulla.**

The Dcp1 analysis was carried out on brains mounted horizontally, because the vertical mounting does not allow accurate visualisation of dying cells and their quantifications in 3D, whereas horizontal mounting does.

We now provide new Figures 5A,B and Figure 6B to illustrate this point.

Related comments: the reported values for Dcp-1 signal volume differ dramatically between the Gal4 (~40,000) and mutant (~2,000) controls, without an explanation for this discrepancy.
Yes, we noticed that, but all the samples were checked carefully for staining quality and they were good. The **control genotypes were indeed different**: for Figure 5B-C the genotype of the control was **wild-type Oregon**, whereas for Figure 5D-E and Figure F-G the control genotypes were ***Toll-8Gal4/+*** **and** ***nsybGal4/+***, respectively. Importantly, the two controls over Gal4 resulted in comparable Dcp-1 levels. Conceivably, Gal4 may contribute to the phenotype. Other considerations can also explain the variability in Dcp1- levels in control samples (e.g. anti-Dcp1 stainings are tricky, fixation, staining, confocal settings in different experimental blocks, laser ageing, etc.). The person carrying out the experiments (first author Naser Alshamsi) would also be improving their skills as they acquired more data. Most importantly, **all genotypes for each experimental block** (i.e. Figure 5B-C loss of function, Figure 5D-E Spz-3/DNT-3 gain of function and Figure 5F-G DNT-2 gain of function) **were handled and scanned as contemporaneously as practically possible and confocal settings were maintained constant within each of these groups.** Doing these experiments required very hard work over various years, and it was impossible to repeat all these experiments

© 2026. Published by The Company of Biologists under the terms of the Creative Commons Attribution License (https://creativecommons.org/licenses/by/4.0/).

for the revision. Most importantly, we are confident that the data were of good quality to reveal appropriate findings and that the conclusions are correct.

We have now added a clarification in the methods section.

(3) The claim that DNT-2 and DNT-3 promote survival via Toll-2 and Toll-8, respectively, is not sufficiently supported. The authors do not show that manipulation of each ligand specifically affects neurons expressing the relevant receptor. To substantiate this conclusion, the specificity of ligand manipulation should be tested. For example, through compensation experiments or by demonstrating that DNT-2 loss of function specifically induces cell death in Toll-2+ neurons.

We had already shown exactly that: DNT-2 complete loss of function in null *DNT-2^{18}/DNT-2^{37}* mutants leads to the total loss of Toll-2+ neurons, **shown in Figure 4C-F of original manuscript (Figure 6C-F of R1 revised version).**

Furthermore, we had also shown that over-expression of DNT-2 resulted in an increase in thee number of Toll-2+ neurons. **This was shown in Figure 4C-F of original submission, and Figure 6C-F of R1 revised version.**

(4) The authors further investigate a potential interaction between DNT-2 and Toll-2, concluding that DNT-2 promotes survival in the lamina, medulla, and lobula complex through Toll-2. However, no evidence is presented showing altered levels of apoptosis in the medulla or lobula/lobula plate following DNT-2 manipulation. The conclusion that DNT-2 promotes survival via Toll-2 is based on a Toll- 2RNAi;DNT-2FL epistasis experiment, but this interpretation does not account for the reduced expression of each construct due to Gal4 titration. This issue also applies to subsequent experiments (Figs. 5 and 6). A Gal4 titration control is required for each of these experiments to exclude this confound.

It appears that the Reviewer missed these data, but the evidence that DNT-2 (and DNT-3) promote cell survival also outside the lamina (i.e. in medulla and lobula complex) **had been given in the original submission in Supplementary Figure 8**. Here, we showed the quantification of the apoptotic marker Dcp1 outside the lamina, upon over-expression of DNT-3/spz-3 in Tol-8+ neurons and over-expression of DNT-2 in all neurons. Both gain of function genotypes resulted in a decrease in cell death, meaning that DNT-2 and -3 promote neuronal survival.

The Reviewer seems to have also missed the data showing that loss of function for DNT-2 resulted in the compelte loss of all Toll-2+ neurons in the medulla and lobula complex. These data had been shown **Figure 4E-F of the original submission (Figure 6E-F of R1, revised version)**.

The conclusion that DNT-2 promotes cell survival via Toll-2 was based not only on the epistasis result mentioned above, but altogether on the following data:

(1) DNT-2 mutants have increased Dcp1+ apoptosis in the lamina
(2) DNT-2 over-expression decreased naturally occurring Dcp1+ cell death in the lamina
    Together these two data sets show that DNT-2 is required to maintain cell survival in the lamina.
(3) Toll-2-RNAi knock-down increased Dcp1+ apoptosis in the lamina
(4) Over-expression of DNT-2 could not promote cell survival if Toll-2 was knocked-down
(5) Heterozygosity for Toll-2 induced lethality of otherwise viable DNT-2^{37}/DNT-2^{18} homozygous mutants.
(6) Over-expression of DNT-2 increased the number of Toll-2+ neurons
(7) DNT-2^{37}/DNT-2^{18} mutants lacked virtually all Toll-2+ neurons
    Together, these data show that DNT-2 promotes cell survival in the optic lobe via Toll-2 and that apoptosis in the absence of DNT-2 or Toll-2 functions leads to cell loss.

We respectfully disagree with this Reviewer, but – in view of the above - we did not consider that titration controls were necessary. A “titration of Gal4” problem – ie, if Gal4 must bind 2 distinct UAS sequences there could be a dilution of Gal4 leading to lower expression from both – would not explain the observations that DNT-2 over-expression increased the number of Toll-2+ neurons whereas virtually all Toll-2+ neurons were lost in DNT-2 mutants.

© 2026. Published by The Company of Biologists under the terms of the Creative Commons Attribution License (https://creativecommons.org/licenses/by/4.0/).

(5) The authors conclude that DNT-2 production in Mi1 medulla neurons is required for L1 connectivity, survival, and morphology. However, these conclusions are based on manipulating DNT-2 and Toll-2 expression in L1 neurons, which does not directly test the requirement for DNT-2 in Mi1 neurons.

Contrary to this Reviewer's assessment, the conclusion was not only reached based on the phenotypes after manipulating DNT-2 and Toll-2 in L1 neurons.

Reviewer 2 missed the fact that for the analysis of cell survival in the lamina, we had analysed phenotypes in ***DNT-2^{37}/DNT-2^{18}* null mutants.** As the mutants were null, and DNT-2 is expressed in medulla Mi1 neurons but not in lamina neurons, the increase in lamina cell death in DNT-2 mutants was due to the loss of DNT-2 function in Mi1 neurons – **see Figure 3E,F and Figure 4C-F of original manuscript**. We had also shown in *DNT-2* mutants virtually all Toll-2+ lamina neurons (which we have now confirmed include L1 neurons) were lost. We had also shown that in DNT-2 null mutants apoptosis increases (Figure 3E,F, **new Figure 5B,C**) and **all Toll-2+ lamina and medulla neurons are lost** (Figure 4C- F, **new Figure 6C-F**). And we showed that DNT-2 rescues naturally occurring cell death (original Figure 3C,D) functioning as a ligand for Toll-2, as this was prevented if Toll-2 was knocked down (original Figure 4A,B).

We have now improved these data by showing that:

- DNT-2 is expressed in Mi1 neurons, which **we demonstrate** using the Mi1 marker anti- Bsh (**new Figure 3A-C**).
- Toll-2 is expressed in L1 neurons, which **we demonstrate in this revision** with the L1 marker anti-Svp (**new Figure 3D,E**).

Consistently with the above data, we had also shown that **Toll-2 RNAi knock-down resulted in a dramatic loss of L1 neurons** (original Figure 7B) and that **over-expression of DNT-2 could increase L1 dendrite size and this could be rescued by knocking down Toll-2** (original Figure 8).

Taken together, the above data show that a source of DNT-2 ligand in Mi1 neurons regulates survival, axonal connections and dendrite shape of Toll-2+ L1 (plus survival of medulla neurons).

In addition, the image quality in Figure 5 is problematic: brightness and contrast differ visibly between panels, making them challenging to interpret.

It appears that the Reviewer did not understand the phenotypes shown in original Figure 5. The Reviewer confused "brightness and contrast" with the fact that **many cells are missing** in the Toll-2-RNAi knock-down genotypes. Toll-2 knock-down caused two phenotype types, with 50% penetrance: a mild and a severe phenotype. **In the severely affected optic lobes, there were hardly any L1 neurons left**, due to the fact that Toll-2 knock-down caused lamina apoptosis (as shown in original Figure 4A,B). As there are hardly any L1 neurons left, there is no green signal in Figure 5B – which presumably led the Reviewer to think it was a brightness problem, but it is not: the neurons are missing.

Finally, the discussion states that "loss of function for DNT-3 (spz-3) causes lamina cell death that is not naturally compensated for by DNT-2," but no evidence is presented in this manuscript to support that conclusion.

We showed that *DNT-2* over-expression and *DNT-3* over-expression can both rescue naturally occurring cell death. We also showed that in *DNT-2* and *DNT-3* single mutants, cell death increased. If DNT-2 and DNT-3 could compensate for each other, then loss of function for one of them (eg *DNT-3*) would be compensated by with an increase in DNT-2 production or release, resulting in a wild-type phenotype (ie no difference in Dcp1+ signal compared to controls). For example, there are multiple Dilps in Drosophila, and mutations or knock-down in one Dilp do not produce a phenotype because other Dilps can compensate for the missing Dilp and cover their function. This is not what we observed: we observed that mutations in only DNT-2 or only DNT-3 caused an increase in cell death. This means that compensatory mechanisms were not induced, suggesting that each of these ligands regulates cell survival in distinct neuronal types or by binding distinct

© 2026. Published by The Company of Biologists under the terms of the Creative Commons Attribution License (https://creativecommons.org/licenses/by/4.0/).

Toll receptors. **We have now edited the discussion to improve clarity, thank you for the feedback.**

Other general comments:
-Lack of labels for each individual image makes figures and text difficult to interpret/reference.
We have now changed this for most figures.

-Gene names not italicised in figures
We have now corrected this.

-Inconsistent aspect ratio for graph axes (Obvious in figure 5C,D)
We have now corrected this.

-Image borders often not completely vertical/horizontal making them appear jagged in some figures e.g. Figure1A.
This has now been corrected.

-General typos e.g. (n=10 10 brains), inhibitor pf apoptosis p35
Thank you, now corrected.

-Lobula complex should not be used to refer to lobula plate and lobula plug at this stage as they are distinct neuropils
Unfortunately, the Reviewer did not provide enough information on the stage they refer to for us to improve this point. We used the term lobula complex when it was not possible to unambiguously identify the neuropil – lobula or lobula plate - i.e. in horizontal views, and in samples not counterstained with NCadherin.

-Figure 1:
-Arrows not explained
Now done in figure legends.

-N-Cadherin absent from some images without explanation
This affects Figure 1. We agree with this Reviewer that it would have been ideal to have all the images counterstained with N-Cadherin. We considered removing the N-Cadherin channel from all images, as in Figure 2 we did not show it either. However, we opted for keeping the N-Cadherin counterstain when possible, as it is very helpful to identify the distribution of sparsely distributed ligands. Unfortunately, we have been unable to improve the images in old Figure 1A,B24h and D 48h. This was work carried out in the greatest part by a PhD student. Unfortunately, we are unable to repeat more experiments to add N-cadherin to all samples, as the PhD student working on this project left the lab already. I had to train from scratch two new members of my team to carry out the remaining revisions for this paper, but the learning curve was steep and they were at the limit of how much they could do, whilst also working on their own projects.

-First two images of 1B are identical.
Thank you for spotting this lapsus, this has now been changed.

-Images in panel B at different timepoints and spz-4 is missing without explanation.
The aim was simply to show that some of these genes have abundant expression in the medulla, and therefore can have broad-ranging effects in medulla development. We did not include spz-4 in this panel because we only found clear expression in the trachea.

Figure 3:
-Dark spot in DNT-3FL 3A image.
Thank you for drawing this to our attention, these images have been replaced for better ones.

-Descriptions in text often lack specific details "Dcp1+ apoptosis in the lamina and outside the lamina too."
This has now been improved.

© 2026. Published by The Company of Biologists under the terms of the Creative Commons Attribution License (https://creativecommons.org/licenses/by/4.0/).

Supplementary:
-Figures S2-S6 lack labels denoting the timepoint of each figure
This was previously included in the filename but it has now been added to the figures too.

-Figure S7 is illegible
It can be read magnifying it in a screen. Unfortunately, there is nothing we can do to improve this, but the resolution is good after magnification.

-Supplementary figure 8 is referred to as S9 in text and is missing genotype information as well as neuropil labels on images
Thank you for spotting this error.
The genotypes were all there in the original version, colour-coded as in the graphs. We have now included the actual names to the images as well. The dotted line ROI indicates the optic lobe excluding the lamina. Dcp1 signal is widespread across the optic lobe, horizontal mounting makes cell counting and signal intensity measurements more robust, but this meant it was not possible to unambiguously identify medulla from lobula complex – other than from the position in the stack, with the medulla being anterior and the lobula complex posterior - in these stainings. **A clarification is included in the figure legend.**

Reviewer #2 (Significance (Required)):

Significance: This is an interesting topic of investigation, and the findings-if substantiated-would be of interest to the field of Drosophila visual system development and programmed cell death. In particular, it would be valuable to place these results in the broader context of programmed cell death as a mechanism for eliminating excess multicolumnar neurons during visual system development. However, at present the evidence presented does not convincingly support the authors' conclusions, and substantial further work is needed before the claims can be firmly established.

We are very pleased that Reviewer 2 found our work interesting. We have made substantial changes to the figures and manuscript and we hope Reviewer 2 is satisfied with these changes.

Reviewer #3 (Evidence, reproducibility and clarity (Required)):

Summary:

Building a functional nervous system with the correct number of correctly specified and wired pre- and post-synaptic neurons requires a tight control of proliferation and cell survival during development.
Focusing on the latter, Naser Alshami and colleagues in the laboratory of Alicia Hidalgo investigated the role of three neurotrophins and their respective Toll receptors in regulating neuronal survival in the visual system of Drosophila. A combination of expression and genetic loss-of-function and gain-of- function analyses revealed a novel role for the neurotrophin DNT2 (spaetzle-5) in controlling survival, dendritic spine formation, columnar axonal innervation of lamina neuron L1 expressing the receptor Toll-2.

Major comments:

- DNT-2 (spz-5) is described to be expressed in non-neuronal cells in the retina. However, the clustered arrangement of cells in cross-sections of the retina during pupal development (72h DNT-2 bottom panel) suggests that photoreceptors could be labeled by the reporter as well. This should be assessed by co- labeling experiments with Elav and/or mAb24B10. This is important, as photoreceptors may represent a presynaptic additional source of this neurotrophin to lamina neurons L1 (see below).

We agree with this reviewer that determining whether the retina could also be a source of DNT-2 is an interesting question. In response to this Reviewer's comments, we repeated this experiment by dissecting the pupae at 24h and 48h APF, but we were unable to detect signal in the retina at 24h APF using DNT-2GAL4 and UAS-6xmCherry. The DNT-2-Gal4 driver line is always rather weak, which presumably means that the expression of the DNT-2 gene is tightly regulated and in low

© 2026. Published by The Company of Biologists under the terms of the Creative Commons Attribution License (https://creativecommons.org/licenses/by/4.0/).

levels. Similarly, mammalian neurotrophins are also expressed in limiting amounts. In situ hybridisation in larval CNS did reveal signal in the retina in the wandering larval stage (Ben Sutcliffe PhD thesis, 2013), although those data did not have the resolution to determine what cell types were the source of DNT-2. Using various reporters (i.e. myrGFP or 6xmCherry), we could detect DNT-2 expression in retinal cells from 72h APF onwards. On recommendation from an optic lobe expert – Dr Fernando Casares - and after reading further publications (e.g. Ready…), we conclude that the retinal cells expressing DNT-2 most likely correspond to inter-ommatidial bristle neurons. We tried to carry out double stainings with photoreceptor markers (e.g. 24B10) and anti-Elav, but unfortunately we were unable to confirm that the expressing cells were neurons but not photoreceptors.

We have added a sentence to include this information, in the results section and Figure 1 I,J legend.

- The most right hand panel of Figure 2A, first row, Toll-2, does not resemble a 24h APF optic lobe, and would need to be replaced by a correctly staged younger sample. This is relevant, as the image at 48 h APF shows extensive staining in a cluster at the medulla edge, suggesting that expression in medulla neurons may be more abundant at younger stages. The expression of Toll-2 in L3 neurons is not unambiguously demonstrated at the angle shown in Figure 1E first panel. Normally, MCFO clones should be able to reveal single cells instead of L1 and L3 together.

According to our experimental records, image **Figure 2D** (new labelling for R1 version) is of a 24h APF sample. In fact, there is strong and abundant medulla signal in this sample, as the Reviewer predicted for this stage.

Unfortunately, we were unable to obtain single cell Toll-2>MCFO clones for L3 neurons. Nevertheless, in response to Reviewer 3's subsequent comments (see below) we carried out a staining using anti-Erm which demonstrated that Toll-2 is expressed in L3 neurons. **These new data are now provided in Figure 3F,G.**

- Related to this part of the study, the authors describe that Toll-2 is expressed by connecting neurons (L1, Mi1, Tm3...). This is solely based on RNAseq data. In the next paragraph, the authors describe that Toll-2 is expressed in L1 and DNT-2 in Mi1 (or related) medulla neurons. However, this is rather vague, as it is not clear what "related" medulla neurons refers to. As expression in Mi1 neurons is at the heart of this study, it would be essential to verify that Mi1 is included in the Toll-2-Gal4 and DNT-2 expression pattern. Indeed, in Figure 1A (right panel, row 2, 48 h APF) expression of DNT-2 in this neuron subtype may be visible. This should be verified by markers and single cell labeling such as MCFO. Finally, the authors mention that the DNT-2 ligand could "reach" the Toll-2 receptor. Here some more precision would be helpful, as it is not clear, how far neurotrophins would need to travel, and synaptic contacts may form too late for mediating survival. Furthermore, there seems to be also the possibility that there is a substantial contribution of autocrine signaling, if L1 neurons indeed express both the ligand and the receptor, which would need to be presented more clearly.

We have now carried out these tests and provide the following new evidence:

- We used anti-**Bsh as Mi1 neuronal marker and labelled DNT-2 neurons with UAS-6xmCherry**, and this showed co-localisation in medulla cells, demonstrating that medulla neurons expressing DNT-2 are indeed Mi1 neurons. **These new data are provided in new Figure 3A-C.**

- We used **anti-Svp as L1 neuronal marker and labelled Toll-2 neurons with UAS-mCD8GFP** and this showed co-localization in L1 neurons, demonstrating that Toll-2 neurons are expressed in L1 neurons. **These new data are provided in Figure 3D,E.**

- We used **anti-Erm as L3 neuronal marker and labelled Toll-2 neurons with UAS-mCD8GFP** and this showed **co-localization in L3 neurons**, demonstrating that Toll-2 neurons are expressed in L1 neurons. **These new data are provided in Figure 3F,G.**

Regarding autocrine signalling, our the data did not reveal natural expression of DNT-2 in L1 neurons, but scRNAseq data indicate that Toll-2 is expressed in Mi1 neurons which we showed

© 2026. Published by The Company of Biologists under the terms of the Creative Commons Attribution License (https://creativecommons.org/licenses/by/4.0/).

express DNT-2. We did express DNT-2FL ectopically in L1 neurons (in old Figure 5 and 6). We have published before that DNT-2FL is naturally secreted and cleaved (McIlroy et al 2013 Nature Neuroscience; Foldi et al 2017 J Cell Biol), and in such experiments it can activate the receptor of the cell releasing the ligand (e.g. Sun et al 2024 eLife). In this manuscript, we demonstrate using epistasis that ectopically expressed DNT-2 in L1 neurons is functional and can be rescued by Toll-2 knock-down in those same neurons.
Unfortunately, we were unable to identity medulla or lobula complex neurons expressing Toll-2. However, we did show that Toll-2 is very abundantly expressed throughout the optic lobe (i.e. including in the medulla). Furthermore, we showed that in flies that are homozygous mutant for DNT-2 plus heterozygous for Toll-2 virtually all Toll-2+ neurons throughout the entire optic lobe are missing. This means that medulla neurons expressing Toll-2 could also respond to the release of DNT-2 from medulla neurons.

We have now edited the manuscript extensively, removing ambiguities and increasing precision and rigour. Thank you for the feedback.

- The next set of experiments uses over-expression of DNT-2 and DNT-3 in Toll-8 expressing neurons and assessment of survival rates in lamina neurons. Survival is determined by evaluating Dcp-1 signals rather than cell numbers. During normal larval development, 7 future lamina neurons per column are formed, two of which are eliminated by apoptosis. Thus, it would be important to verify whether it is these neurons that are surviving, using for instance Elav labeling; moreover lamina neuron specific markers (see Xu et al., 2024) may enable assessment whether the surviving neurons adopt specific lamina neuron identities in excess to the normal 5. It may also need to be considered that both neurotrophins could affect proliferation, if the numbers do not add up (as drivers may be active already during the third instar larval stage, this is possible).

Survival is determined by measuring the amount of Dcp-1, which drives apoptosis. Signal volume rather than cell number is used, because Dcp-1 signal is also present in the cytoplasm, Dcp1+ cells have irregular shapes, and dying cells fragment and release cell debris that could be erroneously counted as individual cells. Furthermore, as there are many dying cells at 24h APF, the signal from neighbouring cells fuses and segmentation programmes are unable to distinguish between individual cells. Thus, **measuring signal volume is the only way to accurately measure changes in Dcp-1 in these specimens.**

We agree with this Reviewer that finding out the cell fate of surviving neurons when over-expressing the ligands DNT-2/spz-5 and DNT-3/spz-3 is an interesting question. We carried out this test, as recommended. We over-expressed DNT-2 in Toll-2+ neurons labelled with Toll-2>hisYFP, and labelled lamina neurons with the lamina neuron L1 marker anti-Svp and with lamina neuron L3 marker anti-Erm. The data showed that there was colocalization between Toll-2 and Svp and Erm in different cells, confirming that Toll-2 lamina neurons include both L1 and L3 (as shown in new Figure 3D-G). **However, the excess cells did not co-label with either Erm nor Svp.** This means that the excess cells did not acquire L1 nor L3 lamina neuron identity. **These new findings are shown in Figure 6G,H.**

Conceivably, DNTs/Spzs could also influence cell proliferation during development through their Toll receptors. In fact, we have previously shown that at least Toll-2 can induce neurogenesis in pupal development and in the adult brain (Li et al 2020 eLife). However, exploring further their involvement in cell proliferation was beyond the scope of this work under assessment. The PhD student who carried out most the work for this manuscript completed his PhD and left the lab, and I do not have people who could put the huge effort required to investigate an involvement in cell proliferation.

- Gain-of-function experiments are followed by loss-of-function experiments, assessing the impact of loss of DNTs in entire animal mutants on lamina neuron survival. As for the gain-of-function studies, it would be important to know, which lamina neuron subtypes are dying in the absence of DNT-2 and DNT-Indeed, Figure 4C reveals a substantial loss of L1 and L3 neurons. The over-expression experiments of the ligands using the Toll-2 driver are complicating matters, considering that the driver line is described to be a mutant in Toll-2, and the ligand is expressed in the same neuron, creating an autocrine signaling situation. Moreover, since Toll-2-Gal4 is used to drive expression in all lamina neurons L1 and L3, and entire optic lobes are assessed, the

© 2026. Published by The Company of Biologists under the terms of the Creative Commons Attribution License (https://creativecommons.org/licenses/by/4.0/).

numbers should be around 1600 in wild type, but are not, suggesting that there might have been some cell death in controls. If there are more lamina neurons in the over- expression situation, it would mean that additional neurons are adopting L1 and L3 identity and thus reveal an additional direct role in cell fate determination by DNT-2 and Toll-2 or an indirect role considering the intricate interactions of lamina neurons to adopt specific identities via N signaling (see Xu et al. 2024). This would need to be assessed more in detail using for instance independent drivers or markers for lamina neuron subtypes.

As the Reviewer pointed out, Figure 4C,D showed that all Toll2+ lamina neurons die in DNT-2 mutants. As there are no Toll-2+ neurons visible in DNT-2 neurons, this means that both L1 and L3 neurons die in DNT-2 mutants. Contrary to the Reviewers' point above, DNT-2 is not expressed in lamina neurons, and over-expression of DNT-2 in Toll-2+ cells resulted in the increase in Toll-2+ lamina neurons. In the medulla, there could be autocrine signalling, but we don't see this to be a problem.

Regarding the number of lamina neurons, we don't think it is meaningful to speculate on the exact number shown, as there could be technical limitations (eg with the mounting, dissections), which would have contributed to the variability in the data.

In response to the point raised regarding the fate of the excess Toll-2+ cells when DNT-2 is over- expressed, we carried out new experiments for this revision. We stained Toll-2>hisYFP+ cells over- expressing DNT-2FL with anti-Svp to identify L1 neurons and with anti-Erm to identify L3 neurons. As explained above, data showed that there was colocalization between Toll-2 and Svp and Erm in different cells, confirming that Toll-2 lamina neurons include both L1 and L3 (as shown in new Figure 3D-G).
However, the excess cells did not co-label with either Erm nor Svp. This means that the excess cells did not acquire L1 nor L3 lamina neuron identity. **These new findings are shown in Figure 6G,H.**

- The statement, that the remaining signal is coming from macrophages would need to supported by additional markers or described more carefully (as it could be glia).

The signal seems unlikely to be glial, as the cells are sporadic and located in unpredictable, erratic locations – hence the reason for proposing they might be macrophages. We tried using anti-Croquemort antibodies to test whether these cells were macrophages, but unfortunately we were unable to obtain new data.
We have now edited and toned down this statement.

- Page. 9. The study reports a new interaction between DNT-2 and Toll-2 as its possible receptor. This is in part based on whole animal lethality, without providing quantification. This should be added.

We respectfully disagree that this is essential information, essential information was already provided. The *Toll-2* locus is on the second chromosome and *DNT-2* is on the third, so we used conventional genetics aiming to generate double stocks of genotype *w;Toll-2Gal4/CyO; DNT-2^{18}/TM6B*. However, only a few males with this genotype eclosed, no females, and we were unable to establish the stock. We used these males to cross to *w; UAS-HisYFP; DNT-2^{37}/TM6B* mutant females, to obtain *Toll-2Gal/UASHisYFP; DNT-2-/DNT-2^{37}* homozygous mutant pupae. However, we also obtained very few of these. We carried out these genetic protocols many times to obtain a satisfactory sample size. Unfortunately, we did not write down the number of flies that eclosed from all genotypes to verify accurately the mendelian segregations. In any case, the number of *Toll-2Gal4/UASHisYFP; DNT-2^{18}/DNT-2^{37}* mutants that make it through pupal development was so low that the combination is virtually lethal.

We did not find such a strong genetic interaction between DNT-2 and Toll-6 or Toll-7 mutants, and we have shown before that DNT-2 is the ligand of Toll-6 and can also bind Toll-7 (McIlroy et al 2013 Nature Neuroscience). We have also published before that DNTs/Spz family ligands can promiscuously bind multiple Toll and Kek receptors: in particular, that DNT-2 binds Toll-6 and Toll-7, Kek-5 and Kek-6 (McIlroy et al 2013 Nature Neuroscience; Foldi et al 2017 J Cell Biology). Hence, the genetic evidence provided in this manuscript – including that: DNT-2 promotes cell survival but this can be prevented with Toll-2 RNAi knock-down (Figure 6A-B); that in DNT-2 mutants Toll-2+ cells are missing (Figure 6C-F); and that Toll-2 knock-down prevents the

© 2026. Published by The Company of Biologists under the terms of the Creative Commons Attribution License (https://creativecommons.org/licenses/by/4.0/).

increase in dendritic complexity caused by DNT-2 over-expression (Figure 8B,D), together strongly suggest that DNT-2 is a ligand for Toll-2.

- Interestingly, knock-down of Toll-2 in all neurons led to increased cell death of lamina neurons, which cannot be overcome by over-expression of DNT-2 [FL]. It would be interesting to assess whether cell death is even higher by providing a statistical test comparing the knock-down of Toll-2 with the knock- down of Toll-2 and the simultaneous over-expression of DNT-2 in Figure 4B.

Thank you for this interesting feedback. We have now changed the statistical test to accommodate this request, and **we show that indeed there is a statistically significant difference in cell death between Toll-2RNAi and Toll-2RNAi; UASDNT-2FL samples.** We have previously published that DNT-2 is a ligand of Toll-6 (McIlroy et al 2013 Nature Neuroscience) and that Toll-6 can lead to opposite cellular outcomes (cell survival or cell death) depending on context (McIlroy et al 2013 Nature Neuroscience; Foldi et al 2017 JCB). In particular, we showed that Toll-6 can induce apoptosis in the pupal CNS (Foldi et al 2017 JCB). Perhaps in the absence of Toll-2, DNT-2 more readily activates Toll-6, favouring overall a proapoptotic pathway.
We have updated the graph in new Figure 6B, and the Table S4 with statistical details, and added the comment above in the text.

- Page 11 (also page 4). The authors describe that "connectivity of lamina neurons to medulla neurons takes place at 30-48 h APF and between medulla and lobula complex at 60-70h APF, and that expression of synaptic markers starts at 24h APF". It is not clear how the authors define connectivity (here and throughout the manuscript). While axonal and dendritic projections are established during the first half of pupal development, functional synapses are thought to be solely established from mid-pupal development onwards (early expression of synaptic markers may not be indicative of synapse formation as early as 24h APF). Moreover, there is no solid evidence yet that connections form sequentially between the lamina and medulla, and medulla and lobula and lobula plate neurons. This description would need to be adjusted. A reference for the occurrence of spontaneous activity (prior to synapse formation) should be provided: Akin et al. 2019. This is important for the subsequent interpretation that cell death overlaps with "connectivity": if this term is used to refer to synapse formation, this may not hold, and need adjustment.

We are grateful for the Reviewer's feedback on this point. By connectivity, we mean the point when pre- and post-synaptic neurons make contact, regardless of whether synapses have formed, matured or are functional, or not. This is typically the final stage of targeting, following axon guidance. **We have now clarified the use of the term in the manuscript**, and we have edited the manuscript throughout taking care of these points.

- Next, the study assesses the impact of loss of Toll-2 in surviving lamina neurons L1 on columnar axonal branching. The authors observed that loss of Toll-2 or over-expression of DNT-2 leads to extension of axonal branches into neighboring columns in the medulla neuropil layer M1. Here, it would be important to assess, whether this phenotype is really due to the loss of Toll-2 or the fact that neighboring L1 neurons are missing (even just one neighbor missing may be enough to create this defect as contact-dependent repulsion may no longer work, Millard et al. 2007). Toll-2 MARCM clones would be able to address this point. Without further experiments conclusions need adjustments. Finally, the authors conclude that this is an indication for a role in connectivity, but indeed, it is not clear whether these projections lead to abnormal connections, as the thin projections may not form synapses. This statement would need to be adjusted or supported by including synaptic markers, such as Brp/or TransTango experiments.

The Reviewer raised two interesting points.
The first one is whether the phenotype in original Figure 5 directly reflects a Toll-2 dependent function or whether it is the indirect consequence of failed repulsion due to cells loss. Our data indicate that the abnormal contacts across columns cannot be explained by loss of contact-mediated repulsion due to cell loss, because we found the same phenotypes with loss of function for Toll-2 and gain of function for DNT-2 conditions. Loss of contact repulsion could explain Toll-2 knock-down data, but not DNT-2 over-expression data where neighbouring cells are not missing but in excess. Since both Toll-2- knock down and DNT-2 over-expression resulted in

© 2026. Published by The Company of Biologists under the terms of the Creative Commons Attribution License (https://creativecommons.org/licenses/by/4.0/).

the same phenotype, and Toll-2RNAi does not rescue the DNT-2 gain of function phenotype, the most parsimonious interpretation is that the levels of DNT-2 and the signalling events it induces through Toll-2 are instructive. This is also consistent with the function of mammalian neurotrophins, which are produced in limiting amounts to enable targeting and connectivity (Levi-Montalcini 1987 Science). And it is also consistent with our previous reports that both loss and gain of function for DNT-1, DNT-2, Toll-6 and -7 in embryos caused similar alterations in axon guidance (Zhu et al 2008 PLoS Biology; McIlroy et al 2013 Nature Neuroscience). Furthermore, Toll-2 knock-down resulted in two distinct phenotypes with equal penetrance (ie 50%), a severe phenotype where many L1 neurons were lost and a milder phenotype where L1 neurons remained. Similar phenotypes in columnar contacts were observed in both phenotypic classes, despite cells having very different cellular neighbours. As a result, we did not think MARCM clones would resolve whether a gradient of a neurotrophic factor produced in limiting amounts or contact repulsion is responsible for the observed phenotypes. **We thank this Reviewer for raising this point, and in response we have improved the discussion.**

The second point questions whether the abnormal columnar axonal branching establish aberrant connectivity patterns, leading to synapses. We tried to generate new data using the synaptic marker UAS-Denmark:sytGFP in L1 neurons, with the aim of seeing whether the abnormal axonal branches formed synapses upon *Toll-2RNAi* knock-down. However, unfortunately, this reporter did not work and we were unable to obtain further data to resolve this doubt. **In response to this criticism, we have now edited the manuscript text adjusting the claims and we mention that abnormal axonal extension are not necessarily functional.**

- The study concludes with the statement that over-expression of DNT-2 FL and knock-down of Toll-2 alters dendritic spine formation of lamina neurons L1 in the lamina, it increases by over-expression and decreases in Toll-2 knock-down. This is in contrast to the axonal projections, where additional extensions are observed in both genotypes. This should be discussed, as the opposite effects may be in line with an indirect effect through loss of neighbors in medulla columns (in contrast to the lamina).

We thank this Reviewer for this feedback, as it is a good point. **We have now added a comment on this point.**

- The authors conclude in the discussion (page 15 and 17) that DNT-2 by medulla neurons Mi1 or related medulla neurons is key to promoting survival of lamina neurons L1, expressing the Toll-2 receptor.
However, the manuscript does not provide any direct evidence that it is indeed Mi1 (or other medulla neurons) playing this role. Considering that DNT-2 may also be provided by photoreceptors (see above), this statement may need support by additional experiments, such as a Mi1 specific or photoreceptor- specific over-expression of DNT-2 in a DNT-2 whole animal mutant background and assessment of survival of labeled L1 neurons (e.g. using the markers Svp/Zfh1 (Xu et al. 2024). Moreover, it would be crucial to indeed determine the time point of when cells are undergoing apoptosis and to assess whether this would coincide with synaptogenesis. Such experiments would allow to comment on a potential retrograde signaling process between connecting neurons.

In response to this criticism raised by the Reviewer, **we now provide new evidence that DNT-2 is expressed in Mi1 neurons in the medulla and Toll-2 in L1 neurons.** The new data show:

- **Co-localisation of the Mi1 marker anti-Bsh in DNT-2+ neurons** visualised with DNT-2>6xmCherry. This demonstrates that DNT-2 is expressed in Mi1 neurons. **New Figure 3A-C.**

- **Co-localization of the L1 marker anti-Svp in L1 Toll-2+ neurons** labelled with Toll-2Gal4 UAShis- YFP, demonstrating that Toll-2 is expressed in L1 neurons. **New Figure 3D,E.**

- **Co-localization of the L3 marker anti-Erm in L3 Toll-2+ neurons** labelled with Toll-2Gal4 UAShis- YFP, demonstrating that Toll-2 is expressed in L3 neurons. **New Figure 3F,G.**

© 2026. Published by The Company of Biologists under the terms of the Creative Commons Attribution License (https://creativecommons.org/licenses/by/4.0/).

These new findings confirm our previous conclusion that DNT-2 promotes cell survival via Toll-2, based on the following data already present in the original submission:

(1) DNT-2 mutants have increased Dcp1+ apoptosis in the lamina
(2) DNT-2 over-expression decreased naturally occurring Dcp1+ cell death in the lamina
Together these two data sets show that DNT-2 is required to maintain cell survival in the lamina.
(3) Toll-2-RNAi knock-down increased Dcp1+ apoptosis in the lamina
(4) Over-expression of DNT-2 could not promote cell survival if Toll-2 was knocked-down
(5) Heterozygosity for Toll-2 induced lethality of otherwise viable *DNT-2*^{37}/*DNT-2*^{18} homozygous mutants.
(6) Over-expression of DNT-2 increased the number of Toll-2+ neurons

(7) *DNT-2*^{37}/*DNT-2*^{18} mutants lacked virtually all Toll-2+ neurons

Together, these data showed that DNT-2 promotes cell survival in the optic lobe via Toll-2 and that apoptosis in the absence of DNT-2 or Toll-2 functions leads to cell loss. The confirmation of the identity of DNT-2 neurons as Mi1 neurons and Toll-2+ neurons as L1 neurons makes our data stronger, and **we would like to thank this Reviewer for their helpful feedback.**

The timing of apoptosis during pupal development, with a peak at 24h APF and decay by the end of 48h APF was published by Togane et al 2012; Hara et al 2013. This is naturally occurring cell death, whereby many cells die during this phase in metamorphosis. We demonstrated already in the original version of the manuscript, Figure 3E,F that DNT-2/spz-5 and DNT-3/spz-3 are both required at this time point to maintain cell survival. This shows that the survival of cells in the optic lobe during naturally occurring cell death is actively maintained.

We now provide **new evidence showing that DNT-2 is expressed in the medulla concomitantly with Dcp1 during the wave of naturally occurring cell death** affecting both the medulla and the lamina.

These new data are provided in new Figure 5A.

We also **provide new data showing that DNT-1 Mi1 neurons connect to L1 neurons expressing the Toll- 2 receptor and that DNT-2 is required for these neurons to connect:**

- We over-expressed **Retrotango** (a tool to identify neuronal inputs of a candidate neuron) in DNT-2 neurons and identified lamina neurons. As we have now confirmed that DNT-2 is expressed in Mi1 neurons that are well-known to connect to L1 neurons, this confirms that DNT- 2 neurons connect to L1 neurons. Then we knocked-down DNT-2, and found that this reduced the number of L1 neurons revealed by Retrotango. **These data show that DNT-2 is required for Mi1 and L1 neurons to connect. These data are shown in new Figure 7A-C.**

Altogether, these new data confirm the identify of DNT-2 and Toll-2 neurons and have improved the demonstration that the neurotrophin DNT-2 regulates neuronal survival during neural circuit formation. We thank this Reviewer for their helpful input.

- The data and molecular and genetic methods are presented in detail in the Material and Method sections, as well as through pertinent supplementary tables. Similarly, the statistical analyses and sample numbers are indicated in the Material and Methods section and related supplementary tables. Table S4 does not indicate genotypes, but short versions of crosses, which should be corrected.
Moreover, the list of fly lines seems to miss one line (UAS-mCherry). Concerning the antibody list, sources are not provided (unlike stated in the main text). In the Result section, differences are described simply as statistically significant decreases and increases, however, at times it would be useful to add some indication about the level of differences (percentage or fold times).

Thank you very much for noticing these minor errors, which have now been improved.

Minor comments:

© 2026. Published by The Company of Biologists under the terms of the Creative Commons Attribution License (https://creativecommons.org/licenses/by/4.0/).

- The figures are informative, however, their orientations are not as indicated horizontal or lateral, but often oblique and not consistent (e.g. Fig 1a, last four panels). This makes comparisons more difficult. The authors could possibly find more suitable optical sections and present images more consistently.
Unfortunately, the PhD student who produced these data has now left the lab and repeating all of these experiments was not possible.

- Please provide the correct reference describing the occurrence of spontaneous activity (Akin et al. 2019).
Thank you, now done.

- The manuscript would be more easily accessible to non-experts, if schematic drawings would be provided (neuron subtypes, model).
Thank you, now done, where possible and reasonable to do so within the constraint of the figures.

- The final paragraph of the discussion about DNT-2 keeping connecting neurons together is not easily understandable, as the term "keeping together" remains undefined.
Thank you, now reworded.

Reviewer #3 (Significance (Required)):

As in the vertebrate nervous system, insect nervous systems rely on neurotrophins to control the survival and correct wiring of neurons. This study proposes a model, that advances our understanding as to how a specific neurotrophin, delivered by a post-synaptic neuron, acting via a specific receptor, controls several developmental steps of one of its pre-synaptic partners. The study assessed the expression patterns of three neurotrophins and the Toll receptor family, using the T2A-Gal4 expression system, revealing their dynamic expression in photoreceptors, specific lamina and medulla neurons, as well as tracheae and glia during pupal development. A strength of the study represents the identification of DNT-2 and Toll-2 as interacting partners, which could be assessed by the extensive set of specifically generated reagents for this study (Gal4 drivers and mutants). However, a number of statements may require additional experiments to conclusively support the proposed model, in particular the action of DNT-2 and Toll-2 in Mi1 and L1, respectively. Moreover, it would be important to critically assess the timing of events of cell death, setting up of projection patterns and synapse formation.

Audience: The outlined findings, if strengthened further, will be of interest for scientists studying nervous system development in vertebrates and invertebrates in general, and the action of neurotrophins in particular.

Thank you very much for the very positive assessment of our work and for considering our findings of interest to the broader neuroscience community.

Original submission to sister journal

First decision letter

MS Title: The neurotrophin DNT-2 via the Toll-2 receptor regulates neuronal survival and morphology during visual system development

Authors: Naser Alshamsi, Francisca Rojo-Cortes, Jervis Fernandes, Callum Perrett, Bangfu Zhu, Samaher Fahy, Guiyi Li, Anna Lassota, Marta Moreira and Alicia Hidalgo

I have now received all the referees' reports on the above manuscript and have reached a decision. I am sorry to say that the outcome is not a positive one. The referees' comments are appended below.

© 2026. Published by The Company of Biologists under the terms of the Creative Commons Attribution License (https://creativecommons.org/licenses/by/4.0/).

As you will see, the referees still have significant concerns about your paper, and are not strongly in favour of publication. Given their continuing concerns about the study, I must therefore, reject your paper. I do realise this is disappointing news, but this journal receives many more papers than we can publish, and we can only accept manuscripts that receive strong support from referees. I do hope you find the comments of the referees helpful, and that this decision will not dissuade you from considering us for publication of your future work.

Reviewer 1

Comments for the author

The authors investigate the role of neurotrophins in development of the Drosophila visual system. Using transgenic reporter lines, the authors show differential expression of four spz ligands and four Toll receptors in the optic lobe. They claim that two of these ligands, spz-3/DNT-3 and spz-5/DNT-2, are expressed in the optic lobe concurrently with a wave of cell death. The authors show that DNT-2 is expressed by Mi1 neurons and Toll-2 expressing cells in the lamina include L1 and L3 neurons. The authors claim that overexpression/loss of DNT-3 and DNT-2 is associated with decreased and increased cell death in the lamina respectively and suggest that DNT-3 promotes survival through Toll-8 whilst DNT-2 promotes survival through novel interactions with Toll-2. Finally, the authors claim that DNT-2 and Toll-2 regulate L1 neuron targeting and size.

Whilst the presentation of data has improved and additional evidence has been provided for some of the authors claims, there remain significant issues throughout which render the manuscript unsuitable for publication.

Major comments:

DCP-1 signal volume is an essential quantification for understanding the results of this study. Although the clarifications added to the methods partially address the disparity seen in DCP-1 signal volume between different experiments, they do not sufficiently address my concerns about the reliability of this quantification and this remains a major weakness in this study. This issue is particularly relevant to the experiments shown in 5H and 6C where the DCP1 signal volume is highly disparate between nsybGAL4 controls. Furthermore, while images have been included as evidence of Dcp1 signal in the lamina (Fig.5A), the absence of counterstains for lamina neuron markers in these images and subsequent DCP-1 signal volume quantifications makes these images difficult to interpret. In particular, counterstaining with lamina neuron and glia markers would allow the identity of DCP1+ cells to be identified more accurately.

The manuscript suggests a novel interaction between DNT-2 and Toll-2. Whilst DNT-2 gain of function experiments appear to support this relationship, direct, cell-type specific loss of function evidence of this relationship is limited. This manuscript presents evidence that loss of function of Toll-2 and DNT-2 both lead to increased cell death in the lamina and that Toll-2+ cells are lost from the lamina, medulla and lobula complex in whole animal DNT-2 mutants. However, evidence that DNT-2 specifically acts through binding Toll-2 on these neurons is absent. It remains possible that DNT-2 supports the survival of Toll-2+ neurons in the lamina through other Toll receptors, or through intermediary cell types such as glia. Further experiments to prove the specificity of the requirement of DNT-2 for the survival Toll-2+ neurons are required. Critically, The authors conclude that DNT-2/DNT-3 promote survival specifically through Toll-2/Toll-8 respectively. Testing whether loss of function of DNT-2 and DNT-3 specifically leads solely to loss of neurons which express the relevant Toll receptors and not to loss of neurons expressing other Toll receptors would strengthen the claim that these ligands promote survival through direct interactions with specific receptors. Additionally, DNT mutants are not compared against heterozygous controls.

Whilst the GRASP experiment provides evidence of connections between DNT-2 and Toll-2 neurons, the images shown in 4A and 4B are in different focal planes which makes comparison impossible.

One of the key potential findings suggested by this manuscript is a model where Mi1 neurons in the medulla secrete DNT-2 which binds to Toll-2 on L1 and L3 lamina neurons to promote survival, connectivity and morphology. However, whilst the phenotypes observed in whole animal DNT-2 mutants and Toll-2RNAi in L1 neurons are suggestive of this model, the requirement for secretion of

© 2026. Published by The Company of Biologists under the terms of the Creative Commons Attribution License (https://creativecommons.org/licenses/by/4.0/).

DNT-2 specifically from Mi1 neurons to L1/L3 neurons remains untested. Furthermore, the images of L1 neuron misrouting remain unconvincing. In particular, The quality of the 24B10 staining is variable and is particularly poor in 7G, calling into question the accuracy of these quantifications. Moreover, brightness of both green and magenta signals is clearly different between these images (5E' vs 5I').

Crucially, in Figure 8, the authors conclude that expressing Toll-2RNAi in L1 neurons simultaneously with DNT-2FL (8D) "prevented the increase in dendritic size that would have been caused by DNT-2FL over-expression". This conclusion is problematic as it relies on a comparison between results from experiments driving different GFP reporter constructs (8C vs 8D; CD8GFP vs myrGFP). Indeed, the signal volume for the control with mCD8GFP (~25,000 units) is considerably lower than the signal volume for the control with myrGFP (~60,000 units). Without assessing the effect of expressing DNT-2FL in L1 neurons using myrGFP as a reporter, the authors cannot assume any effect that "would have been caused by DNT-2FL over-expression" on myrGFP signal volume. In addition, a titration control is critical to make any comparison between expressing Toll-2RNAi and DNT-2FL and DNT-2FL alone as a failure to increase signal volume in the former may result from reduced expression of DNT-2FL due to titration rather than due to the effect of Toll-2RNAi.

In addition to these issues, there remain significant issues with the presentation of the figures. In figure 1, the inconsistent orientations and N-cadherin counterstains along with the absence of timepoints in some panels makes the figure difficult to interpret. Furthermore, it is not clear which panels in figure 2 (E-L) are clones stained for anti-HA and which are expressing GFP reporters. This is exacerbated by a description "(E-K) MCFO clones with anti-HA antibodies (E, F, G, I, J, K,L)" which appears to contradict the genotypes listed in the legend. Another issue with the manuscript is that the developmental stage is often not clearly indicated in figure, legend or results. This may seem like a minor comment but is essential information for interpreting results in multiple figures.

Minor comments:

Line 115: should this say central brain neurons?

Line 246: szp-5 typo

-Line 289: "Most dramatically, DNT-218/DNT-237 null mutants revealed a complete loss of Toll-2+ cells throughout the optic lobe" - Untrue, there remain Toll-2+ cells of ambiguous identity in the optic lobe.

Diagrams in figures not adequately explained.

Figure 7A: Unclear what normal and weak refer to in the control

Figure 7C: lamine typo, Y axis should include RetroTango+ before lamina neurons so as not to imply that almost all lamina neurons are lost

-No counterstain to confirm that DNT-2 is expressed in neurons in the retina

Figure S7 plots expression profiles for multiple cell types not mentioned in this study which make the graphs difficult to read even in a digital version of this figure.

Reviewer 2: SUMMARY OF THE ADVANCE MADE IN THIS PAPER AND ITS POTENTIAL SIGNIFICANCE TO THE FIELD

The revised manuscript of Naser Alshamsi and colleagues in the Hidalgo lab has significantly gained from the extensive revisions. In particular, the additional experimental evidence that indeed lamina neurons L1 and medulla intrinsic Mi1 neurons are included in the Toll-2 receptor and DNT-2 neurotrophin expressing neuron populations strengthens the argument that this signaling pathway could be involved in mediating survival, as well as lateral axonal branch extension and dendrite formation of the presynaptic partner. The authors generally addressed my comments. However, the manuscript still remains rather difficult to understand and some imprecisions and urgent errors need to be addressed.

© 2026. Published by The Company of Biologists under the terms of the Creative Commons Attribution License (https://creativecommons.org/licenses/by/4.0/).

SUGGESTIONS TO AUTHORS

1. Read-out of T2A-Gal4 lines: the term of « spatial distribution of translated transcripts » does not seem helpful. Regardless of how this technology works, the revealed expression pattern will depend on the reporter, revealing only the identity of neurons producing the DNTs. The description should thus be adjusted.
2. Expression of DNT-2 and DNT-3 in the retina: mAb24B10 and anti-Elav immunolabeling in the Drosophila retina normally work very faithfully, and should lend itself readily for co-labeling. However, as the authors encountered technical problems, they propose, based on already existing images, that labeled cells correspond to pigment cells and interommatidial bristle neurons. Please note that the latter have distinct projections extending into the suboesophageal ganglion and could be identified on this morphological criterion to remove some of the speculative interpretations, especially as they are later mentioned again as potential source of DNT-2 (albeit a less important one for the discoveries presented in this manuscript).
3. The revised Figure 2A now contains 2 identical images described as 48 hours APF and 72 hours APF (it is 72h APF), this needs to be urgently corrected.
4. The orientations and planes of images shown are still highly variable, making it very difficult to identify neuron subtypes (e.g. in Figure 1E-G it is not possible to identify the cell bodies as lobula complex neurons).
5. Line 135 should be Toll-1-T2A>myrGFP
6. Figure 2F and J (left) show identical neurons. The right hand in panel J does not allow to identify the neuron as L2.
7. Mi1 neurons are normally present in every medulla column, this is not well reflected in Figure 3A because of the unusual angle of the image (it would be much better in the angle shown in Figure 1D for example. The developmental stages of panels are not indicated. The sizes of the scale bars need adding in the legend, and it should read « colocalise with » and anti-Svp. Furthermore, Figure 2I illustrates expression in Mi1 neurons, this is however only explored in Figure 3, where the cartoon would be placed more logically.
8. GRASP labeling - the developmental stage is not indicated in Figure 4 and it is not clear what the arrows are pointing to. If the stage is very young (first half of pupal development), no synapses are formed yet, and thus describing the signal as revealing connections is not appropriate, it can solely indicate proximity (one of the limitations of GRASP). Whether interommatidial bristle neurons are in contact with lamina neurons remains speculative in light of the provided data.
9. Line 290. The authors report a complete loss of Toll-2 neurons in DNT2 mutant flies. Yet while there is significant loss in the relevant panels in Figure 6D on the left-hand side, there are still neurons surviving, but the magnified panel chose an area with no cell bodies remaining. « Complete » should thus be replaced by a less strong description.
10. In the revised manuscript, the authors still use connectivity in a not much clearer way compared to the earlier version of the manuscript. Moreover, Retrotango is not set up to reveal requirements during development, but solely reveals the outcome in adult brains. In line 326, the authors focus on the connections between Mi1 and L1 neurons, however, the Retrotango labeling solely shows that some lamina neurons are labeled and thus could be presynaptic to DNT-2 neurons, which could include Mi1 interneurons in the medulla. All this should be described in a more careful way.
11. While it is very likely that there is an impact on L1 axonal branch extensions into neighboring columns following Toll-2 RNAi knockdown it is not possible to exclude that this is not an indirect effect of loss of neighboring neurons, solely MARCM could do so. The comparison of samples with mild and strong phenotypes could be an indication, however as the quantifications of defects does not provide numbers of L1 axons counted to establish percentages (only the number of brains is provided), it remains unclear, how to interpret the data, and thus, careful wording remains essential.

Transfer to Biology Open

<u>First decision letter</u>

© 2026. Published by The Company of Biologists under the terms of the Creative Commons Attribution License (https://creativecommons.org/licenses/by/4.0/).

MS ID#: bio.062837

MS Title: The neurotrophin DNT-2 via the Toll-2 receptor regulates neuronal survival and morphology during visual system development

Authors: Naser Alshamsi, Francisca Rojo-Cortes, Jervis Fernandes, Callum Perrett, Bangfu Zhu, Samaher Fahy, Guiyi Li, Anna Lassota, Marta Moreira and Alicia Hidalgo

I am happy to tell you that your manuscript has been accepted for publication in Biology Open, pending our standard publication integrity checks. It was accepted on 29th July 2026.

© 2026. Published by The Company of Biologists under the terms of the Creative Commons Attribution License (https://creativecommons.org/licenses/by/4.0/).
